# A Coumarin–Porphyrin FRET Break-Apart Probe for Heme Oxygenase-1

**DOI:** 10.1021/jacs.0c12864

**Published:** 2021-04-13

**Authors:** Edward R. H. Walter, Ying Ge, Justin C. Mason, Joseph J. Boyle, Nicholas J. Long

**Affiliations:** † Department of Chemistry, Imperial College London, Molecular Sciences Research Hub, White City Campus, Wood Lane, London W12 0BZ, U.K.; ‡ National Lung and Heart Institute, Imperial College London, Du Cane Road, London W12 0NN, U.K.

## Abstract

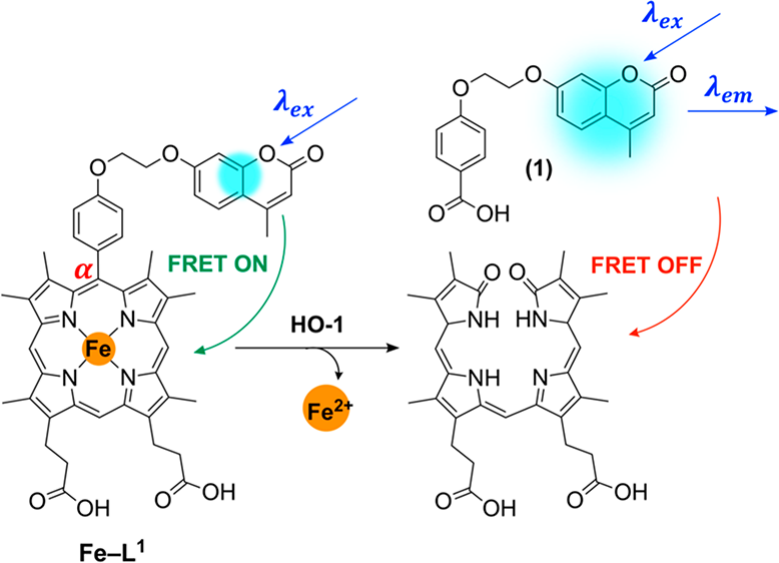

Heme oxygenase-1 (HO-1) is a vital enzyme in humans that primarily regulates free heme concentrations. The overexpression of HO-1 is commonly associated with cardiovascular and neurodegenerative diseases including atherosclerosis and ischemic stroke. Currently, there are no known chemical probes to detect HO-1 activity, limiting its potential as an early diagnostic/prognostic marker in these serious diseases. Reported here are the design, synthesis, and photophysical and biological characterization of a coumarin–porphyrin FRET break-apart probe to detect HO-1 activity, **Fe–L**^**1**^. We designed **Fe–L**^**1**^ to “break-apart” upon HO-1-catalyzed porphyrin degradation, perturbing the efficient FRET mechanism from a coumarin donor to a porphyrin acceptor fluorophore. Analysis of HO-1 activity using *Escherichia coli* lysates overexpressing hHO-1 found that a *6-fold* increase in emission intensity at 383 nm was observed following incubation with NADPH. The identities of the degradation products following catabolism were confirmed by MALDI-MS and LC–MS, showing that porphyrin catabolism was regioselective at the α-position. Finally, through the analysis of **Fe–L**^**2**^, we have shown that close structural analogues of heme are required to maintain HO-1 activity. It is anticipated that this work will act as a foundation to design and develop new probes for HO-1 activity in the future, moving toward applications of live fluorescent imaging.

## Introduction

**Figure 1:**
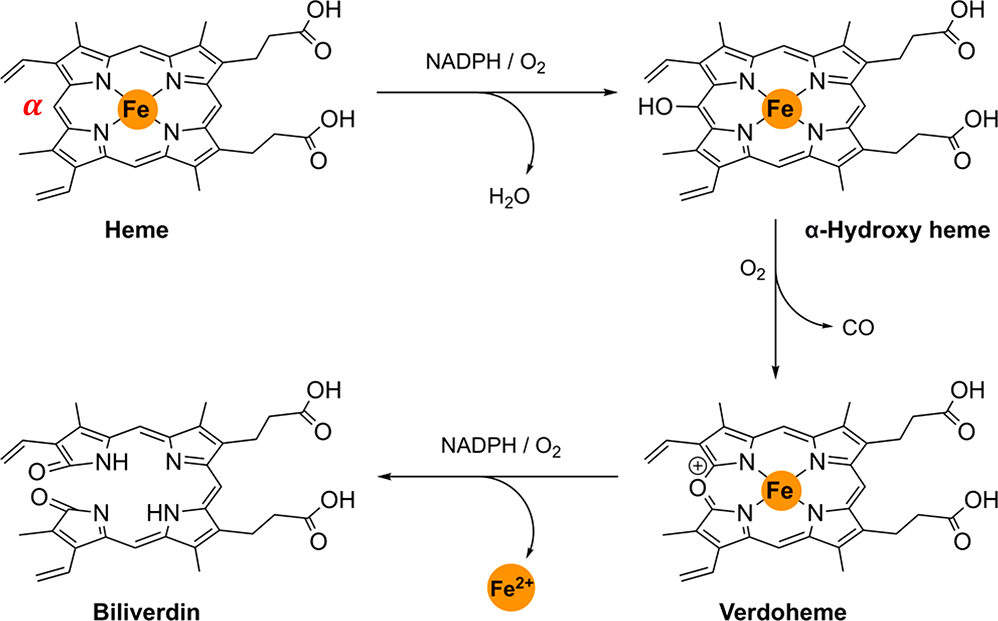
HO-1 catabolism of “free” heme.

Heme oxygenase (HO) is an important homeostatic microsomal enzyme in vascular biology and cell signaling. There are two active mammalian isoforms, namely HO-1 and HO-2, encoded by the *HMOX1* and *HMOX2* genes, respectively. The primary role of HO is to prevent the accumulation of cytotoxic “free” heme (Fe-PPIX),^1,2^ which has the potential to act as a Fenton catalyst *in vivo*, leading to the generation of reactive oxygen species. (The term “free” denotes heme that is not bound to proteins, either because it is newly synthesized and not yet incorporated into hemoproteins or it has been released from hemoproteins during oxidative stress.^68^) Heme catabolism is a three-step reaction that requires molecular oxygen, NADPH, and cytochrome p450 reductase (Figure 1).^3,4^ During the process, the porphyrin ring is regioselectively decyclized at the α-carbon atom to form α-biliverdin, with the loss of carbon monoxide (CO) and ferrous iron (Fe^2+^). Biliverdin is further converted to bilirubin by biliverdin reductase (BVR) in the cytosol.^5^ Both isoforms catalyze heme degradation.^6^ However, HO-1 is the only isoform that is induced by cellular stress stimuli in the body and has a defensive role in a number of diseases including atherosclerosis^7,8^ and some cancers.^9−11^

Each of the byproducts of heme degradation is known to be cytoprotective. For example, Fe^2+^ is chelated by ferritin, where it is safely stored out of Fenton activity, and a number of studies have demonstrated that CO is an essential intracellular signaling molecule, activating the *K*_ATP_ ion channel^12^ and suppressing endothelial cell apoptosis.^13,14^ Finally, bilirubin has also been proven to display antioxidant properties.^15^ Individuals with bilirubin concentrations above basal levels, in genetic disorders such as Gilbert syndrome, are significantly less likely to develop cardiovascular disease over their lifetime.^16^

HO-1 plays a protective role in atherosclerosis, a leading cause of death in the western world. Defined by the gradual buildup of plaque on the artery walls, atherosclerosis has no symptoms in its mild form but, dangerously, over time can lead to the onset of ischemic stroke^17,18^ and coronary artery disease.^19^ In atherosclerosis, HO-1 is induced during intraplaque hemorrhage (IPH), a rupture of the vulnerable plaque coating the artery walls. IPH is an atheroaccelerant, releasing cholesterol-enriched membrane lipids and hemoglobin into the plaque interior.^20^ In turn, IPH leads to the development of more advanced vulnerable plaques and can further lead to the development of blood clots, restricting the blood flow to the heart and the brain.

Fluorescence imaging is a powerful, noninvasive imaging modality used in cell biology.^21−23^ Its high sensitivity and spatial resolution allow for the detection of a variety of biological targets.^24,25^ Fluorescence resonance energy transfer (FRET) is a distance-dependent dipolar interaction between two fluorophores that can unlock detailed information on the nanometer scale.^26^ The process is characterized by a nonradiative energy transfer from a donor fluorophore to an acceptor, providing there is a good spectral overlap between the donor emission and acceptor absorbance spectrum and they are close enough in space.

Over the years, FRET probes have been designed to detect numerous biological processes including protein folding,^26^ labile pools of biologically relevant metal ions,^27,28^ peroxynitrate anions,^29^ and “free” heme.^30−32^ The biosensing of enzyme activity by ratiometric “break-apart” probes has enabled enzyme activity to be detected in real-time.^33−35^ Many of these probes are peptide-based, sensing cutting enzymes such as caspase-3,^36^ and recently, an alternative method has been developed enabling a “labeling after recognition” strategy to be utilized effectively.^37^

There are no known chemical probes to detect HO-1 enzyme activity. Current enzyme assays are long and cumbersome processes, and most do not allow real-time kinetic measurements. The development of a fluorescent chemical probe to detect HO-1 will, therefore, significantly move the field forward. Such an advancement will enable the detection of IPH and potentially plaque instability, often the portent of plaque rupture and severe associated conditions.

Recently, coumarin-porphyrin diads have gained an increasing interest, displaying a high FRET efficiency between the coumarin donor and the porphyrin acceptor fluorophores. In 2011, Lin and co-workers reported a ratiometric coumarin–porphyrin probe for biologically relevant thiols. For example, cysteine cleaved the disulfide bond linking the two fluorophores, resulting in a 60-fold increase in the coumarin emission intensity.^38^ In contrast, Liu and co-workers developed a Zn–coumarin–porphyrin FRET probe based on 5-(4-hydroxyphenyl)-10,15,20-triphenyl porphyrin (HP-TPP) and monitored the binding properties to DNA.^39^

**Figure 2:**
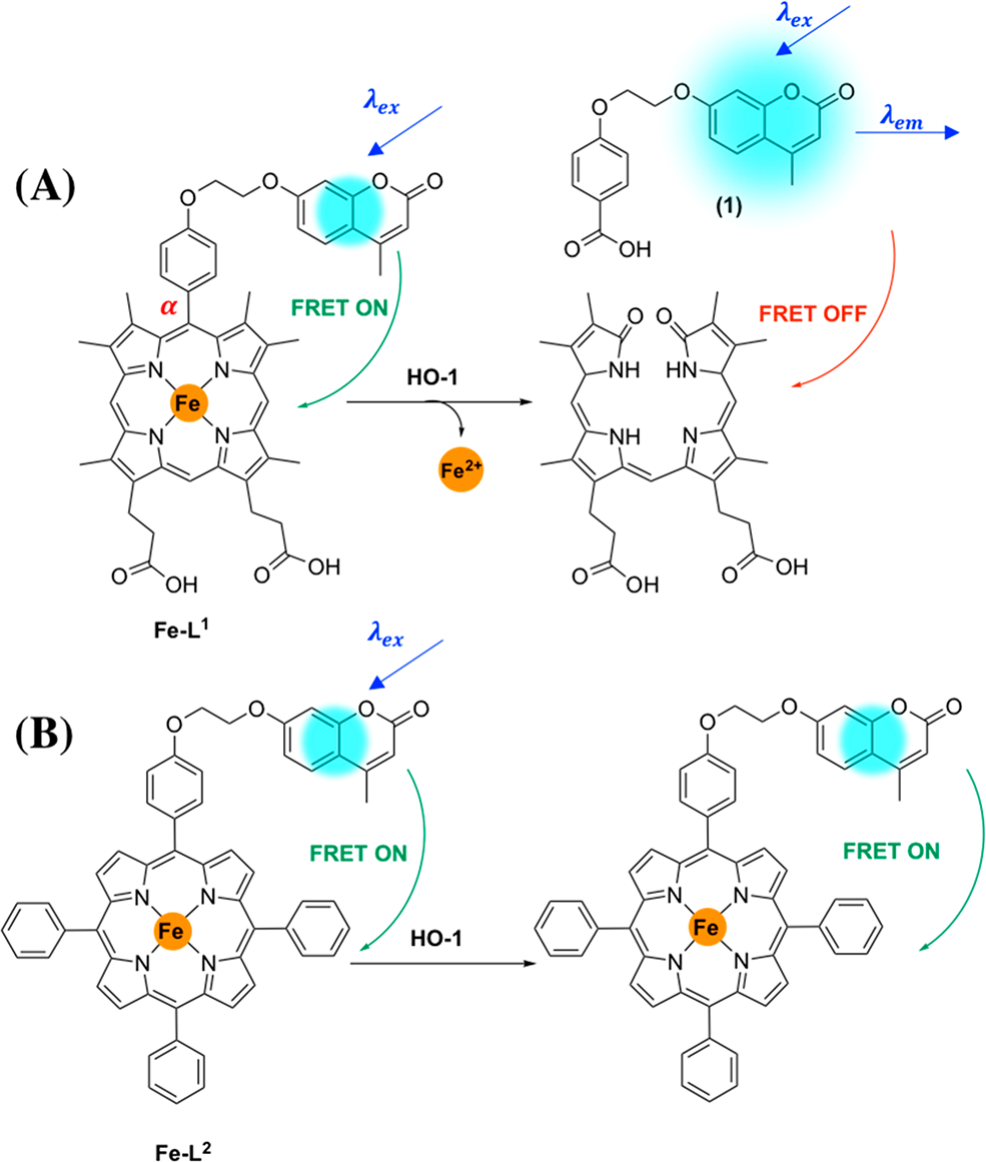
(A) Rationale behind **Fe–L**^**1**^ probe design and the formation of 4-(2-((4-methyl-2-oxo-2*H*-chromen-7-yl)oxy)ethoxy)benzoic acid (**1**) following porphyrin catabolism. (B) **Fe–L**^**2**^ was not expected to be a substrate for HO-1.

Here, we report the design, synthesis, photophysical, and biological characterization of a “turn-on” coumarin–porphyrin FRET probe for HO-1, namely **Fe–L**^**1**^ (Figure 2). Complex **Fe–L**^**1**^ is based on the structure of dimethyldeuteroheme (Fe–DMD), a symmetrical analogue of Fe–PPIX, known to display an activity toward HO-1.^40^ The “turn-on” fluorescent probe was designed to exploit the regioselective α-cleavage of Fe-PPIX observed during HO-1 catabolism. In this process, the α-carbon atom linking the coumarin donor and porphyrin acceptor fluorophores is lost during porphyrin decyclization and the FRET process is switched off, resulting in an increase in the fluorescence emission intensity of the coumarin donor fluorophore (Figure 2A). A 7-hydroxymethylcoumarin moiety was selected as the donor fluorophore, due to the possibility of functionalization onto a porphyrin scaffold and the excellent spectral overlap with the absorbance spectrum of porphyrin-based compounds (Figures S1 and S2). Additionally, 7-hydroxymethylcoumarin derivatives are also known to be fairly photostable,^41,42^ including compound **1** (Figure S3), a characteristic that will importantly maximize the observed increase in fluorescence following enzymatic probe cleavage.

The mechanism of **Fe–L**^**1**^ α-cleavage is expected to be analogous to that of α-*meso* phenylheme—where regioselective decyclization occurs at the α-carbon atom to form benzoic acid and biliverdin.^43,44^ HO-1 catabolism of α-*meso* phenylheme occurs via a slightly different pathway from that of Fe-PPIX (Figure 1) and without the loss of CO.^43^ The exact mechanism is still not completely understood, but it is thought to proceed via an isoporphyrin intermediate.^44^ In the case of **Fe–L**^**1**^, the formation of 4-(2-((4-methyl-2-oxo-2*H*-chromenn-7-yl)oxy)ethoxy)benzoic acid (**1**) is likely to be responsible for the fluorescence “turn-on” following porphyrin catabolism (Figure 2A).

We herein describe a comparison of enzyme activity of two porphyrin-coumarin diads **Fe–L**^**1**^ and **Fe–L**^**2**^ toward HO-1 catabolism. An *E. coli* lysate system overexpressing human HO-1 (hHO-1) was selected to act as a proof-of-concept model. Complex **Fe–L**^**2**^ is based on the structure of HP-TPP, and the subsequent Zn-complex has been previously synthesized by Liu and co-workers.^39^ In our study, the Fe complex was of greater interest, however, as Zn-porphyrins such as zinc protoporphyrin (ZnPP) are known to inhibit hHO-1 activity.^45^ As the structure of **Fe–L**^**2**^ differs significantly from biologically relevant Fe-PPIX, it will provide an additional insight into the structural requirements to maintain HO-1 activity. It was expected that unlike **Fe–L**^**1**^, **Fe–L**^**2**^ would not act as a substrate for HO-1 and would be used as a control (Figure 2B).

## Results and Discussion

### Design, Synthesis and Characterization of **Fe–L**^**1**^ and **Fe–L**^**2**^

Over the years, numerous analogues of protoporphyrin IX (PPIX) have been synthesized for applications from photosensitizers in photodynamic therapy (PT)^46^ to multiporphyrin arrays.^47^ Functionalization is commonly achieved at two main positions, first, via one or both of the vinyl positions through Diels–Alder^48^ or bromination^49^ reactions. Another route of functionalization is through amide coupling methodologies of one (or both) of the propionic acid residues.^50^ Such reactivity has been summarized in an excellent review by Senge and Sitte.^51^ However, it still remains a major challenge to functionalize the α-*meso*-position of PPIX selectively, as each of the four inequivalent *meso*-positions have an almost identical reactivity leading to a mixture of species that are difficult to separate.^52,53^

Instead of selectively functionalizing the α-*meso*-position of PPIX, we designed a porphyrin probe based on Fe–DMD, **Fe–L**^**1**^ (Figure 2). Similar to Fe–PPIX, Fe–DMD is a known substrate for HO-1.^40^ Importantly, the increased porphyrin symmetry—achieved by exchanging two vinyl-substituents with two methyl—allows a phenol linker between the coumarin and porphyrin FRET pair to be introduced.

**Scheme 1:**
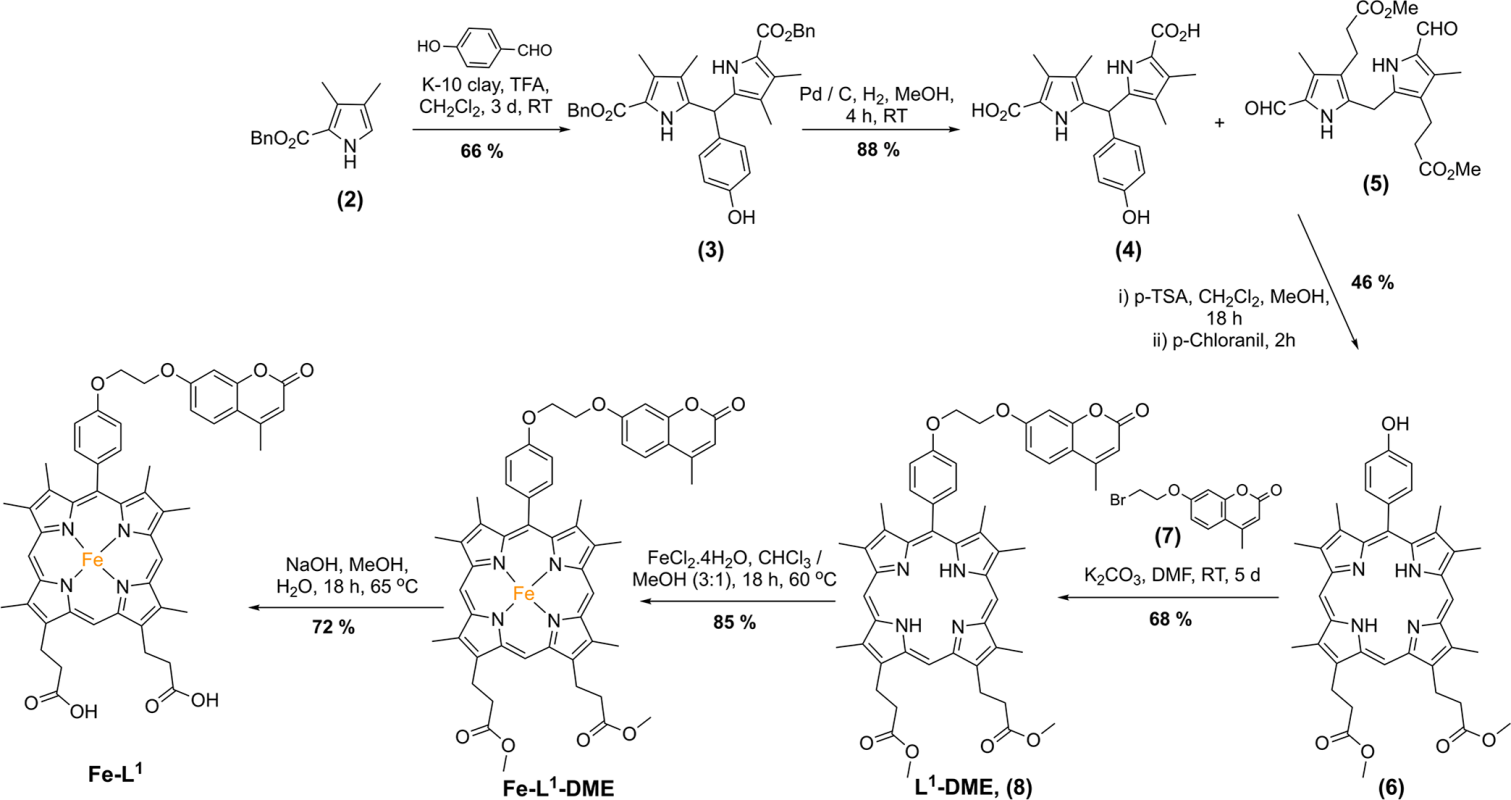
Synthesis of **Fe–L**^**1**^

The synthetic route toward **Fe–L**^**1**^ is shown in Scheme 1. Briefly, the synthesis began with benzyl 3,4-dimethyl-1H-pyrrole-2-carboxylate **(2)**, first reported by Lash and co-workers in 1994.^54^ Condensation of **2** with 4-hydroxybenzaldehyde in dichloromethane and trifluoracetic acid formed **3** in moderate yield following purification by column chromatography. Subsequent benzyl ester hydrogenation and MacDonald-type condensation^55^ with diformyl dipyrromethene **5**(56) produced **6**. Alkylation of **6** with **7** was achieved in DMF at room temperature over 5 d with potassium carbonate as the base in a procedure adapted from Wang and Liu.^39^ Complexation of **8** with FeCl_2_·4H_2_O in chloroform/methanol (3:1) and methyl ester hydrolysis under basic conditions gave rise to the formation of complex **Fe–L**^**1**^. Ester hydrolysis after metal complexation was found to be much more effective. Complexation of the free-acid porphyrin was difficult to achieve, due in part to its poorer solubility in organic solvents and purification difficulties via column chromatography. No demetalation was observed during the ester hydrolysis of **Fe–L**^**1**^**–DME**, with only the desired product present in the MALDI-MS.

Complex **Fe–L**^**2**^ was synthesized as a control to **Fe–L**^**1**^ for the biological characterization assays. The synthesis of **Fe–L**^**2**^ is based on an alkylation reaction of HP–TPP **9** with coumarin **7** under conditions identical to those reported for the alkylation of **6** (Scheme S1).

HO-1 catabolism of **Fe–L**^**1**^ is predicted to produce coumarin **1** as a byproduct accompanying biliverdin and Fe^2+^. In order to confirm the identity of the coumarin-based degradation product formed during porphyrin catabolism, **1** was synthesized in a two-step procedure from ethyl 4-hydroxybenzoate (Scheme S2). Coumarin methyl ester **12** was also synthesized and fully characterized to assist the mass spectrometry analysis of the *E. coli* lysates.

### Photophysical Studies of **Fe–L**^**1**^ and **Fe–L**^**2**^

The photophysical properties of porphyrin compounds have been studied in great detail due to their widespread application as photosensitizers for photodynamic therapy,^57−59^ chemical probes, and organic light-emitting devices (OLEDs).^60^ The photophysical properties of the two coumarin–porphyrin ligands and their subsequent Fe^2+^ complexes in this study were recorded in aqueous solutions of PBS buffer at pH 7.4 in order to mimic physiological conditions (Table 1). For comparison, photophysical characterization was also performed in chloroform and is detailed in the Supporting Information (Table S1). It was found in both PBS buffer and chloroform that all coumarin–porphyrin diads display excellent FRET efficiencies, estimated at greater 95% (Figures S4 and S5, Table 1, and Table S1). Such a high efficiency supports the literature precedent of an efficient intramolecular energy transfer between coumarin donor and porphyrin acceptor fluorophores.^38,39^

**Table 1:** Photophysical Data for **1**, **7**, and Coumarin–Porphyrin Diads Discussed in This Study^a^

	λ_abs_ (nm) (ε [10^4^ M^–1^ cm^–1^])					
compd/complex	UV	Soret	Q-band	emission λ_max_ (nm)	*E* (%)	ϕ_514nm_(%)
(1)	320 (1.5)			384		e
(7)	320 (1.5)			383		e
L^1^–DME	320 (1.0)	413 (1.6)	b	387,^c^ 638, 675, 708	95.1	0.5
Fe–L^1^–DME	320 (2.2)	403 (2.5)	b	387^c^^,^^d^	99.2	e
Fe–L^1^	321 (2.2)	401 (3.0)	b	383^c^^,^^d^	99.6	e
L^2^	294 (0.6)	430 (2.7)	527 (0.4), 565 (0.2), 603 (0.2), 661 (0.1)	383,^c^ 660, 727	96.8	2.0
	320 (0.7)					
Fe–L^2^	320 (1.2)	417 (1.5)	587 (0.8), 629 (0.7)	383^c^^,^^d^	99.4	e

a Concentration = 20 μM in PBS buffer (pH = 7.4), λ_ex_ = 320 nm, 298 K. Quantum yields (ϕ) ± 20% were measured using tetraphenylporphyrin (TPP) in toluene (ϕ_514nm_ = 0.11) as the standard.^62^

b Broad Q-bands.

c Residual coumarin emission.

d No porphyrin emission was observed.

e No quantum yield was measured.

The absorbance spectra of **Fe–L**^**1**^ and **Fe–L**^**2**^ in PBS buffer and chloroform are shown in the Supporting Information (Figures S6 and S7). In PBS buffer, **Fe–L**^**1**^ displayed a broad Soret band centered at 401 nm and a broad Q-band stretching from 541 to 680 nm assigned to S_0_ → S_2_ and S_0_ → S_1_ transitions, respectively. The absorbance at 320 nm was assigned to the coumarin moiety. Biologically relevant analogues **L**^**1**^ and **Fe–L**^**1**^ displayed a significantly broader absorbance spectrum in aqueous media compared to that in chloroform. Such a phenomenon is typical of PPIX analogues, due to the increase in π-stacking, and is strongly dependent on pH and ionic strength.^61^ As such, slight fluctuations in the absorbance spectra were observed for **Fe–L**^**1**^ with pH. However, very little change in the absorbance or emission spectra were observed for the expected break-apart product (**1**) from pH 6–10 (Figure S8).

In contrast to **L**^**1**^, analogues of HP–TPP, **L**^**2**^ and **Fe–L**^**2**^, displayed sharper absorbance spectra in PBS buffer, and red-shifted Soret bands at 430 and 417 nm, respectively (Figure S6). In both Fe-based porphyrins, complexation of Fe^2+^ was confirmed by the change in the number of Q-bands in the UV–vis spectrum in chloroform–from four to two. A blue-shift in the Soret band was also observed in aqueous and organic media. Complex **Fe–L**^**1**^**–DME**, for example, displayed a 10 nm hypsochromic shift in PBS buffer after Fe^2+^ complexation (Table 1).

The excitation spectra of **L**^**1**^ and **L**^**2**^ are shown in Figures S9 and S10 and further validate the presence of FRET from coumarin to porphyrin fluorophores. Similar to the absorbance spectra, in PBS buffer **L**^**1**^ displayed a broad response, even more so than that of the absorbance spectrum. It was also noted that a split Soret band was present in chloroform for both **L**^**1**^ and **L**^**2**^. A peak centered at 320 nm, corresponding to the coumarin donor moiety, was displayed in each instance.

**Figure 3:**
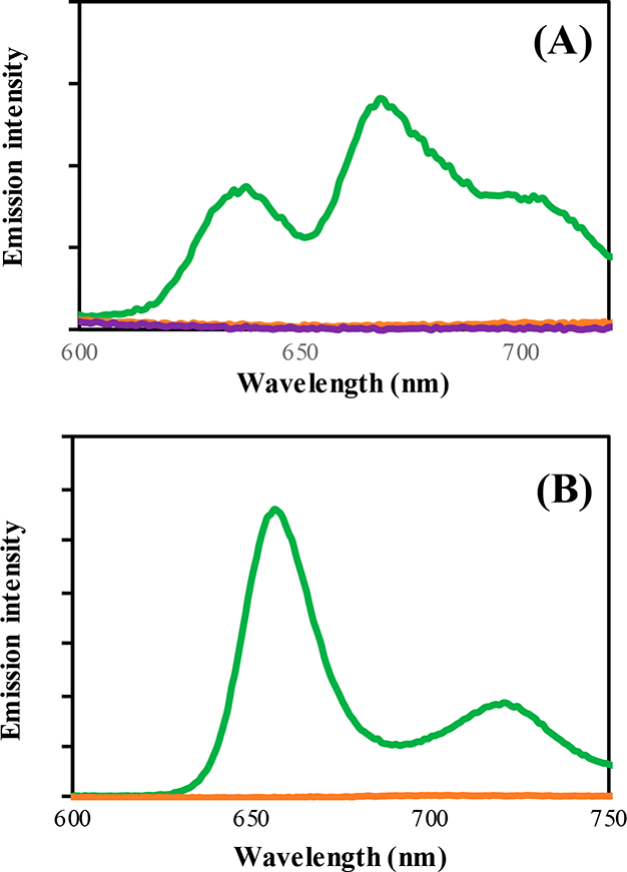
Emission spectra of (A) **L**^**1**^**–DME** (green), **Fe–L**^**1**^**–DME** (orange), **Fe–L**^**1**^ (purple) and (B) **L**^**2**^ (green), **Fe–L**^**2**^ (orange). Concentration = 20 μM in PBS buffer pH = 7.4, λ_*ex*_= 320 nm, 298 K. Slits 10:10.

The emission spectra of **L**^**1**^ and **L**^**2**^ analogues are displayed in Figure 3 and Figure S11 in PBS buffer and chloroform, respectively. Following excitation at λ_max_ of the coumarin moiety (320 nm), characteristic porphyrin emission spectra was observed in free base analogues **L**^**1**^**–DME** and **L**^**2**^. However, a rather different emission profile was observed in each case. In PBS buffer, **L**^**1**^**–DME** displayed two main peaks at 638 and 675 nm with a broad shoulder at 708 nm (Figure 3A). On the other hand, **L**^**2**^ presented a spectrum with two distinct emission peaks at 660 and 727 nm (Figure 3B). The difference in spectral shape is likely to be due to π-stacking in aqueous media and is not observed in chloroform where both spectra have the same emission profile. In both PBS buffer and chloroform, **L**^**2**^ displays a red-shifted emission compared to **L**^**1**^**–DME** due to the increased conjugation provided by the three additional *meso*-phenyl substituents.

Following Fe^2+^ complexation of the free-base analogues, porphyrin emission was completely quenched in PBS buffer and chloroform (Figure 3 and Figure S11). Such behavior is not surprising, as Fe^2+^ is well-known to quench fluorescence through electron and/or energy transfer processes.^63^ Complexation of Fe^2+^ also significantly quenches residual coumarin emission. Therefore, prior to porphyrin catabolism by hHO-1, no porphyrin and only weak coumarin fluorescence was observed.

### Determining the HO-1 Activity

To determine whether the coumarin–porphyrin diads **Fe–L**^**1**^ and **Fe–L**^**2**^ act as substrates for HO-1, we incubated the two probes with an *E. coli* lysate overexpressing hHO-1. *E. coli* systems have been shown to support recombinant HO-1 function in the absence of cytochrome p450 reductase (CPR), with the role of CPR likely substituted by flavodoxin and flavodoxin reductase.^64^ Such systems are an ideal proof-of-concept model due to a high level of hHO-1 expression and have been used effectively by Sigala and co-workers to probe His-Heme ligation in HO catalysis.^65^ In mammalian systems, biliverdin is subsequently converted to bilirubin by biliverdin reductase (BVR); however, *E. coli* BL21 does not express BVR. Therefore, the biliverdin product formed from **Fe-L**^**1**^ decyclization is expected to accumulate.

Complexes **Fe–L**^**1**^ and **Fe–L**^**2**^ were incubated at 37 °C (310 K) for 16 h with *E. coli* lysate fractions and 1 mM NADPH, a required substrate of HO-1 catalyzed heme degradation. Such concentrations of NADPH are higher than physiological levels but were chosen to ensure maximum possible reaction conversion for our novel design. In order to quantify HO-1 activity, a control experiment was carried out where **Fe–L**^**1**^ and **Fe–L**^**2**^ were mixed with an equal volume of *E. coli* lysate without addition of NADPH and without incubation. UV–vis, fluorescence spectroscopy, and mass spectrometry analysis of the bacterial lysate systems were used to monitor the HO-1-catalyzed porphyrin degradation of **Fe–L**^**1**^ and **Fe–L**^**2**^.

Probe concentrations of 50 μM were chosen for the UV–vis and fluorescence analysis of the lysates. Additionally, the emission intensity of **1** was found to decrease at concentrations above 50 μM (Figure S12). However, it was determined that probe concentrations of 50 μM were too dilute to obtain accurate MS analysis. Instead, concentrations of 200 μM were used for the mass spectrometry analysis.

#### UV–vis Spectroscopy

**Figure 4:**
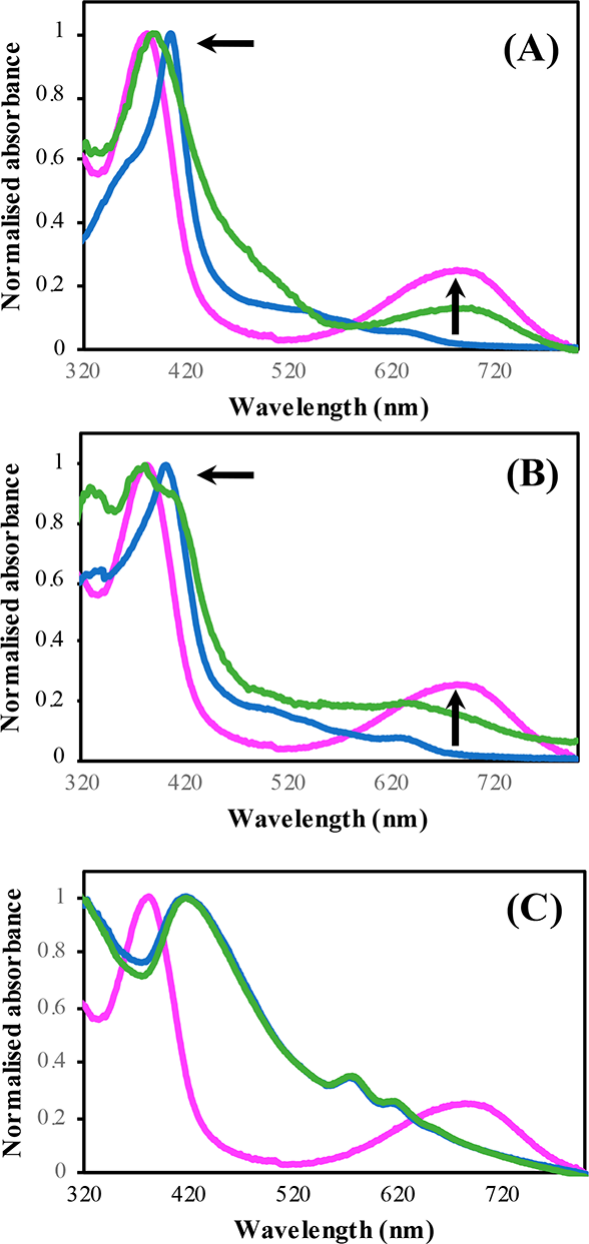
Normalized UV–vis spectra of (A) Fe–PPIX, (B) **Fe–L**^**1**^, and (C) **Fe–L**^**2**^ in *E. coli* lysates with (green) and without 16 h incubation with NADPH (blue). Biliverdin is highlighted in pink. Concentration = 50 μM, NADPH = 1 mM, 310 K.

In order to confirm the validity of the *E. coli* lysate system, Fe–PPIX was incubated with the lysate and NADPH (1 mM). Significant changes in the UV–vis absorbance spectrum indicative of heme degradation were observed following 16 h incubation after the addition of NADPH (Figure 4A). A hypsochromic shift of the Soret band from 405 to 388 nm and the appearance of a broad peak centered at 685 nm demonstrated effectively that heme catabolism to biliverdin was taking place. Similarly, when **Fe–L**^**1**^ was incubated with the *E. coli* lysate expressing hHO-1 and NADPH (Figure 4B), a partial 17 nm hypsochromic shift of the Soret band to 384 nm was displayed. The appearance of broad peaks at ca. 650 and 680 nm was also seen, confirming that **Fe–L**^**1**^ acts as a substrate for hHO-1. However, from the UV–vis spectra it is evident that the porphyrin degradation of **Fe–L**^**1**^ is not as efficient as Fe–PPIX, due to a split signal and the presence of the Soret band of **Fe–L**^**1**^ at 401 nm.

Absorbance studies of the dimethyl esters of Fe–PPIX (Fe–PPIX–DME) and **Fe–L**^**1**^ (**Fe–L**^**1**^**–DME**) in the *E. coli* lysate support literature precedent suggesting that the propionic acid residues are essential to maintain HO-1 activity.^40,66,67^ In both cases, very little spectral change was observed following incubation in the *E. coli* lysates with NADPH (Figure S13).

In contrast to Fe–PPIX and **Fe–L**^**1**^, minimal spectral differences were reported when **Fe–L**^**2**^ was incubated with hHO-1 and NADPH (Figure 4C). No shift in the porphyrin Soret band was observed, and no additional peak was formed at 680 nm, suggesting that HO-1 has little-to-no activity toward **Fe–L**^**2**^. Due to the modified porphyrin structure, biliverdin (or a biliverdin analogue) would not form during porphyrin catabolism of **Fe–L**^**2**^. However, if any activity toward HO-1 was displayed, the appearance of a peak in the range of 650–700 nm corresponding to the formation of an open-chain tetrapyrrole would be expected.

#### Fluorescence Spectroscopy

**Figure 5:**
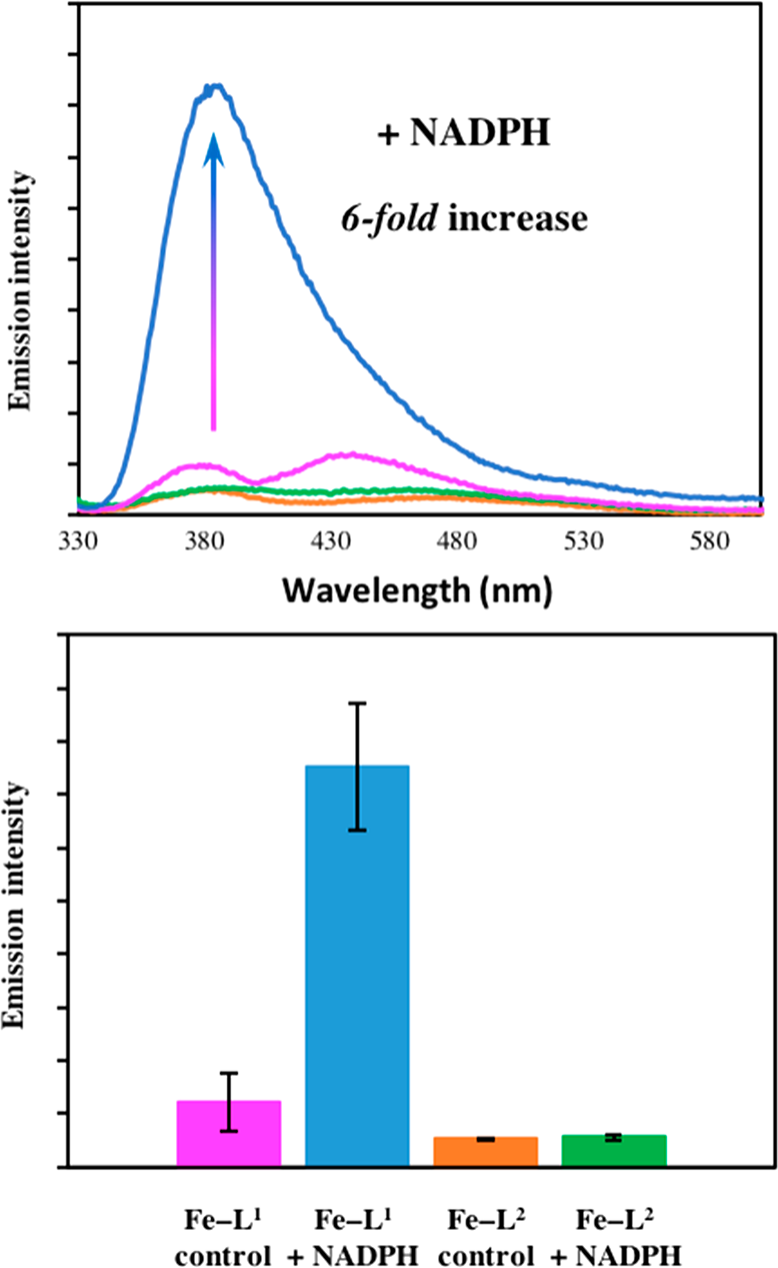
(A) Coumarin emission intensity of **Fe–L**^**1**^ and **Fe–L**^**2**^ in *E. coli* lysates with or without 16 h incubation with 1 mM NADPH. (B) Bar chart showing the average change in fluorescence intensity. Error bars represent the standard deviations of five independent experiments. **Fe–L**^**1**^ control (pink), **Fe–L**^**1**^ + NADPH (blue), **Fe–L**^**2**^ control (orange), and **Fe–L**^**2**^ + NADPH (green). Concentration = 50 μM, λ_*ex*_ = 320 nm, 298 K, slits 5:5.

The emission spectra of **Fe–L**^**1**^ and **Fe–L**^**2**^ in the hHO-1 *E. coli* lysate with and without incubation with NADPH (1 mM) are shown in Figure 5. In the absence of NADPH and incubation, very weak coumarin emission centered at 383 nm was observed for both coumarin–porphyrin diads. A broad peak at 440 nm accompanied the characteristic coumarin emission and was also present in the emission spectra of the *E. coli* lysate controls, likely due to NADPH. In the bacterial lysate, the coumarin emission intensity was lower in **Fe–L**^**2**^ compared to **Fe–L**^**1**^. Such a fluorescence response was also observed in PBS buffer, where a 3.0-fold decrease in the coumarin emission was reported for **Fe–L**^**2**^ vs **Fe–L**^**1**^ (Figure S14).

Fe-DMD analogue **Fe–L**^**1**^ displayed a *6-fold* increase in fluorescence at 383 nm following incubation with added NADPH versus the control (Figure 5). Such a wavelength maxima is closely aligned to that of the anticipated break-apart product, coumarin **1** (Figure S15). The significant *6-fold* “turn-on” in the fluorescence intensity in the coumarin region of the spectrum confirms that α-cleavage of the porphyrin is taking place, accompanied by the perturbation of the coumarin–porphyrin FRET mechanism. A fluorescence increase following incubation with NADPH supports the UV–vis data in Figure 4, where the formation of biliverdin was observed. Competitive activity studies with Zn-PP, a well-known HO-1 inhibitor, further confirmed that the increase in fluorescence is due to HO-1 catalyzed porphyrin degradation. Following addition of Zn-PP (50 μM) to the **Fe–L**^**1**^ reaction incubated with hHO-1 expressing *E. coli* lysate and NADPH, no increase in fluorescence emission intensity versus the control experiment was observed (Figure S16).

In contrast to **Fe–L**^**1**^, very little fluorescence enhancement was observed for **Fe–L**^**2**^ following the addition of NADPH to the *E. coli* lysates and 16 h incubation. Due to the enhanced fluorescence quenching of the coumarin moiety observed for **Fe–L**^**2**^ in the control samples, any substantial hHO-1 activity would be expected to cause a greater fluorescence enhancement than that of **Fe–L**^**1**^ as the break-apart product is formed.

The excitation spectra of **Fe–L**^**1**^ and **Fe–L**^**2**^ in the *E. coli* lysates are shown in Figure S17. Similar to the emission spectra, an increase in the excitation intensity was reported for **Fe–L**^**1**^ following incubation in the presence of excess NADPH. A *7-fold* increase at 320 nm was observed. A negligible response was seen for **Fe–L**^**2**^, further demonstrating that it is not metabolized by HO-1.

#### Mass Spectrometry Analysis

In order to further certify and understand the porphyrin catabolism of **Fe–L**^**1**^, mass spectrometry analysis of the *E. coli* lysates was undertaken (MALDI-MS and LC–MS). It was also envisaged that these techniques would provide additional information on the regioselectivity of the α-cleavage and confirm the chemical structure of the break-apart products.

Prior to MS analysis, reaction samples from the *E. coli* lysates were first acidified in H_2_SO_4_/methanol (5% v/v H_2_SO_4_) to form their subsequent methyl esters and extracted into chloroform. Such procedures have been used previously to good effect to form organic soluble biliverdin dimethyl esters, enabling the regioselectivity of heme analogues to be studied following porphyrin catabolism^43,44^

From MALDI-MS it was determined that porphyrin catabolism is regioselective at the α-position with the release of the coumarin donor fluorophore. Following incubation of **Fe–L**^**1**^ in *E. coli* lysates containing excess NADPH, there was only evidence for the formation of one biliverdin compound with a mass of 587 Da (Figure S18). Signals for both mono- and dimethyl esters of **Fe–L**^**1**^ are also present, supporting the UV–vis data (Figure 4) in suggesting that porphyrin catabolism is not 100% efficient. Analysis of the control assay without incubation with NADPH showed no evidence of α-cleavage (Figure S19).

In contrast to **Fe–L**^**1**^, MALDI-MS of **Fe–L**^**2**^ reaction mixture extracts in the absence and presence of NADPH produced identical MALDI-MS spectra (Figures S20 and S21). There was no evidence toward formation of any porphyrin degradation products following incubation with excess NADPH.

**Figure 6:**
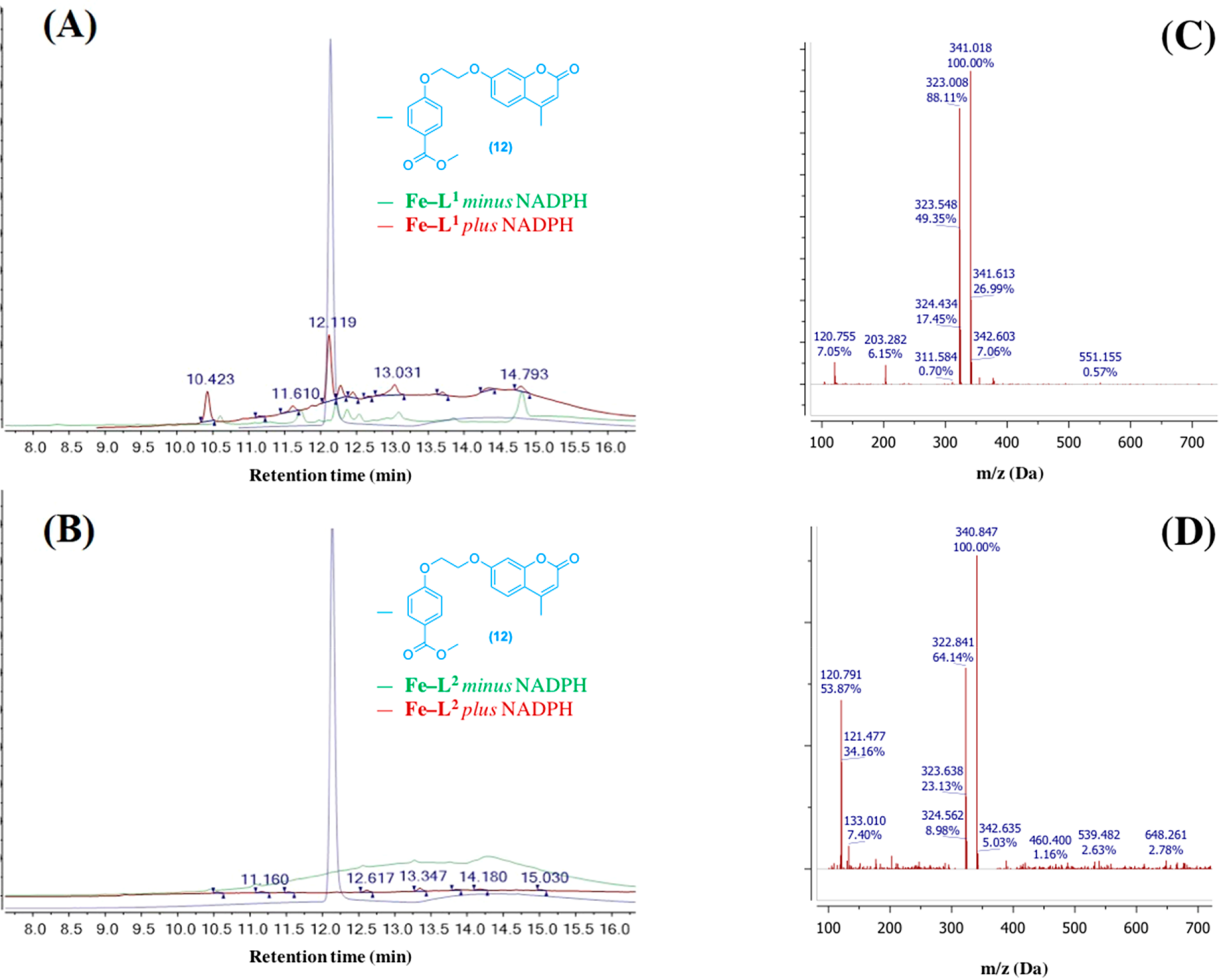
LC chromatograms of acidified reaction mixture extracts for (A) **Fe–L**^**1**^ and (B) **Fe–L**^**2**^ with synthesized methyl ester analogue **12** (blue) as a comparison. **Fe–L**^***n***^ + NADPH (red), **Fe–L**^***n***^ control (green). UV detection at 320 nm (λ_max_ of **12**). Concentration of **Fe–L**^**1**^ and **Fe–L**^**2**^ = 200 μM. (C) (+) *m*/*z* of **12** at a retention time of 12.1 min and (D) (+) *m*/*z* of **Fe–L**^**1**^ following addition of NADPH at a retention time of 12.1 min. During LC–MS, methyl ester hydrolysis of **12** to **1** was observed.

In order to confirm the identity of break-apart coumarin byproduct following HO-1 catabolism, the acidified reaction extracts were analyzed by liquid chromatography mass spectrometry (LC–MS). Samples of **Fe–L**^**1**^ and **Fe–L**^**2**^ with and without incubation with NADPH (1 mM) were compared to compound **12**, the methyl ester of the predicted break-apart product **1**. The UV detection was set to 320 nm, the absorbance wavelength maximum for **12** and **1** (Figure 6).

Compound **12** displayed a LC retention time of 12.1 min and a mass of 341 Da, suggesting that methyl ester hydrolysis was occurring during the analysis (Figure 6A,C). It was found that incubating **Fe–L**^**1**^ in the *E. coli* lysate in the presence of NADPH produced an identical species with the same LC retention time and mass (Figure 6A,D). Such a result confirms the formation of compound **1** following porphyrin catabolism, and its formation is responsible for the *6-fold* increase in fluorescence at 383 nm. The formation of compound **1** and the presence of **12**, was not detected in the LC trace of the control reaction of **Fe–L**^**1**^ (minus NADPH), or for the reaction mixture extracts of **Fe–L**^**2**^. Such an observation supports the UV–vis and fluorescence measurements in Figure 4 and 5 respectively, strongly suggesting that an analogue of Fe–PPIX, or in this case, Fe–DMD is required in order to maintain hHO-1 activity.

## Conclusions

Herein, we report the design, synthesis, photophysical, and biological characterization of **Fe–L**^**1**^, a coumarin-porphyrin FRET probe for hHO-1. We have shown using an *E. coli* lysate overexpressing hHO-1 that following incubation with excess NADPH a *6-fold* “turn-on” in fluorescence at 383 nm is observed. The increase in fluorescence is due to porphyrin α-cleavage and the perturbation of FRET from a coumarin donor to a porphyrin acceptor fluorophore. The identity of the coumarin break-apart product was confirmed by LC-MS to be compound **1**, supporting work by Ortiz de Montellano and co-workers who detected the formation of benzoic acid following HO-1 oxidation of α-*meso*-phenylheme.^43^ Moreover, we have demonstrated from MALDI-MS analysis that porphyrin catabolism of **Fe–L**^**1**^ is regiospecific at the α-position.

HP-TPP analogue **Fe–L**^**2**^ was found to not act as a substrate for hHO-1 from UV–vis, fluorescence, and mass spectrometry analysis. Therefore, it was used as a control in the assay experiments. The synthesis and analysis of **Fe–L**^**2**^ have provided an additional insight into the structural tolerance of the hHO-1 active site. It is likely that the addition of three *meso*-phenyl substituents provide an additional steric hindrance in the HO-1 binding pocket reducing its binding affinity and subsequent activity. It is expected that the absence of two propionic acid residues in **Fe–L**^**2**^ is also responsible for the lack of metabolism by HO-1. These residues are known to have important electrostatic interactions with positively charged side chains lysine (Lys18) and arginine (Arg183), anchoring heme analogues in the HO-1 active site. The importance of the propionic acid residues is further highlighted from the UV–vis spectra of Fe–PPIX–DME and **Fe–L**^**1**^**–DME** in *E. coli* lysates where no spectral change was reported following addition of NADPH and incubation.

To the best of our knowledge, this is the first known chemical probe capable of detecting HO-1 activity. The work detailed here shows very encouraging and promising results. Following our proof-of-concept study in *E. coli* lysates, we are now investigating the biological characterization of **Fe–L**^**1**^ in mammalian cells and human derived macrophages, with promising data (Figure S22). Moreover, it is thought that a FRET break-apart probe of this nature could be used to identify HO-1 overexpression in real-time and as a research tool to accelerate work on the pathophysiology of HO-1. Critically, it could be developed into a real-time imaging and detection reagent that would allow the detection of hemorrhagic pathologies. These include coronary intraplaque hemorrhage in cardiology, or other internal hemorrhages in stroke or aneurysm. Since hemorrhage or HO-1 is suspected to play a role in many pathologies, the scope for development is significant. We also note that this is one of the first descriptions of the application of the FRET break-apart principle to a small/medium-sized molecule metabolite. This principle could, therefore, be extended to other interesting medium-sized metabolites, such as lipids.

The structure of our probe offers flexibility in design for future modifications, with the possibility of developing a FRET system containing a more favorable NIR fluorophore. First, a NIR analogue could be achieved following coordination of an alternative metal ion, e.g. Mg^2+^, allowing the porphyrin to act as a fluorescent donor to a NIR acceptor. Another possible avenue to explore is to incorporate both the donor and acceptor fluorophores onto the structure of Fe-DMD. We are also exploring the possibility of experimenting with altering the polypyrrole structure to red-shifted polypyrroles, such as bacteriochlorins, that are expected to interact with some of the smaller NIR dyes. To this end, work is currently ongoing to develop new probes with red-shifted wavelength maxima, to improve the photophysical properties for live fluorescence imaging. It is important to note that care must also be taken to ensure that the chosen fluorophore(s) have a high photostability prior to undertaking these experiments.

The development of such probes will continue to further advance the field and could enable a range of cardiovascular and neurodegenerative disorders to be diagnosed more effectively in the near future.

## Supporting Information Available

Additional figures, synthetic and experimental procedures (PDF)
