# Supplementary material for: A Coumarin–Porphyrin FRET Break-Apart Probe for Heme Oxygenase-1
Source: J Am Chem Soc. 2021 Apr 13;143(17):6460–9. doi: 10.1021/jacs.0c12864 (PMC8154531; doi:10.1021/jacs.0c12864)
Supplement: Supplementary file 1 — ja0c12864_si_001.pdf [file ja0c12864_si_001.pdf]

# Supporting Information for:

# A coumarin-porphyrin FRET break-apart probe for Heme Oxygenase-1 (HO-1).

Edward R. H. Walter,[†, ‡] Ying Ge,[‡] Justin C. Mason,[‡] Joseph J. Boyle[‡*] and Nicholas J. Long. [†*]

[†] Department of Chemistry, Imperial College London, Molecular Sciences Research Hub, White City Campus, Wood Lane, London, W12 0BZ, UK

[‡] Vascular Sciences, National Lung and Heart Institute, Imperial College London, Du Cane Road, London, W12 0NN, UK.

*Corresponding authors: Joseph J. Boyle (joseph.boyle@imperial.ac.uk)

Nicholas J. Long (n.long@imperial.ac.uk)

**Table of Contents**

*1. Additional Figures and Schemes* ........ - 3 -

*2. Experimental methods* ........ - 14 -

2.1 Photophysical Characterisation ........ - 14 -

2.2 Biological evaluation ........ - 16 -

2.3 Compound synthesis ........ - 19 -

*3. Compound Characterisation* ........ - 27 -

3.1 $^{1}H$ and $^{13}C$ NMR spectra ........ - 27 -

3.2 Mass Spectrometry data ........ - 35 -

*4. References* ........ - 44 -

## 1. Additional Figures and Schemes

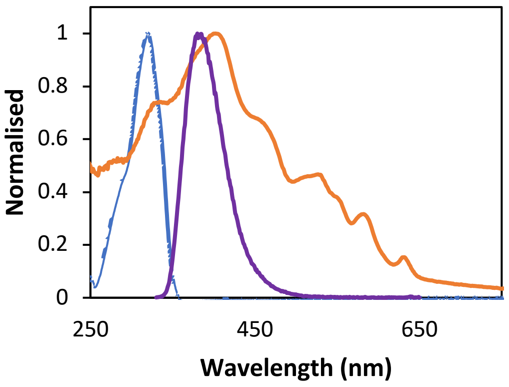


**Figure S1.** The spectral overlap of the emission spectrum of 7-hydroxymethyl coumarin analogue (*purple,* $\lambda_{ex}$= 320 nm), **(7)** with the absorbance spectrum of porphyrin **(6)** (*orange*). Absorbance of **(7)** is shown in *blue*. Concentration = 20 $\mu$M in PBS buffer (pH = 7.4), 298 K.

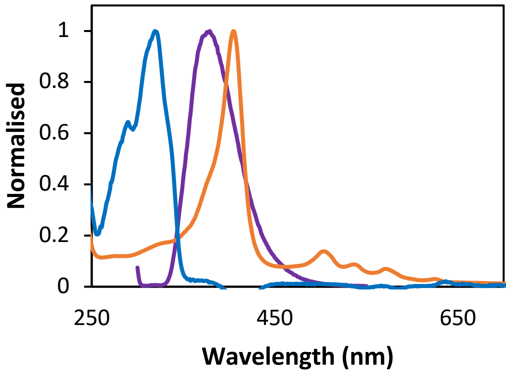


**Figure S2.** The spectral overlap of the emission spectrum of 7-hydroxymethyl coumarin analogue (*purple,* $\lambda_{ex}$= 320 nm), **(7)** with the absorbance spectrum of porphyrin **(6)** (*orange*). Absorbance of **(7)** is shown in *blue*. Concentration = 10 $\mu$M in $CHCl_3$ (pH = 7.4), 298 K.

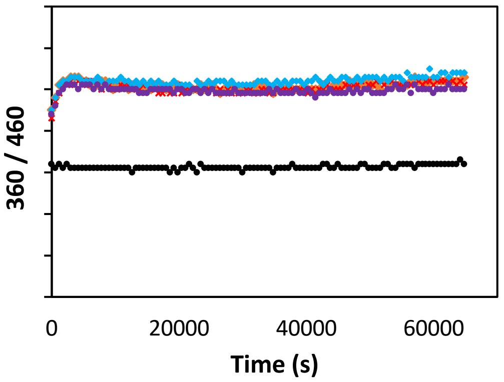


**Figure S3.** Fluorescence emission of **(1)** in potassium phosphate buffer (*blue*), Tris buffer (*red*), MOPS (*purple*) at pH 7.4 and $H_2O$ (*orange*) ($\lambda_{ex}$= 360 nm, $\lambda_{em}$= 460 nm). Measurements were recorded every 10 min for 18 h. A blank (*black*) was run in $H_2O$ without addition of compound **(1)**.

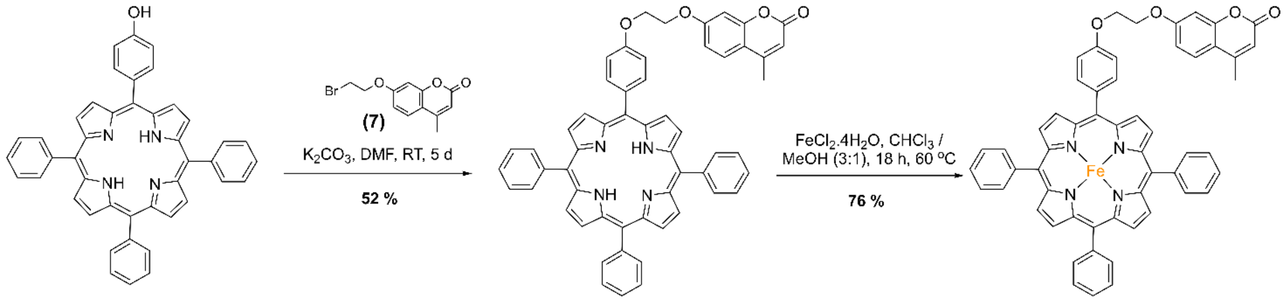


**Scheme S1.** The synthesis of **Fe–L$^2$**.

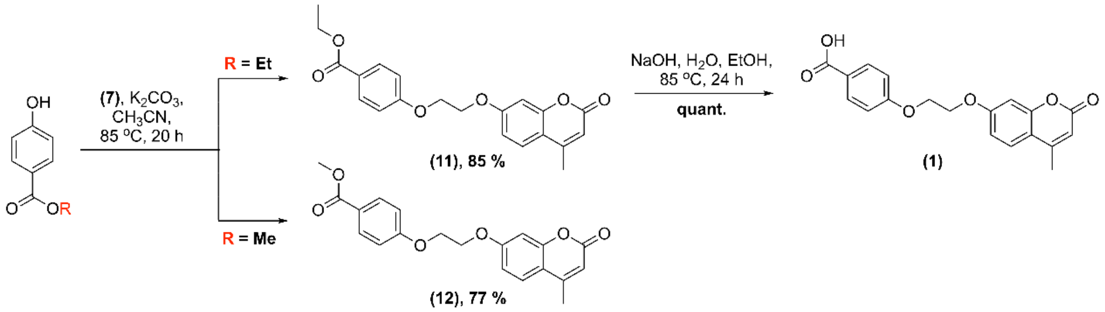


**Scheme S2.** The synthesis of coumarin derivatives **(11)**, **(12)** and **(1)**.

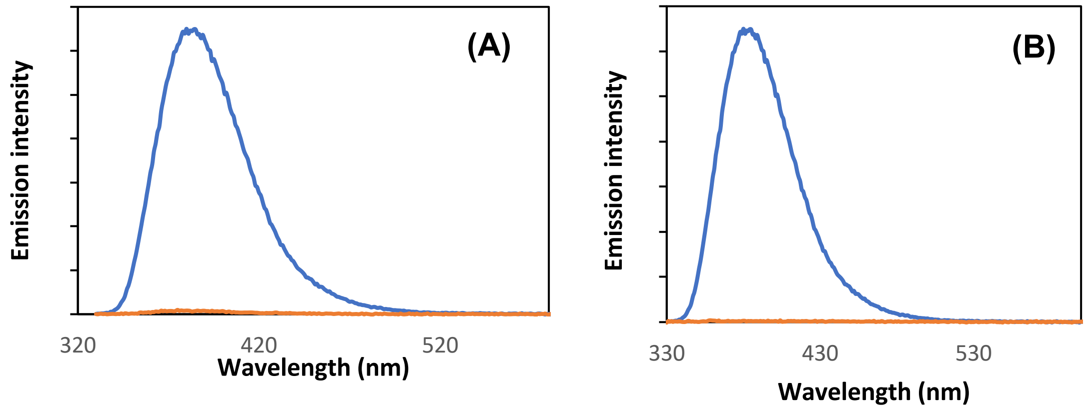


**Figure S4.** Highlighting the coumarin emission and FRET efficiency of **(A) (7)** (*blue*) and **Fe–L$^1$** (*orange*) and **(B) (7)** (*blue*) and **Fe–L$^2$** (*orange*). Concentration = 20 $\mu$M in PBS buffer (pH = 7.4), 298 K, $\lambda_{ex}$= 320 nm slits 2.5:2.5.

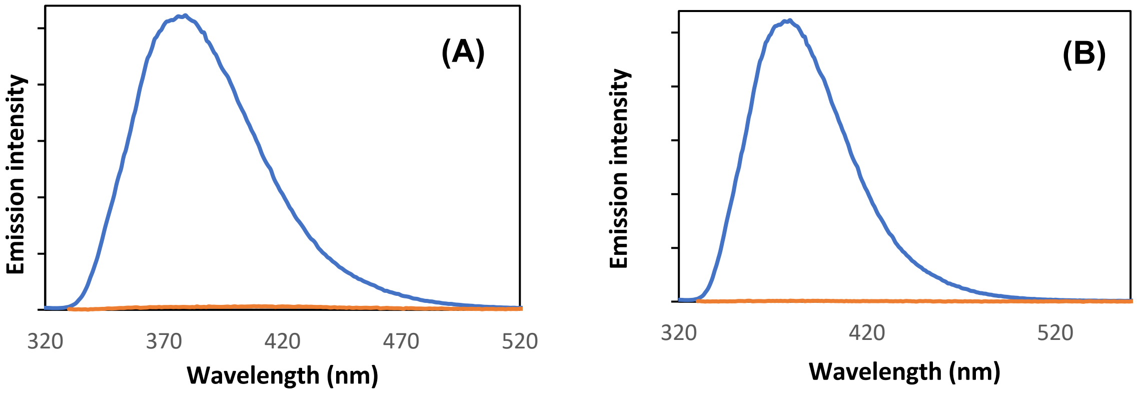


**Figure S5.** Highlighting the coumarin emission and FRET efficiency of **(A) (7)** (*blue*) and **Fe–L[1]** (*orange*) and **(B) (7)** (*blue*) and **Fe–L[2]** (*orange*). Concentration = 10 $\mu$M in $CHCl_3$, 298 K, slits $\lambda_{ex}$= 320 nm, 5:5.

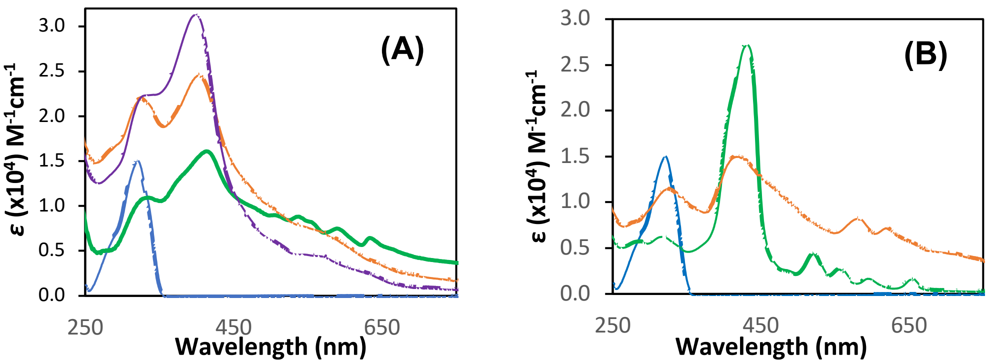


**Figure S6.** The absorbance spectra of **(A) (7)** (*blue*), **L[1]–DME** (*green*), **Fe-L[1]–DME** (*orange*) and **Fe–L[1]** (*purple*) and **(B) (7)** (*blue*), **L[2]** (*green*) and **Fe–L[2]** (*orange*). Concentration = 20 $\mu$M in PBS buffer pH = 7.4, 298 K.

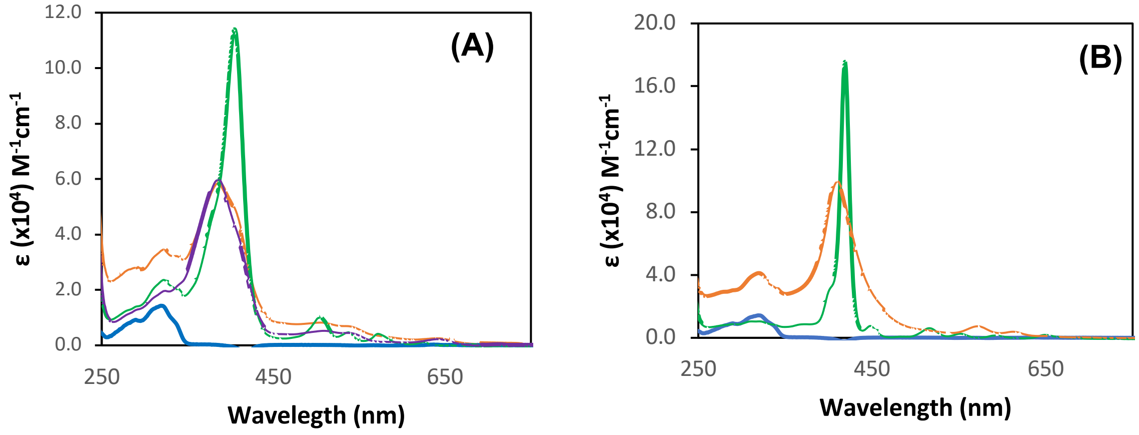


**Figure S7.** The absorbance spectra of **(A) (7)** (*blue*), **L[1]–DME** (*green*) and **Fe–L[1]–DME** (orange) and **Fe–L[1]** (*purple*) and **(B) (7)** (*blue*), **L[2]** (*green*) and **Fe–L[2]** (*orange*). Concentration = 10 $\mu$M in $CHCl_3$ 298 K.

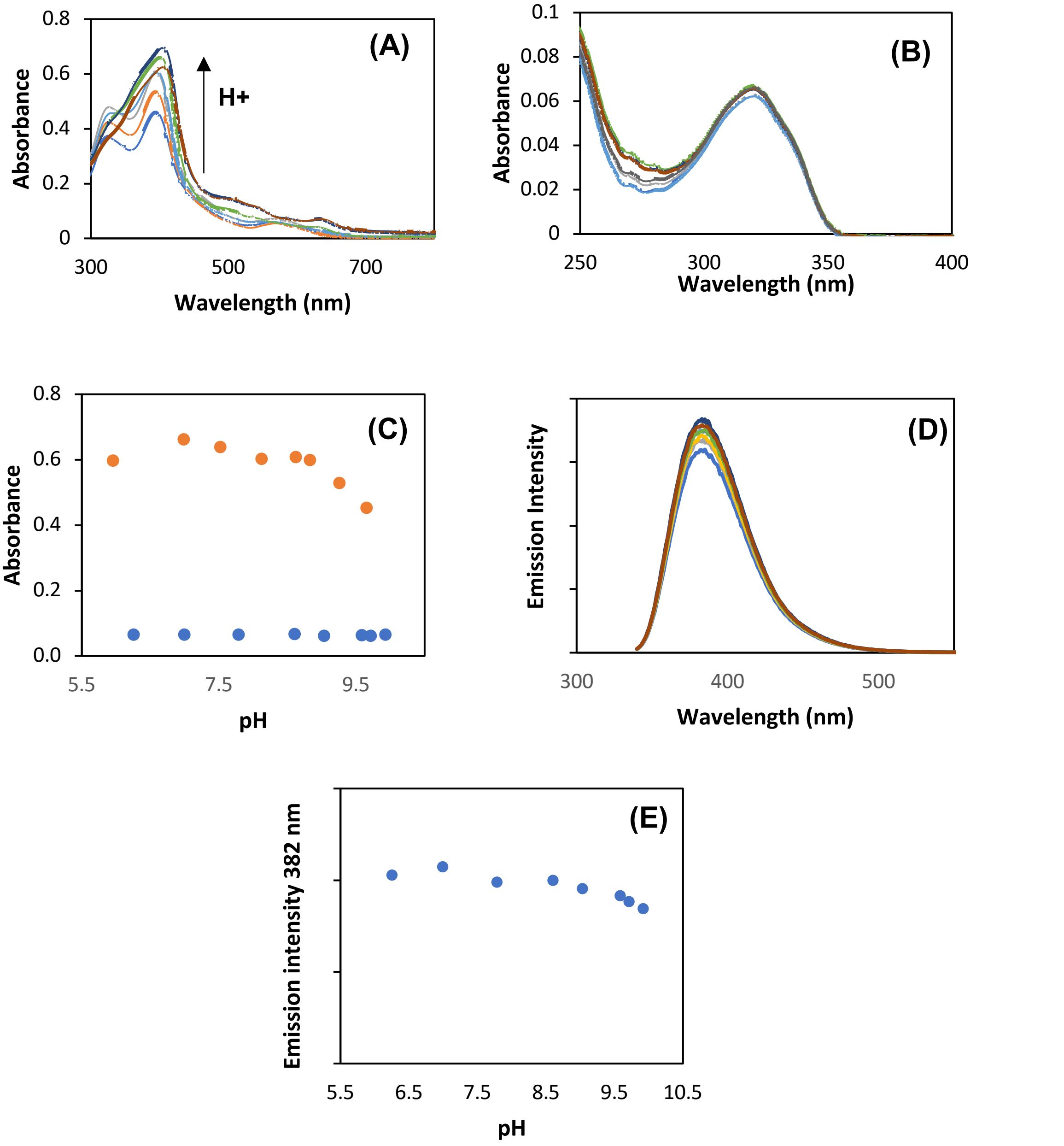


**Figure S8.** Variation of the absorbance spectra with pH for **(A) Fe–L**[1] and **(B)** coumarin **(1)**. **(C)** Plot of absorbance against pH for **Fe–L**[1] ($\lambda_{abs}$= 400 nm, *orange*) and **(1)** ($\lambda_{abs}$= 320 nm, *blue*) **(D)** Variation of the emission spectra of **(1)** with pH and **(E)** Plot of emission against pH for **(1)** ($\lambda_{em}$= 382 nm), slits 5:5, $\lambda_{ex}$= 320 nm. Concentration = 20 $\mu$M in Tris (25 mM) and NaCl (150 mM), 298 K.

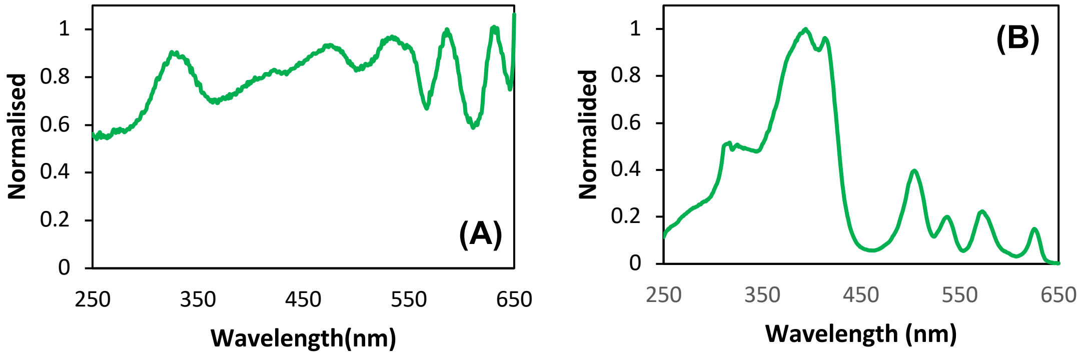


**Figure S9.** The excitation spectra of **L**[1]**–DME** in **(A)** PBS buffer (pH = 7.4) ($\lambda_{em}$ = 674 nm, slits 10:10, 435 nm filter) and **(B)** $CHCl_3$ ($\lambda_{em}$ = 627 nm, slits 5:5) at 298K.

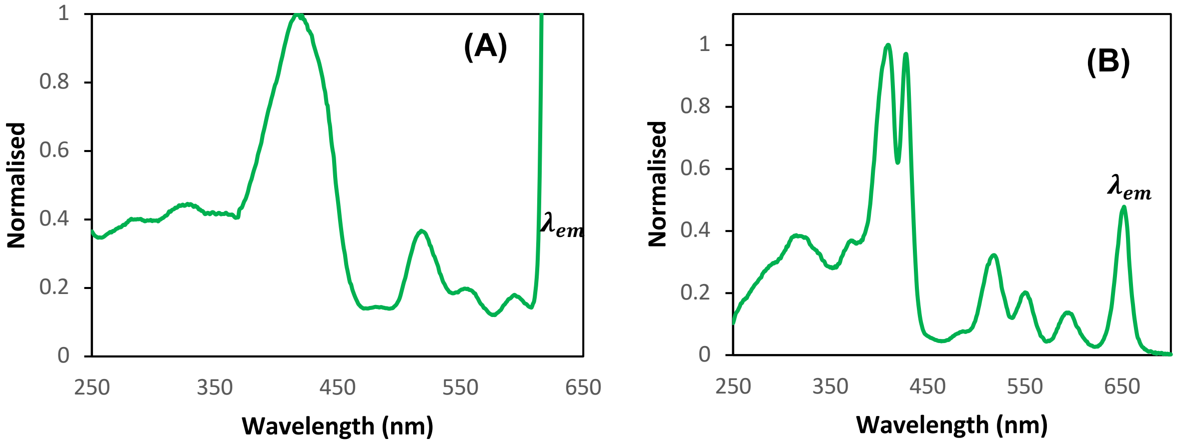


**Figure S10.** The excitation spectra of **L**[2] in **(A)** PBS buffer (pH = 7.4) ($\lambda_{em}$ = 639 nm, slits 10:10) and **(B)** $CHCl_3$ ($\lambda_{em}$ = 654 nm, slits 5:5) at 298K.

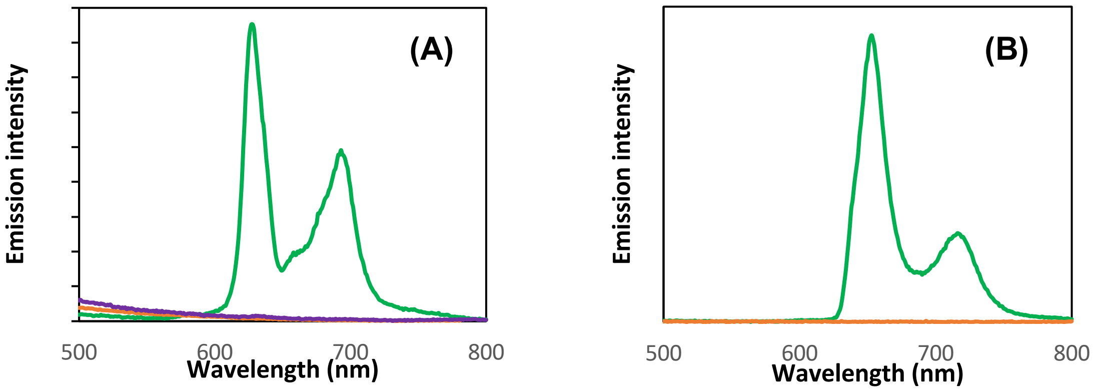


**Figure S11.** Porphyrin emission spectra of **(A) L**[1]**–DME** (*green*)**, Fe–L**[1]**–DME** (*orange*), and **Fe–L**[1] (*purple*) and **(B) L**[2] (*green*) and **Fe–L**[2] (*orange*). Concentration = 10 $\mu$M in $CHCl_3$ 298 K, $\lambda_{ex}$ = 320 nm, slits 5:5.

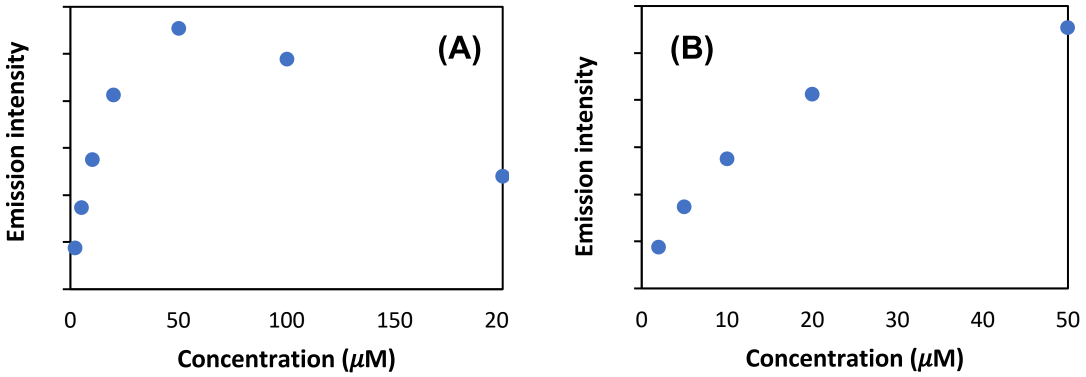


**Figure S12.** Variation of the coumarin emission intensity ($\lambda_{em}$ = 383 nm) of **(1)** in PBS buffer (pH = 7.4). **(A)** Emission intensity from 2 $\mu$M to 200 $\mu$M and **(B)** focusing on 2 $\mu$M to 50 $\mu$M. $\lambda_{ex}$ = 320 nm, 298 K.

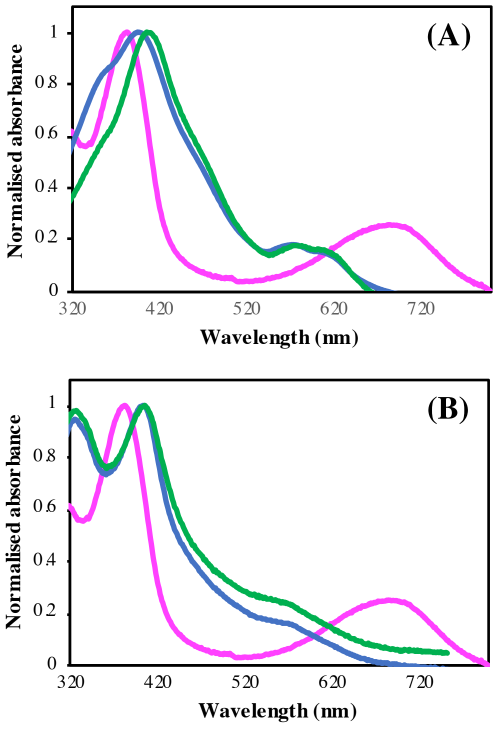


**Figure S13.** Normalised UV-Vis spectra of **(A)** Fe–PPIX–DME and **(B) Fe–L$^1$–DME** in *E. coli* lysates with (*green*) and without incubation with NADPH (*blue*). Biliverdin is highlighted in *pink*. Concentration of porphyrin = 50 $\mu$M, NADPH = 1 mM, 310 K.

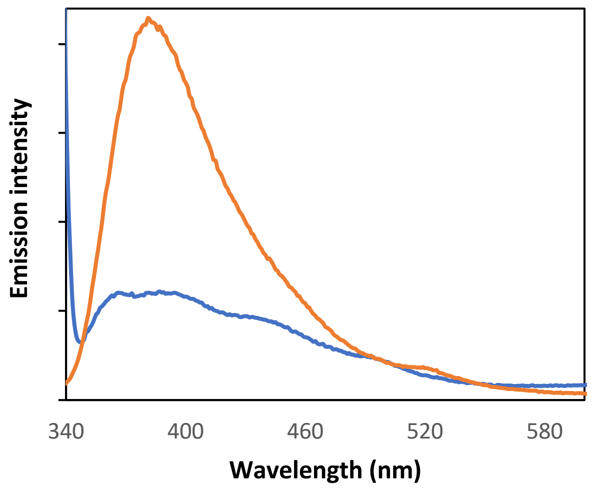


**Figure S14.** Coumarin emission of **Fe–L**[1] (*orange*) and **Fe–L**[2] (*blue*) in PBS buffer (pH = 7.4). Concentration = 20 $\mu$M, $\lambda_{ex}$ = 320 nm, 298 K, slits 10:10.

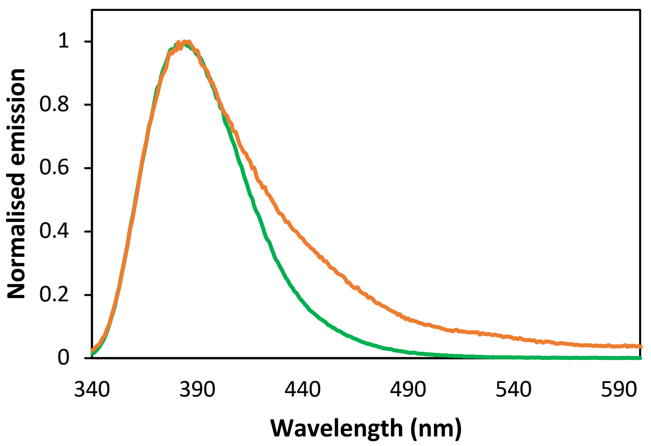


**Figure S15.** Normalised coumarin emission spectra of **Fe–L**[1] in *E. coli* lysate with incubation with NADPH (*orange)* and coumarin **(1)** (*green*) in PBS buffer (pH = 7.4), $\lambda_{ex}$= 320 nm, 298 K.

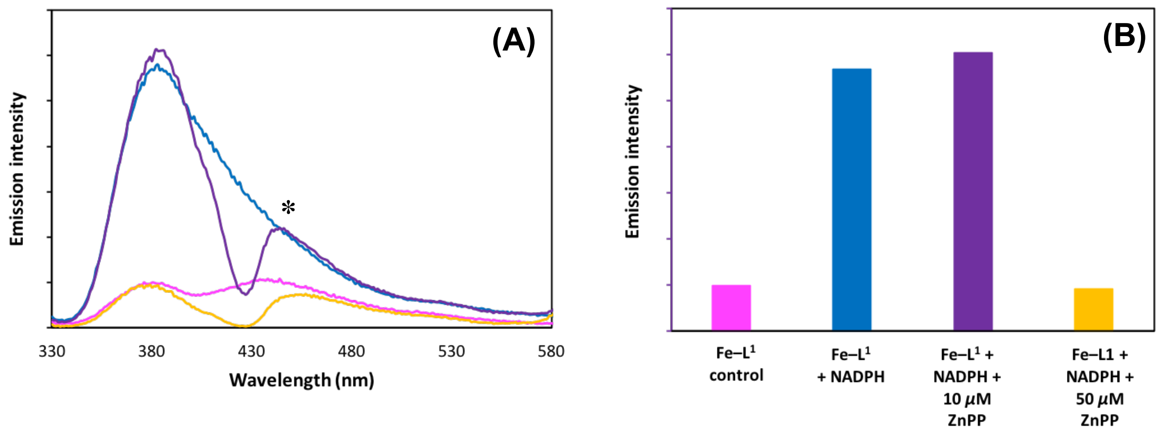


**Figure S16. (A)** Coumarin emission intensity of **Fe–L$^1$** in *E. coli* lysates with or without 16 h incubation with 1 mM NADPH and inhibition studies with ZnPP. **(B)** Bar chart showing the change in fluorescence intensity. **Fe–L$^1$** control (*pink*), **Fe–L$^1$**+NADPH (*blue*), **Fe–L$^1$**+NADPH+10 $\mu$M ZnPP (*purple*) and **Fe–L$^1$**+ NADPH+50 $\mu$M ZnPP (*yellow*). * Peak at 450 nm present in the lysate (no porphyrin) control. **Fe–L$^1$** concentration = 50 $\mu$M, $\lambda_{ex}$= 320 nm, 298 K, slits 5:5.

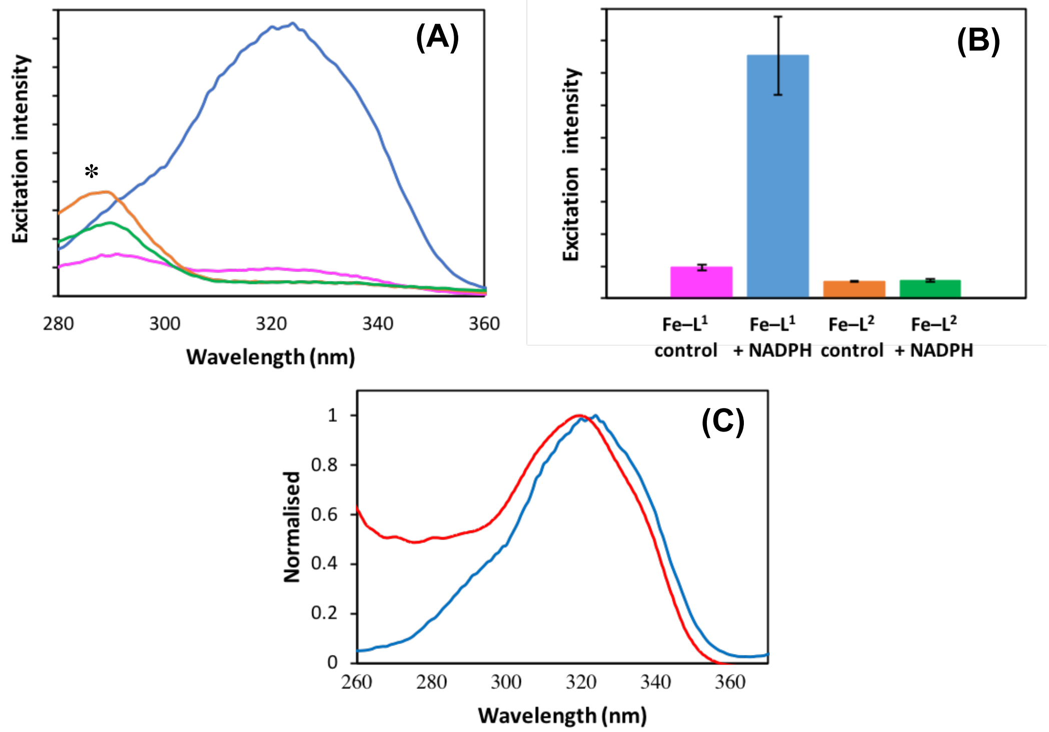


**Figure S17. (A)** Coumarin excitation spectra following incubation of **Fe–L$^1$** and **Fe–L$^2$** in *E. coli* lysates with 1mM NADPH, or in unincubated (no NADPH) controls. * peak at 290 nm present in the lysate (no porphyrin) control. **(B)** Bar chart showing the average change in excitation intensity. Error bars represent the standard deviation of five independent experiments. **Fe–L$^1$** control (*pink*), **Fe–L$^1$**+NADPH (*blue*), **Fe-L$^2$** control (*orange*) and **Fe–L$^2$**+NADPH (*green*). Concentration = 50 $\mu$M, $\lambda_{ex}$= 320 nm, 298 K, slits 5:5. **(C)** Normalised excitation spectrum of **Fe–L$^1$**+NADPH (*blue*) and absorbance spectrum of coumarin **(1)** (*red*).

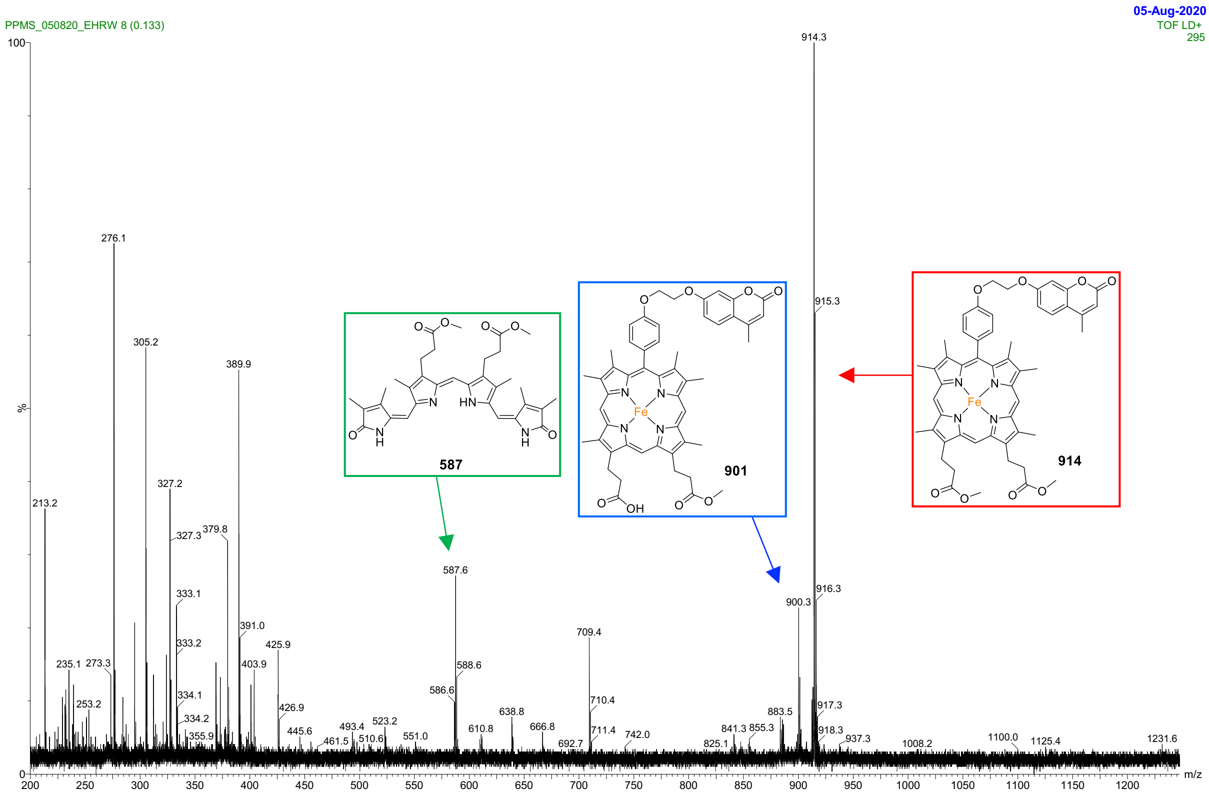


**Figure S18.** The MALDI-MS of **Fe–L**[1] following 16 h incubation with 1 mM NADPH and *E. coli* lysate and acidification and extraction into chloroform.

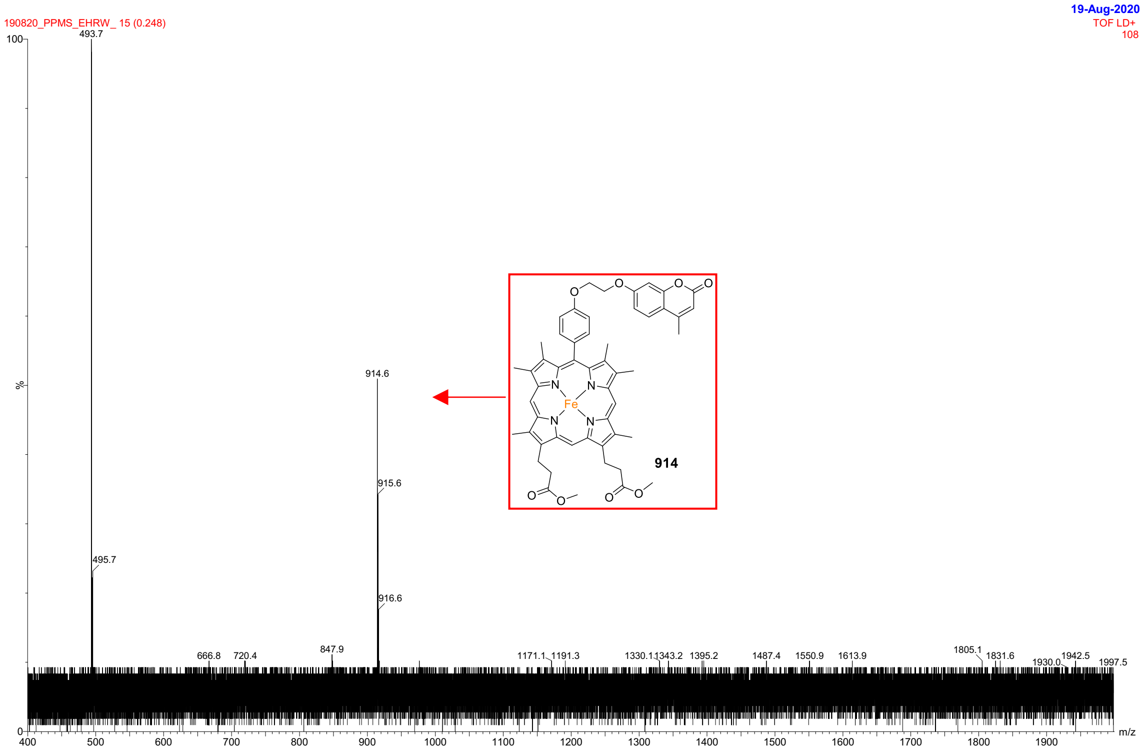


**Figure S19.** The MALDI-MS of **Fe–L**[1] minus NADPH (control) in *E. coli* lysates after acidification and extraction into chloroform.

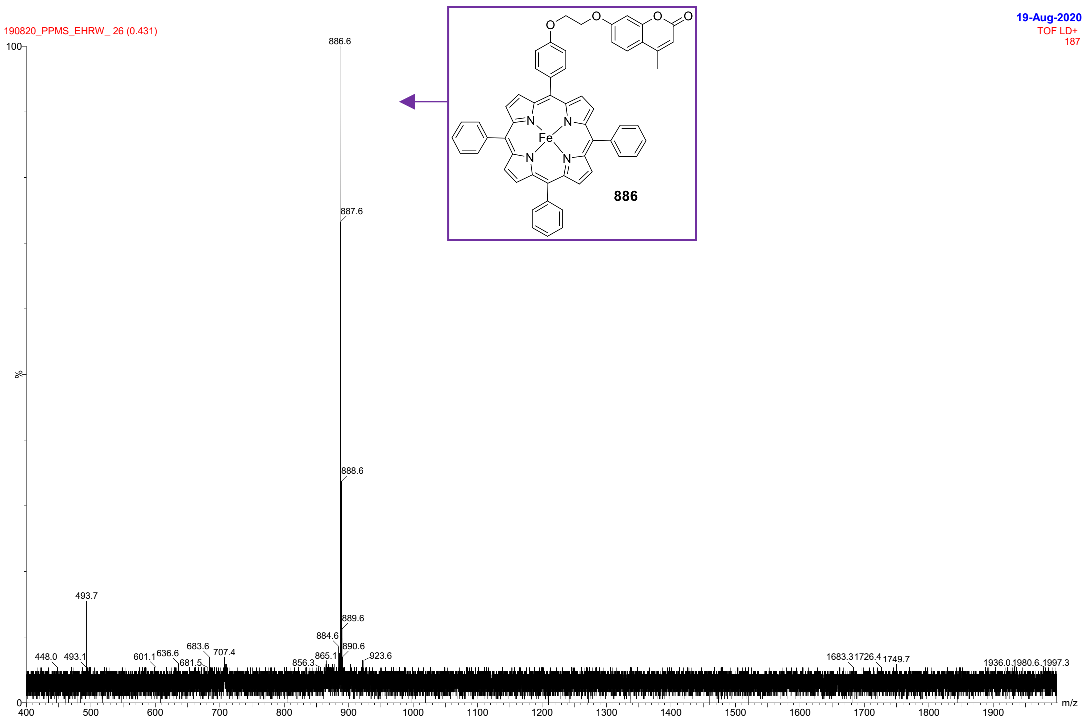


**Figure S20.** The MALDI-MS of **Fe–L$^2$** following incubation with 1 mM NADPH and *E. coli* lysates and acidification and extraction into chloroform.

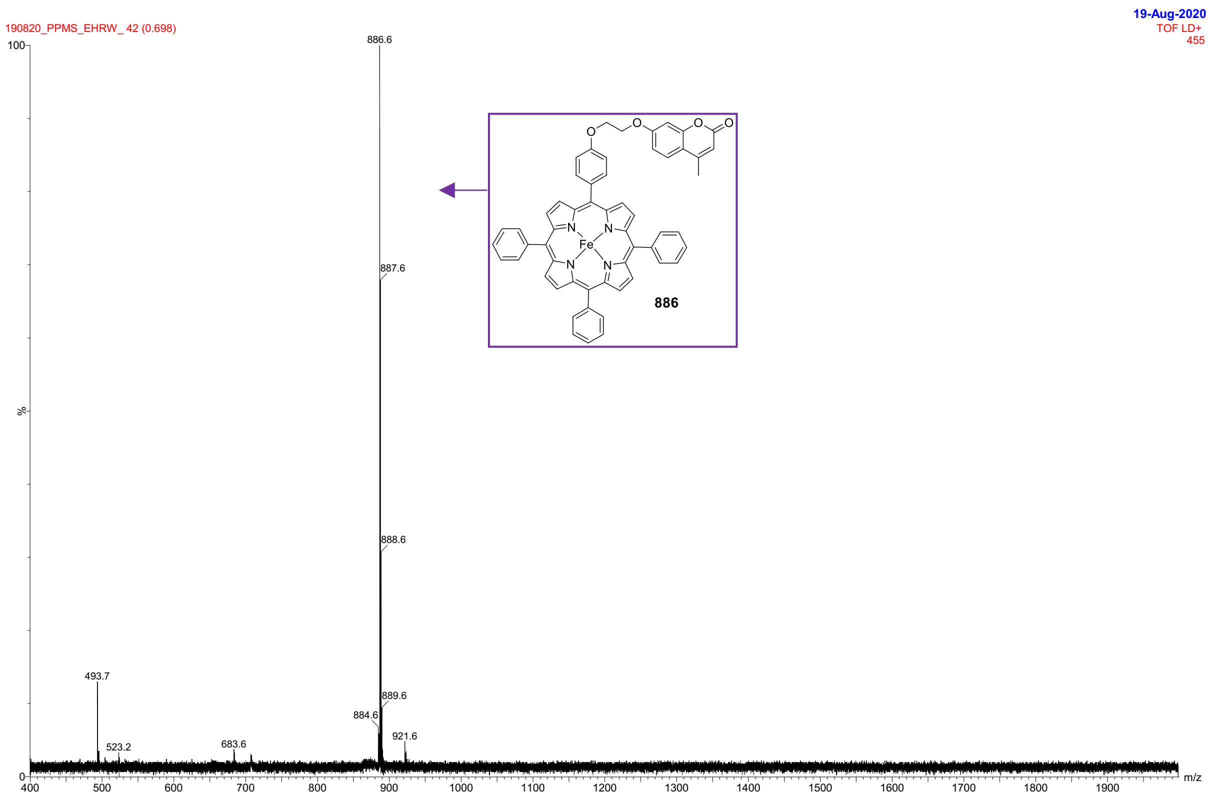


**Figure S21.** The MALDI-MS of **Fe–L$^2$** minus NADPH (control) in *E. coli* lysates after acidification and extraction into chloroform.

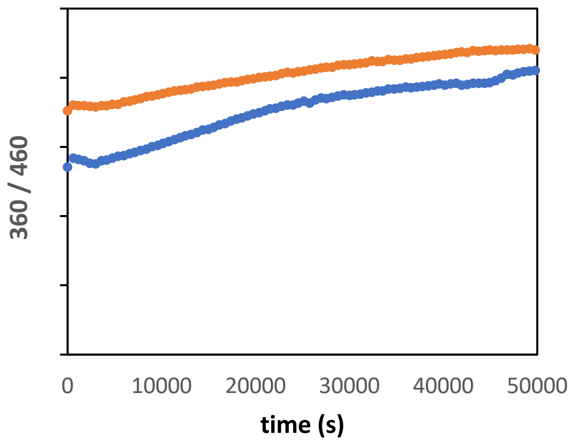


**Figure S22.** Increase in coumarin emission of **Fe–L[1]** in vehicle (*blue*) and heme treated (*orange*) human derived macrophages. Measurements were recorded every 10 min for 14 h. Concentration = 50 $\mu$M, $\lambda_{ex}$= 360 nm, $\lambda_{em}$= 460 nm in MOPS buffer, pH = 7.4, 298 K.

**Table S1.** Photophysical data for **(1)**, **(7)**, and coumarin-porphyrin diads discussed in this study. Concentration = 10 $\mu$M in $CHCl_3$, $\lambda_{ex}$ = 320 nm, 298 K. Quantum yields (∅) ± 20 % were measured using tetraphenylporphyrin ($H_2TPP$) in toluene ($\emptyset_{514\,nm}$ = 0.11) as the standard.[1]

| **Compound/ complex** | **$\lambda_{abs}$ [nm] ($\varepsilon$ / $10^4$ $M^{-1}cm^{-1}$)** | | | **$\lambda_{em}$ [nm]** | **E [%]** | **$\emptyset_{514\,nm}$ [%]** |
|---|---|---|---|---|---|---|
| | **UV** | **Soret** | **Q-band** | | | |
| **(1)** | 291 (0.9)<br>321 (1.7) | – | – | 381 | – | – [c] |
| **(7)** | 291 (0.9)<br>323 (1.4) | – | – | 382 | – | – [c] |
| **L[1]–DME** | 291 (1.4)<br>320 (2.4) | 405 (11.4) | 503 (1.0), 538 (0.5), 572 (0.4), 625 (0.2) | 385[a], 629, 695 | 97.7 | 5.2 |
| **Fe–L[1]–DME** | 291 (2.8)<br>321 (3.5) | 384 (5.9) | 506 (0.9), 535 (0.7), 638 (0.3) | 385[a], [b] | 98.7 | – [c] |
| **Fe–L[1]** | 320 (2.0) | 385 (6.0) | 523 (0.5), 548 (0.4), 640 (0.3) | 385 [a], [b] | 99.2 | – [c] |
| **L[2]** | 291 (0.9)<br>320 (1.1) | 419 (17.6) | 516 (0.7), 552 (0.3), 592 (0.2), 649 (0.3) | 379[a], 654, 719 | 96.6 | 17.4 |
| **Fe–L[2]** | 320 (4.1) | 409 (10.0) | 572 (0.8), 618 (0.4) | 385[a], [b] | 99.8 | – [c] |

[a] Residual coumarin emission, [b] no porphyrin emission was observed, [c] no quantum yield was measured.

## 2. Experimental methods

**General Procedures:** All commercially available reagents were used as received from suppliers without further purification. Solvents used were laboratory grade. Anhydrous solvents were obtained from departmental solvent towers and stored over 3 Å molecular sieves. Moisture-sensitive reactions were carried out by Schlenk-line techniques, under an inert atmosphere of nitrogen. Thin-layer and column chromatography was performed on silica (Merk Art 5554) and visualised under UV radiation. $^{1}H$ (400 MHz) and $^{13}C\{H\}$ (101 MHz) NMR spectra were recorded on a Bruker AV-400 spectrometer, Imperial College London at 298 K. Chemical shifts are reported in parts per million (ppm) and coupling constants in Hertz (Hz). Peak multiplicities are abbreviated as; s = singlet, m = multiple, d = doublet, t = triplet, q = quartet, dd = doublet of doublet and br = broad. Mass spectrometry analysis (ESI and MALDI-MS) was conducted by the Mass Spectrometry Service, Imperial College London, unless stated otherwise.

### 2.1 Photophysical Characterisation

**Sample preparation:** Stock solutions of **L$^1$**, **Fe–L$^1$**, **L$^2$** and **Fe–L$^2$** analogues were made in DMSO with a concentration range of 1 – 10 mM and stored at -20 °C in the dark. Samples were thawed to room temperature directly before use. All samples were diluted to 20 $\mu$M in PBS buffer pH = 7.4 (ThermoFisher, tissue culture grade, 10010023) and 10 $\mu$M in $CHCl_3$ buffer for the photophysical measurements. Total concentration of DMSO in samples run for photophysical measurements was < 1 %.

**Absorption spectroscopy:** UV-Visible absorption spectra were measured using an Agilent Technologies Cary 60 Spectrophotometer operating with WinUV software. The sample was held in a quartz cuvette with a path length of 1 cm. Absorption spectra were recorded against a baseline of pure solvent in a optically matched cuvette with a scan rate of 600 nm / min and

a data interval of 1.0 nm. Extinction coefficients were calculated from the Beer Lambert Law (**Equation 1**).

$$A = \varepsilon c l \qquad \textbf{Eq 1.}$$

Where A = the absorbance at a particular wavelength, $\varepsilon$ is the extinction coefficient, c is the concentration and l is the path length (width of the quartz cuvette, 1 cm).

Absorbance spectra to quantify the HO-1 activity were measured in transparent 96-well plates with a Beckman Coulter Paradigm plate reader. Fluorescent spectra were obtained in a quartz cuvette using a Cary Eclipse Fluorescence Spectrophotometer (Agilent).

**Fluorescence spectroscopy:** Emission and excitation spectra were acquired on an Agilent Technologies Carry Eclipse Fluorescence Spectrophotometer, in quartz cuvettes with a path length of 1 cm. Emission and excitation spectra were collected with a scan rate of 120.0 nm / min, a delay interval of 1.0 nm and band-passes of 5 nm unless stated otherwise. FRET efficiency (E) was calculated according to **Equation 2**.

$$E\ [\%] = 1 - \left(\frac{A_D}{A_{DA}} x\ \frac{E_{DA}}{E_D}\right) x\ 100 \qquad \textbf{Eq 2.}$$

Where E is the FRET efficiency, $A_D$ and $E_D$ is the absorbance and the emission of the donor fluorophore respectively, and $A_{DA}$ and $E_{DA}$ are the absorbance and emission of the donor fluorophore in the presence of the acceptor. Porphyrin emission and excitation spectra after excitation at the coumarin donor fluorophore were recorded using a 395 nm filter to remove $2\lambda_{ex}$ light from the fluorescence emission and $\frac{\lambda_{em}}{2}$ from the excitation spectrum.

**Quantum yields:** The fluorescence quantum yields of **$L^1$-DME** and **$L^2$** were determined relative to tetraphenyl porphyrin (TPP) ($\phi_{514}$ = 0.11) in toluene.[1] Solutions of the reference and the sample were prepared so that the absorbance intensity at 514 nm was $\leq$ 0.1. All measurements were recorded under aerated conditions at room temperature in PBS buffer (pH = 7.4) and chloroform. Absorbance and emission spectrum were run consecutively with

identical instrumentation parameters. The quantum yield for the free-base and transition-metal-based porphyrins were calculated from **Equation 3**.

$$\emptyset_a = \left(\frac{I_a}{I_b}\right) x \left(\frac{A_b}{A_a}\right) x \left(\frac{n_a}{n_b}\right)^2 x \, \emptyset_b \qquad \textbf{Eq 3.}$$

Where $\emptyset$ is the quantum yield, I is the integrated intensity of the emission spectrum, A is the absorbance at the excitation wavelength and n is the refractive index of the solvent. 'a' refers to the sample, and 'b' refers to the standard.

**pH measurements:** Measurements were recorded using a Jenway 3510 pH meter in combination with a Jenway 924 005 pH electrode. Before each independent titration, the pH probe was calibrated using pH 4, 7 and 10 buffer solutions. Aqueous solutions containing Tris (25 mM) and NaCl (150 mM) were made up at different physiologically relevant pH values, and the pH of each solution was confirmed following the addition of **Fe–L$^1$** and **(1)**.

**Photobleaching experiment:** Measurements were recorded on a Beckman Coulter PARADIGM Microplate Reader in $H_2O$ and potassium phosphate buffer, Tris buffer and MOPS at pH = 7.4. Compound **(1)** (concentration 25 $\mu$M) was excited at 360 nm every 10 mins for 18 h. The emission was recorded at 460 nm.

## 2.2 Biological evaluation

**Overexpression and partial purification of human heme oxygenase-1**: A soluble form of hHO-1 lacking the C-terminal 23 residues (a kind gift from Dr Paul Sigala) was overexpressed in *Escherichia coli* BL21 (DE3) cells (Sigma). Starter cultures were grown in LB medium supplemented with 100 $\mu$g/ml ampicillin at 37 °C and 200rpm, until the optical density at a wavelength of 600 nm reached 0.5 and induced with 1 mM isopropyl β-D-1-thiogalactopyranoside. Cells were further incubated at 37 °C and 200 rpm for 5 h before being harvested by centrifugation at 4000 rcf for 15 min at 20 °C. Cell pellet was re-suspended in lysis buffer (20 mM Tris-HCl, pH 7.4, 150 mM NaCl) at 200 mg wet pellet per mL, and lysed

by sonication at wavelength of 15 $\mu$m (SoniPrep 150, MSEcheck). Cell debris were removed by centrifugation at 20000 *g* for 20 min at 4 °C and the supernatant fraction was used for HO-1 activity assays. HO-1 reactions were set up in lysis buffer containing 20 % v/v cleared *E. coli* lysate, 50 $\mu$M or 200 $\mu$M heme or probe, and 1 mM NADPH, and incubated away from light at 37 °C for 16h. Unreacted controls were set up in lysis buffer containing an equivalent amount of lysate and heme/probe, and measured without incubation or addition of NADPH .

**Absorbance measurements of HO-1 activity:** For UV-vis spectroscopy, reactions (200 $\mu$L) were carried out with 50 $\mu$M probe (heme, **Fe–L$^1$** or **Fe–L$^2$**) in 96-well microplates, and absorbance between 300 nm and 800 nm were measured at 1 nm intervals by a Beckman Coulter PARADIGM Microplate Reader. Absorbance spectra of heme, biliverdin, and probes standards (50 $\mu$M) were also recorded in the presence of 20% v/v *E. coli* lysate, as HO-1 binding alter the spectra of these compounds. Absorbance spectrum of 20 % v/v *E. coli* lysate in lysis buffer, without NADPH or probe, was recorded and subtracted as a baseline.

**Sample preparation for fluorescence measurements of HO-1 activity:** For fluorescence spectroscopy, reactions (500 $\mu$L) were carried out with 50 $\mu$M probes in microtubes. Prior to fluorescence measurements, samples were diluted with 200 $\mu$L of deionised water to increase the total sample volume from 500 $\mu$L to 700 $\mu$L.

**Fluorescence measurements of HO-1 activity:** Emission and excitation spectra of the HO-1 reactions set up with *E. coli* lysates were carried out in the same procedure as described in **Section 2.1** above.

**Sample preparation for mass spectrometry of HO-1 reactions:** Samples from the HO-1 reactions (containing 200 $\mu$M probe) were lyophilised on an Alpha 1-2LD plus -55 °C freeze

dryer overnight and subsequently acidified in $H_2SO_4$ / MeOH (5 % v/v, 400 $\mu$L). Each sample was subsequently stirred with a VWR International mini vortex mixer for 30 seconds and left to stand at 298 K for 24 h. After this time, samples were diluted with $CHCl_3$ (800 $\mu$L) and the organic phase was washed with deionised water (3 x 400 $\mu$L) and centrifuged to ensure a good separation between the two layers was achieved. The organic and aqueous layers were then separated and the organic solvent was evaporated under atmospheric pressure overnight at 298 K. Mass spec samples were submitted as solid samples for MALDI-MS and run by the Mass Spectrometry service at Imperial College London.

**LC-MS of HO-1 reaction samples:** Organic extracts of the samples incubated with *E. coli* lysates were dissolved in 25 % DMSO in acetonitrile to ensure complete solubility (total volume = 100 $\mu$L). LC-MS measurements were recorded on an Agilent 6200 TOF LC-MS from a gradient of 20 % – 98 % – 20 % acetonitrile in water (2 % formic acid). Coumarin **(12)** was ran under the same conditions and used as a control. Retention time ($R_T$) is quoted in minutes (min). UV detection was recorded at 320 nm, the absorbance maximum of coumarin methyl ester **(12)** and break-apart product **(1).**

**Preparation of human blood-derived macrophages:**

Human blood-derived macrophages were prepared from normal human volunteers exactly as previously described (PMID: 32667970).

Briefly, favourable institutional Ethical opinion and consent were obtained, 50 mL of blood aseptically withdrawn. Peripheral blood mononuclear cells were separated by Ficoll-Hypaque and cultured at 2 x $10^5$ cells per well in a 24-well plate (1.8 $cm^2$ culture surface per well). Macrophages were cultured overnight in 10% autologous serum and IMDM and then stimulated with hemin 20 µM (Sigma Aldrich 51280, concentration chosen for pathophysiological representativeness).

The macrophages were lysed in ultrapure water 100 µL / well (Sigma Aldrich W4502) and frozen at -80 °C until analysis. Defrosted lysates of 16 wells were scraped with a silicone policeman, pooled and added to 60 wells of a 96-well plate, giving 5.3 x $10^4$ cells per well equivalent.

Whole lysates of macrophages were incubated in a reaction mix with 50 µM Fe-L1; 20 µL macrophage hypotonic lysate (1.7 mg.$mL^{-1}$ protein at A280 nm); 80 µL 1mM MOPS pH 7.4 (3-(*N*-morpholino)propanesulfonic acid, Merck Sigma-Aldrich, M1254); final 150 µM NADPH (10 µl / well Nicotinamide Adenine Dinucleotide Phosphate 2 mM Enzo ALX-480-004-M050); 10 µL/well Catalase 1mg/ml 2700 u/mg (Tokyo Chemical Industries TCI C0052).

Fluorescence was then measured in a white Fluoronunc 96-well plate using a Biotek Synergy HT multimode plate reader, at 37 °C, measuring top fluorescence every 10 minutes, sensitivity settings auto low well = 25, excitation 360/40, emission 460/40, for 18 hours total. Reaction mix was collected at the end, pooled and analysed with fluorescence spectroscopy.

## 2.3 Compound synthesis

Compounds **(2)**,[2] **(5)**,[3] **(7)**,[4] and **(9)**[5] were prepared using procedures previously published in the literature.

**4-(2-((4-Methyl-2-oxo-2*H*-chromen-7-yl)oxy)ethoxy)benzoic acid, (1)**

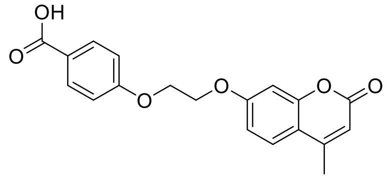

NaOH (4 M, 0.7 mL) was added to a solution of coumarin **(11)** (74.8 mg, 0.20 mmol) in ethanol (5 mL). The solution was heated at 85 °C for 20 h, with the formation of a white precipitate. The precipitate was dissolved in deionised water (5 mL) and acidified to pH 4-5 with 6 M HCl. The white precipitate formed on acidification was isolated by centrifuge, washed with water (3 x 10 mL) and dried under reduced pressure to form the title compound as a white solid (63.8 mg, 94 %). $^1$H NMR (400 MHz, $(CH_3)_2SO$) 12.60 (1 H, br s), 7.92 – 7.88 (2 H, m), 7.70 (1 H, d,

$^{3}J_{H\text{-}H}$ 8.8), 7.10 – 7.06 (3 H, m), 7.02 (1 H, dd, $^{3}J_{H\text{-}H}$ 8.9 and $^{4}J_{H\text{-}H}$ 2.5), 6.22 (1 H, d, $^{4}J_{H\text{-}H}$ 1.3), 4.48 – 4.42 (4 H, m), 2.40 (3 H, d, $^{4}J_{H\text{-}H}$ 1.3); $^{13}C$ {$^{1}H$} (101 MHz, $(CH_3)_2SO$) 167.0, 161.8, 161.3, 160.1, 154.7, 153.4, 131.4, 126.5, 123.3, 114.4, 113.3, 112.5, 111.3, 101.4, 67.0, 66.4, 18.2; ESI-LRMS $[C_{19}H_{17}O_6]^+$, (+) m/z 341.1; ESI-HRMS calculated for $[C_{19}H_{17}O_6]^+$, 341.1025 found, 341.1024.

**Dibenzyl-5,5'-(4-hyroxyphenyl)methylene)bis(3,4-dimethyl-1H-pyrrole-2-carboxylate), (3).**

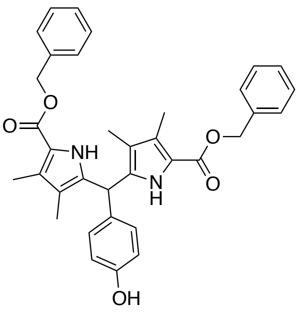


K-10 montmorillonite clay (0.86 g) was added to a solution of **(2)** (213.7 mg, 0.93 mmol) in anhydrous $CH_2Cl_2$ (12 mL). 4-Hydroxybenzaldehyde (60.3 mg, 0.49 mmol) and TFA (1.0 mL, 5.76 mmol) were added, and the suspension was stirred at room temperature for 2 d. The suspension was diluted with $CH_2Cl_2$ (10 mL) and insoluble inorganics were removed by filtration, and washed $CH_2Cl_2$ (2 x 10 mL). Organic extracts were combined and washed with $NaHCO_3$ (saturated solution, 20 mL) and brine (20 mL) and dried over $Na_2SO_4$. The solvent was removed under reduced pressure to form a dark red solid. Purification by silica gel column chromatography (gradient 100 % hexane to 60 % hexane, 40 % ethyl acetate) formed the title compound as a pale pink oil (180.9 mg, 66 %). $^{1}H$ NMR (400 MHz, $CDCl_3$) 8.40 (2 H, br s, -NH), 7.39 – 7.24 (10 H, m), 6.91 (2 H, d, $^{3}J_{H\text{-}H}$ 8.4), 6.74 (2 H, d, $^{3}J_{H\text{-}H}$ 8.4), 5.67 (1 H, br s, -OH), 5.41 (1 H, s), 5.26 (4 H, s), 2.25 (6 H, s), 1.77 (6 H, s); $^{13}C$ {$^{1}H$} NMR (101 MHz, $CDCl_3$) 161.7, 155.2, 136.5, 132.6, 130.4, 129.5, 128.4, 128.0, 127.9, 117.9, 117.4, 116.1, 115.9, 65.7, 40.4, 10.8, 8.9; ESI-LRMS $[C_{35}H_{35}N_2O_5]^+$, (+) m/z 563.3, ESI-HRMS calculated for $[C_{35}H_{35}N_2O_5]^+$, 563.2546 found, 563.2554.

**5,5'-(4-Hyroxyphenyl)methylene)bis(3,4-dimethyl-1H-pyrrole-2-carboxylic acid), (4).**

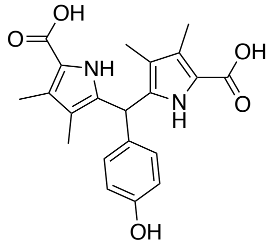

Palladium on charcoal (10 wt%, 112.4 mg, 0.11 mmol) was added to a solution of compound **(3)** (492.6 mg, 0.88 mmol) in ethanol (30 mL) and the reaction was stirred at room temperature for 4 h under an atmosphere of hydrogen. After this time, the reaction mixture was filtered through a celite plug, and washed with ethanol (20 mL) and $CH_2Cl_2$ (20 mL). The solvent was removed under reduced pressure to form a purple oil / solid (287.5 mg, 85 %). $^{1}H$ NMR (400 MHz, MeOD) 6.88 (2 H, d, $^{3}J_{H-H}$ 6.9), 6.74 (2 H, d, $^{3}J_{H-H}$ 6.9), 5.56 (1 H, s), 2.25 (6 H, s), 1.81 (6 H, s); $^{13}C$ {$^{1}H$} NMR (101 MHz, MeOD) 163.1, 156.2, 133.1, 130.4, 129.4, 129.0, 127.6, 117.3, 115.1, 39.6, 9.4, 7.5; ESI-LRMS $[C_{21}H_{21}N_2O_5]^-$, (-) m/z 381.1, ESI-HRMS calculated for $[C_{21}H_{21}N_2O_5]^-$, 381.1450 found, 381.1448.

**Dimethyl-3,3'-(10-(4-hydroxyphenyl)-3,7,8,12,13,17-hexa-methylporphyrin-2,18-diyl)dipropionate, (6)**

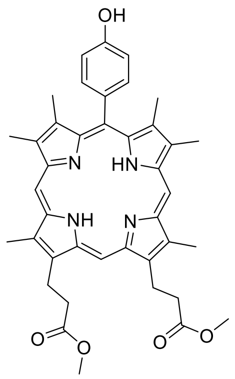

Compound **(4)** (423.2 mg, 1.11 mmol) was added to a solution of **(5)** (456.4 mg, 1.11 mmol) in anhydrous $CH_2Cl_2$ (150 mL). A solution of *p*-toluene sulfonic acid (758.5 mg, 3.99 mmol) in methanol (20 mL) was added dropwise over 15 min and the solution was stirred at room temperature overnight with protection from light under an inert atmosphere of nitrogen. After 18 h, *p*-chloranil (545.2 mg, 2.22 mmol) was added and the reaction was stirred for an additional 2 h, before the solvent was removed under reduced pressure to form a dark red residue. Purification silica gel column chromatography (gradient 100 % $CH_2Cl_2$ to 95 % $CH_2Cl_2$ / 5 % MeOH) formed the title compound as a dark red solid (344.8 mg, 46 %). $^{1}H$ NMR (400 MHz, $CDCl_3$) 10.13 (2 H, s), 9.96 (1 H, s), 7.06 (2 H, d, $^{3}J_{H-H}$ 7.9), 6.68 (2 H, $^{3}J_{H-H}$ 7.9), 4.42 (4 H, t, $^{3}J_{H-H}$ 7.7), 3.70 (6 H, s), 3.67 (6 H, s), 3.46 (6 H, s), 3.34 (4 H, t, $^{3}J_{H-H}$ 7.7), 2.10 (6 H, s); $^{13}C$ {$^{1}H$} (101 MHz, $CDCl_3$) 173.8, 158.1, 156.1, 144.9, 144.8, 143.0, 137.8, 137.6, 137.2, 136.9, 133.7, 118.5, 114.2, 96.8, 95.2, 51.8, 37.0, 22.0, 14.9, 12.1, 11.8; ESI-

LRMS $[C_{40}H_{43}N_4O_5]^+$, (+) m/z 659.3, ESI-HRMS calculated for $[C_{40}H_{43}N_4O_5]^+$, 659.3233 found, 659.3245; UV-Vis ($CHCl_3$, $\lambda_{max}$ / nm): ($\varepsilon$ / $M^{-1}cm^{-1}$) 405 (100179), 504 (14248), 537 (8943), 571 (7123), 624 (3287).

**Dimethyl 3,3'-(3,7,8,12,13,17-hexamethyl-10-(4-(2-((4-methyl-2-oxo-2*H*-chromen-7-yl)oxy)ethox-y) phenyl)porp-hyrin-2,18-diyl)dipropionate, L$^1$–DME, (8)**

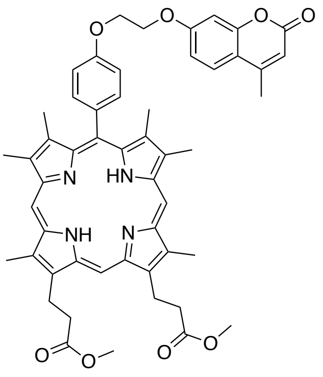


Anhydrous $K_2CO_3$ (56.2 mg, 0.41 mmol) was added to a solution of **(6)** (26.2 mg, 0.04 mmol) and **(7)** (26.3 mg, 0.09 mmol) in anhydrous DMF (2.5 mL). The reaction was stirred at room temperature under an inert atmosphere of nitrogen with protection from light for 4 d. The solvent was removed under reduced pressure to form a dark brown residue. Purification by silica gel column chromatography (gradient 100 % $CH_2Cl_2$ to 99 % $CH_2Cl_2$ / 1 % MeOH) formed the title compound as a dark red solid (23.5 mg, 68 %). $^1$H NMR (400 MHz, $CDCl_3$) 10.15 (2 H, s), 9.95 (1 H, s), 7.77 – 7.74 (2 H, m), 7.52 – 7.44 (1 H, m), 7.10 – 7.05 (2 H, m), 6.94 – 6.90 (2 H, m), 6.17 (1 H, br s), 4.44 – 4.33 (8 H, m), 3.68 (6 H, s), 3.65 (6 H, s), 3.52 (6 H, s), 3.30 (4 H, t, $^3J_{H-H}$ 7.8), 2.41 – 2.30 (9 H, m); $^{13}$C {$^1$H} (101 MHz, $CDCl_3$) 173.7, 161.6, 161.3, 158.7, 155.2, 152.5, 145.1, 144.9, 143.5, 143.0, 138.1, 137.8, 137.2, 137.0, 135.5, 133.8, 125.6, 118.5, 113.9, 113.6, 112.7, 112.2, 101.6, 96.86, 95.4, 67.1, 66.3, 51.8, 37.0, 21.9, 18.7, 15.2, 12.2, 11.8; ESI-LRMS $[C_{52}H_{53}N_4O_8]^+$, (+) m/z 861.4, ESI-HRMS calculated for $[C_{52}H_{53}N_4O_8]^+$, 861.3863 found, 861.3887; UV-Vis ($CHCl_3$, $\lambda_{max}$ / nm): ($\varepsilon$ / $M^{-1}cm^{-1}$) 291 (14481.4), 321 (23963.1), 405 (113969.6), 503 (10264.3), 538 (5047.8), 572 (4529.2), 625 (1601.4).

**Fe–L[1]–dimethyl ester, Fe–L[1]–DME**

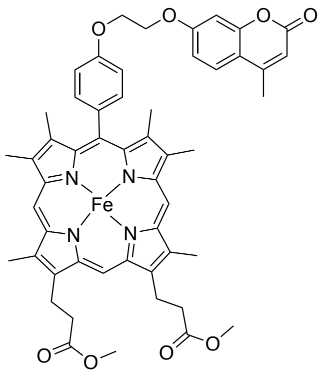


$FeCl_2.4H_2O$ (85.2 mg, 0.43 mmol), sodium hydrogen carbonate (30.2 mg, 0.36 mmol) and sodium ascorbate (21.0 mg, 0.11 mmol) were added to a solution of **(8)** (42.2 mg, 0.05 mmol) in $CHCl_3$ / MeOH (2:1, 20 mL). The solution was heated to 60 °C and stirred under an inert atmosphere of nitrogen with protection from light. After 18 h, the reaction was cooled to room temperature and the solvent was removed under reduced pressure. The crude residue was re-dissolved in $CH_2Cl_2$ (20 mL) and washed with $H_2O$ (10 mL) and brine (10 mL). Organic extracts were combined and dried over $Na_2SO_4$, and the solvent was removed under reduced pressure to form a dark red / brown residue. Purification by silica gel column chromatography (gradient 100 % $CH_2Cl_2$ to 95 % $CH_2Cl_2$ / 5 % MeOH) formed the title compound as a dark red / brown solid (38.1 mg, 85 %). MALDI-MS 914.8; UV-Vis ($CHCl_3$, $\lambda_{max}$ / nm): ($\varepsilon$ x$10^4$ / $M^{-1}cm^{-1}$) 290 (28315.0), 321 (34896.4), 384 (58787.9), 506 (8568.1), 535 (7306.8), 638 (2916.6).

**Fe–L[1]**

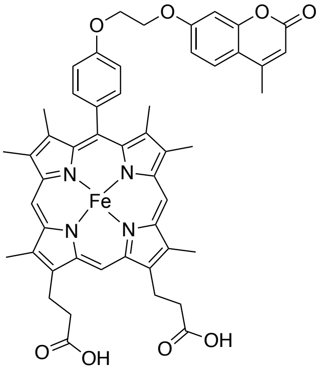


NaOH (4 M, 0.45 mL) was added to a solution of **Fe–L[1]–DME** in $CH_2Cl_2$ (1 mL) and methanol (3 mL). The solution was heated to 40 °C and stirred under an inert atmosphere of nitrogen for 24 h. During the reaction a dark red / brown precipitate appeared indicating the formation of **Fe–L[1]**. After 24 h the reaction was cooled to room temperature and the solvent was removed under reduced pressure to form a dark brown residue. The residue was re-dissolved in $H_2O$ (5 mL) and acidified to pH 3-4 with HCl (6 M). The precipitate was isolated by centrifuge, washed with $H_2O$ (3 x 10 mL) and lypholised to form the title compound as a dark purple / brown solid (12.6 mg, 72 %). ESI-LRMS $[C_{50}H_{46}N_4O_8Fe]^+$, (+) m/z 886.3; ESI-HRMS calculated for

$[C_{50}H_{46}N_4O_8Fe]^+$, 886.2665 found, 886.2682; MALDI-MS 886.2; UV-Vis ($CHCl_3$, $\lambda_{max}$ / nm): ($\varepsilon$ / $M^{-1}cm^{-1}$) 320 (19980.8), 385 (59877.1), 523 (4975.3), 548 (4183.6), 640 (2615.6).

**4-Methyl-7-(2-(4-(10,15,20-triphenylporphyrin-5-yl)phenoxy)ethoxy)-2*H*-chromen-2-one, $L^3$, (10).**

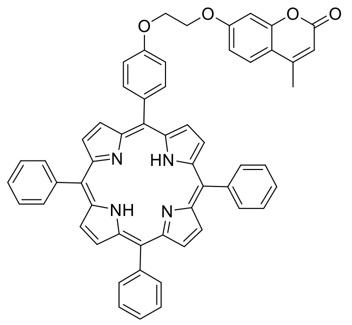

Anhydrous $K_2CO_3$ (281.8 mg, 2.04 mmol) was added to a solution of **(9)** (105.5 mg, 0.17 mmol) and **(7)** (111.0 mg, 0.39 mmol) in anhydrous DMF (9.5 mL). The reaction was stirred at room temperature under an inert atmosphere of nitrogen with protection from light for 3 d. The solvent was removed under reduced pressure to form a dark brown residue. Purification by silica gel column chromatography (gradient 100 % hexane to 75 % hexane / 25 % ethyl acetate) formed the title compound as a dark purple solid (70.5 mg, 52 %). $^1H$ NMR (400 MHz, $CDCl_3$) 8.88 – 8.85 (8 H, m), 8.24 – 8.21 (6 H, m), 8.14 (2 H, d, $^3J_{H-H}$ 8.2), 7.81 – 7.77 (9 H, m), 7.55 (2 H, d, $^3J_{H-H}$ 8.5), 7.32 (2 H, d, $^3J_{H-H}$ 8.2), 7.03 – 6.99 (2 H, m), 6.18 (1 H, d, $^4J_{H-H}$ 1.3), 4.63 – 4.51 (4 H, m), 2.42 (3 H, d, $^4J_{H-H}$ 1.3), -2.77 (2 H, br s); ESI-LRMS $[C_{56}H_{41}N_5O_4]^+$, (+) m/z 833.3; ESI-HRMS calculated for $[C_{56}H_{41}N_5O_4]^+$, 833.3122 found, 833.3101; UV-Vis ($CHCl_3$, $\lambda_{max}$ / nm): ($\varepsilon$ $M^{-1}cm^{-1}$) 290 (9317.9), 320 (11098.0), 419 (175515.7), 516 (6766.15), 552 (3239.92), 592 (2091.9), 649 (2520.6).

**Fe–L[2]**

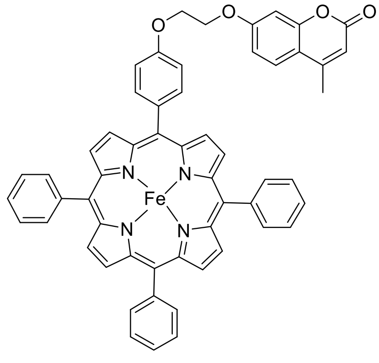


Fe-L[3]

$FeCl_2.4H_2O$ (60.2 mg, 0.30 mmol), sodium hydrogen carbonate (20.4 mg, 0.24 mmol) and sodium ascorbate (17.7 mg, 0.09 mmol) were added to a solution of **(10)** (41.7 mg, 0.05 mmol) in $CHCl_3$ / MeOH (2:1, 20 mL). The solution was heated to 60 °C and stirred under an inert atmosphere of nitrogen with protection from light. The solvent was removed under reduced pressure and the crude residue was re-dissolved in $CH_2Cl_2$ (20 mL) and washed with $H_2O$ (10 mL) and brine (10 mL). Organic extracts were combined and dried over $Na_2SO_4$, and the solvent was removed under reduced pressure to form a dark red / brown residue. Purification by silica gel column chromatography (gradient 100 % $CH_2Cl_2$ to 95 % $CH_2Cl_2$ / 5 % MeOH) formed the title compound as a dark red / brown solid (34.0 mg, 76 %). MALDI-MS 886.3; UV-Vis ($CHCl_3$, $\lambda_{max}$ / nm): ($\varepsilon$ / $M^{-1}cm^{-1}$) 320 (41179.2), 409 (99527.5), 572 (7982.8), 618 (4092.5).

**Ethyl 4-(2-((4-methyl-2-oxo-2*H*-chromen-7-yl)oxy)ethoxy)benzoate, (11)**

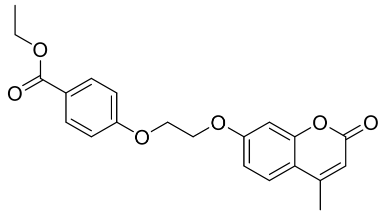


Coumarin **(7)** (165.2 mg, 0.58 mmol) was added to a stirring solution of ethyl-4-hydroxybenzoate (80.2 mg, 0.48 mmol), potassium carbonate (300.4 mg, 2.17 mmol) and potassium iodide (7.0 mg, 0.04 mmol) in anhydrous acetonitrile (7 mL). The reaction was heated to reflux and stirred for 24 h, before being cooled to room temperature. The suspension was diluted with $CH_2Cl_2$ (20 mL) and filtered to remove any inorganic impurities and the solvent was removed under reduced pressure to form an off white solid. Purification by column chromatography (gradient 100 % hexane to 100 % $CH_2Cl_2$) formed the title compound as a white crystalline solid (150.9 mg, 85 %). $^1H$ NMR (400 MHz, $CDCl_3$) 8.03 – 7.99 (2 H, m), 7.51 (1 H, d, $^3J_{H-H}$ 8.8), 6.98 – 6.95 (2 H, m), 6.92 (1 H, dd, $^3J_{H-H}$ 8.8 and $^4J_{H-H}$ 2.6), 6.87 (1 H, d, $^4J_{H-H}$ 2.6), 6.15 (1 H, d, $^4J_{H-H}$ 1.3) 4.40 (4 H, s), 4.34 (2 H, q,

$^{3}J_{H-H}$ 7.1), 2.40 (3 H, d, $^{4}J_{H-H}$ 1.3), 1.38 (3 H, t, $^{3}J_{H-H}$ 7.1); $^{13}C$ {$^{1}H$} (101 MHz, $CDCl_3$) 166.3, 162.1, 161.5, 161.2, 155.2, 152.5, 131.6, 125.7, 123.6, 114.2, 114.0, 112.7, 112.3, 101.6, 66.9, 66.3, 60.7, 18.7, 14.4; ESI-LRMS $[C_{21}H_{21}O_6]^+$, (+) m/z 369.1; ESI-HRMS calculated for $[C_{21}H_{21}O_6]^+$, 369.1333 found, 369.1324.

**Methyl 4-(2-((4-methyl-2-oxo-2*H*-chromen-7-yl)oxy)ethoxy)benzoate, (12)**

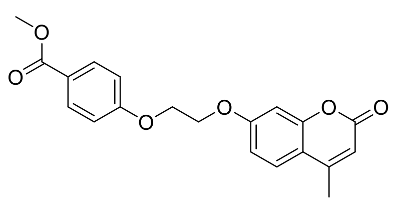

Coumarin **(7)** (239.2 mg, 0.84 mmol) was added to a stirring solution of methyl-4-hydroxybenzoate (104.6 mg, 0.69 mmol), potassium carbonate (554.0 mg, 4.0 mmol) and potassium iodide (12.1 mg, 0.07 mmol) in anhydrous acetonitrile (8 mL). The reaction was heated to reflux and stirred for 24 h, before being cooled to room temperature. The suspension was diluted with $CH_2Cl_2$ (20 mL) and filtered to remove any inorganic impurities and the solvent was removed under reduced pressure to form an off white solid. Purification by column chromatography (gradient 100 % hexane to 100 % $CH_2Cl_2$) formed the title compound as a white crystalline solid (186.4 mg, 77 %). $^{1}H$ NMR (400 MHz, $CDCl_3$) 8.05 – 8.01 (2 H, m), 7.52 (1 H, d, $^{3}J_{H-H}$ 8.7), 7.01 – 6.98 (2 H, m), 6.92 (1 H, dd, $^{3}J_{H-H}$ 8.7 and $^{4}J_{H-H}$ 2.4), 6.88 (1 H, d, $^{4}J_{H-H}$ 2.4), 6.16 (1 H, d, $^{4}J_{H-H}$ 1.3), 4.41 (4 H, s), 3.89 (3 H, s), 2.41 (3 H, d, $^{3}J_{H-H}$ 1.3); $^{13}C$ {$^{1}H$} (101 MHz, $CDCl_3$) 166.9, 162.3, 161.6, 161.3, 155.3, 155.6, 131.8, 125.8, 123,3, 114.3, 114.1, 112.8, 112.4, 101.7, 67.0, 66.4, 52.1, 18.8; ESI-LRMS $[C_{20}H_{19}O_6]^+$, (+) m/z 355.1; ESI-HRMS calculated for $[C_{20}H_{19}O_6]^+$, 355.1182 found, 355.1177.

## 3. Compound Characterisation

### 3.1 $^1$H and $^{13}$C NMR spectra

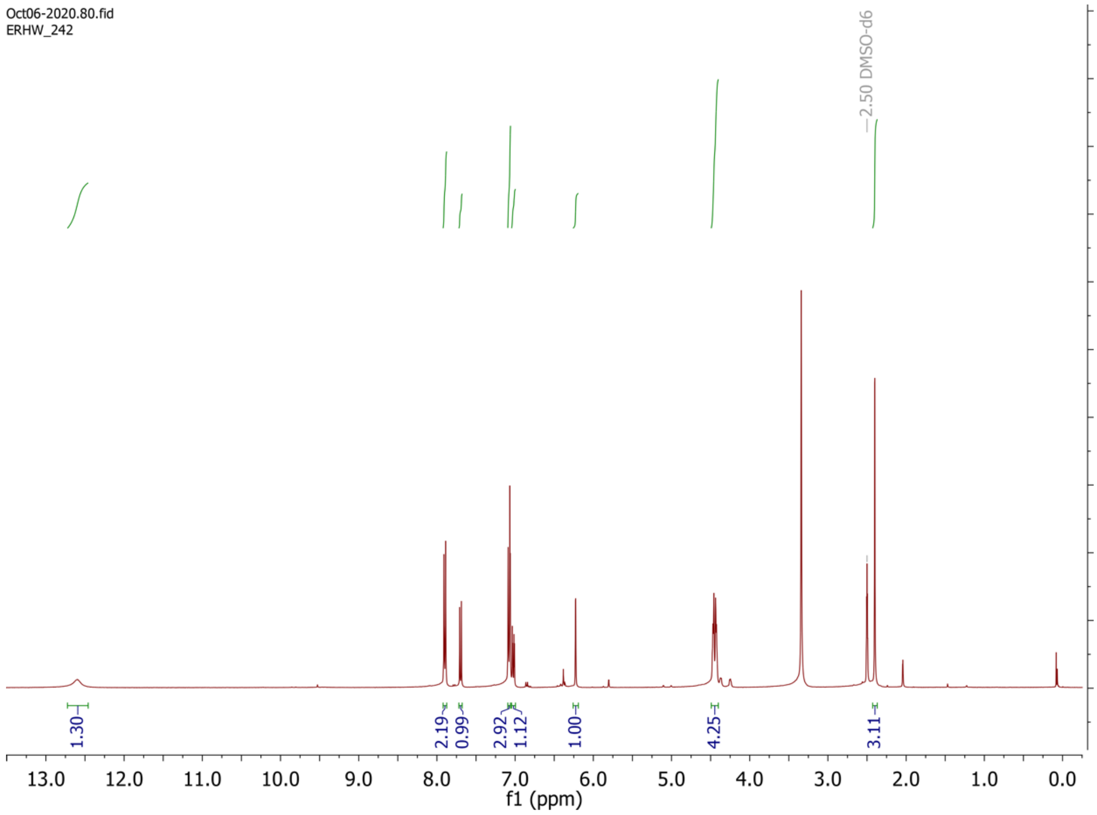


**Figure S23.** The $^1$H NMR spectra of compound **(1)** in $(CH_3)_2SO$, 298 K.

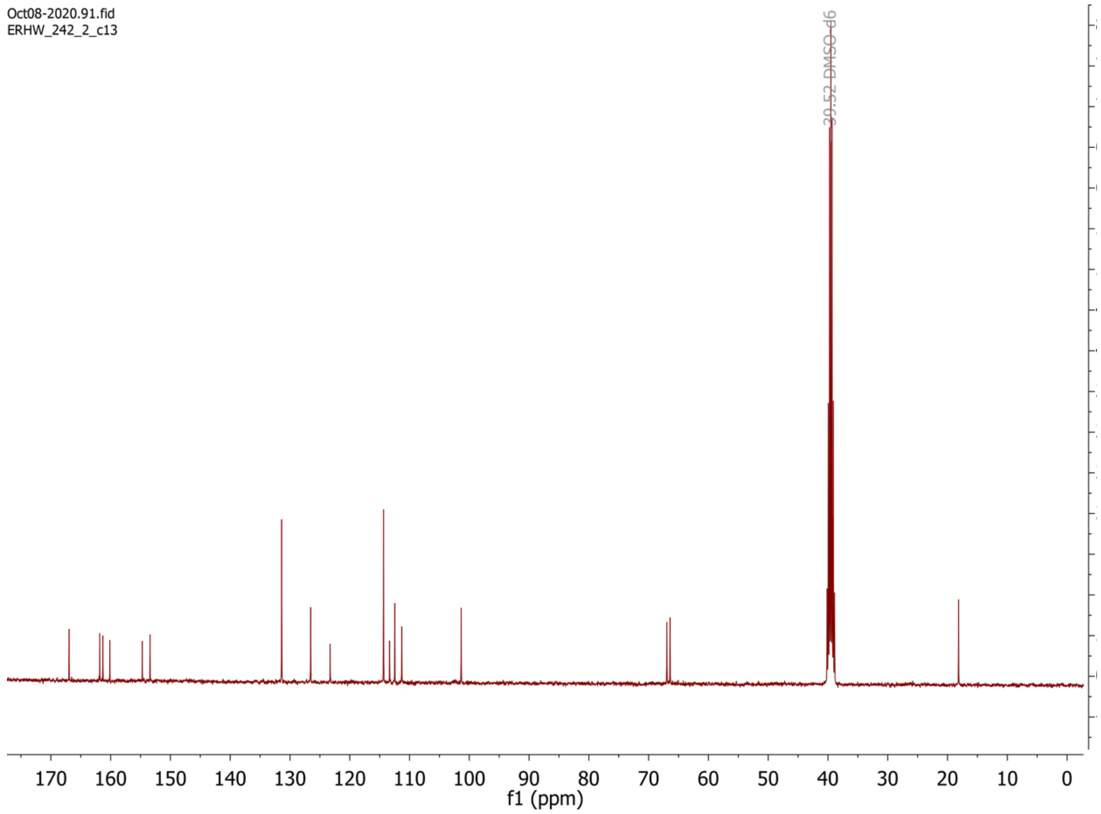


**Figure S24.** The $^{13}C$ NMR spectra of compound **(1)** in $(CH_3)_2SO$, 298 K.

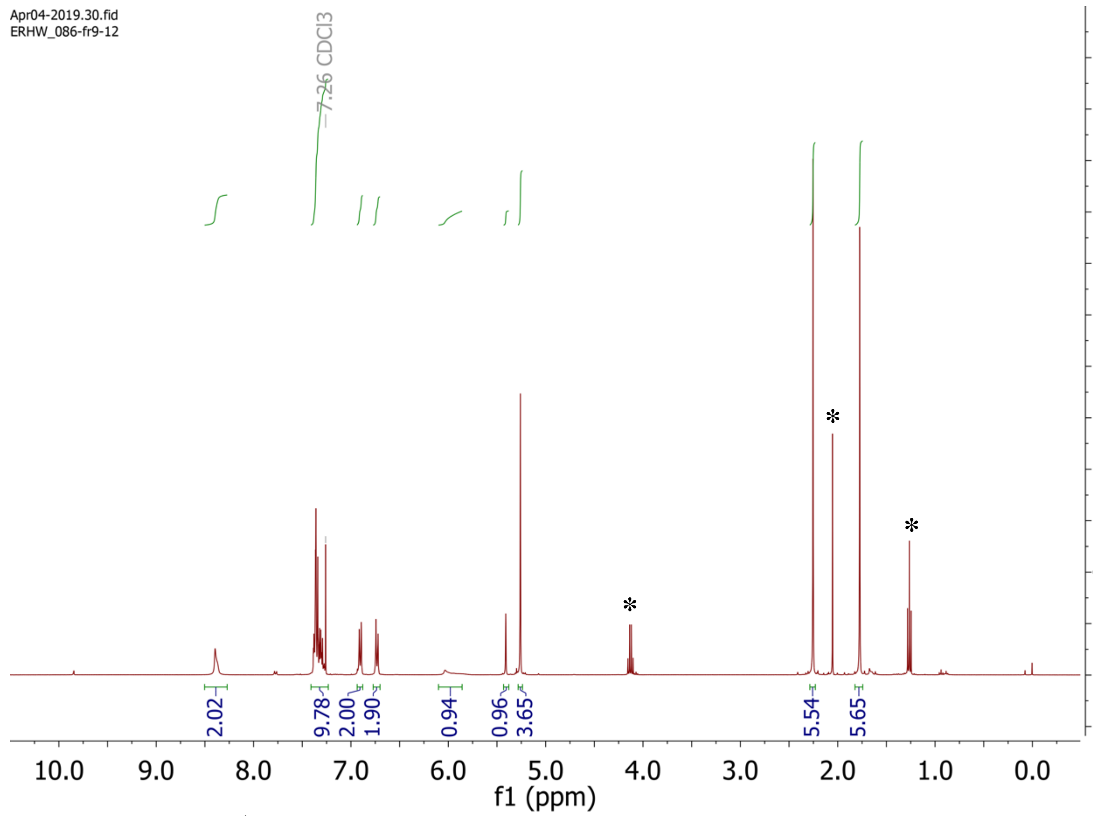


**Figure S25.** The $^{1}H$ NMR spectra of **(3)** in $CDCl_3$, 298 K. * Trace amounts of ethyl acetate.

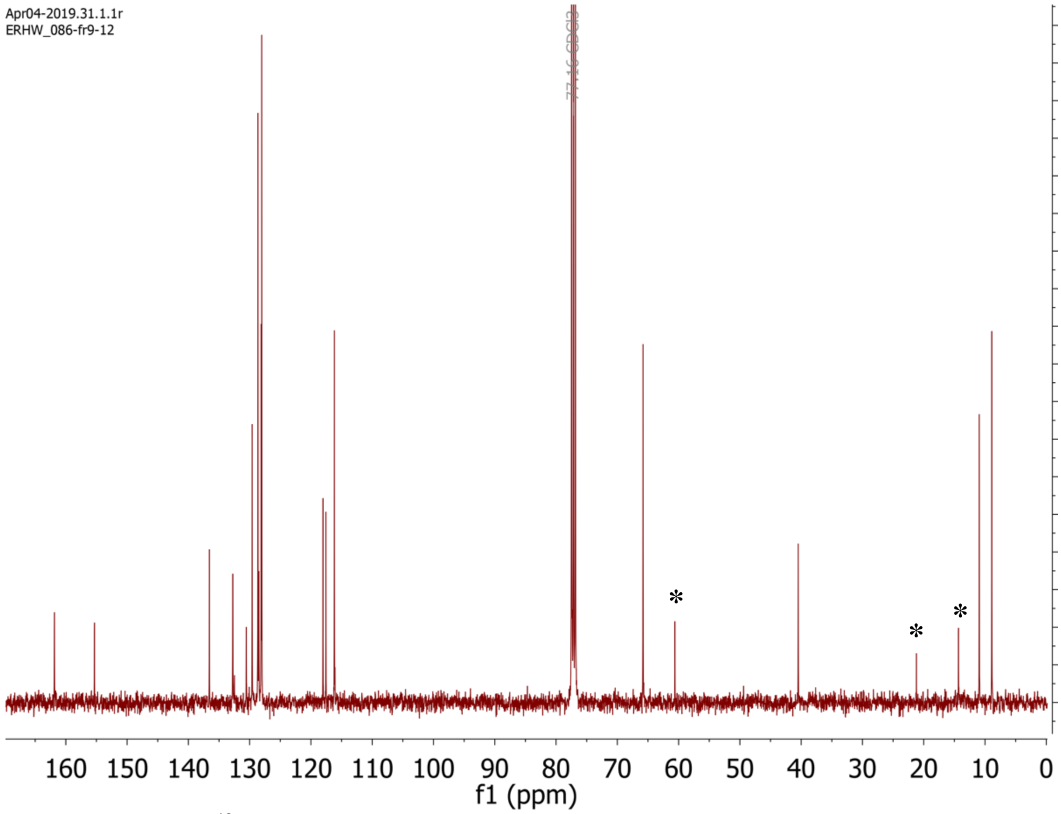


**Figure S26.** The $^{13}$C NMR spectra of **(3)** in $CDCl_3$, 298 K. * Trace amounts of ethyl acetate.

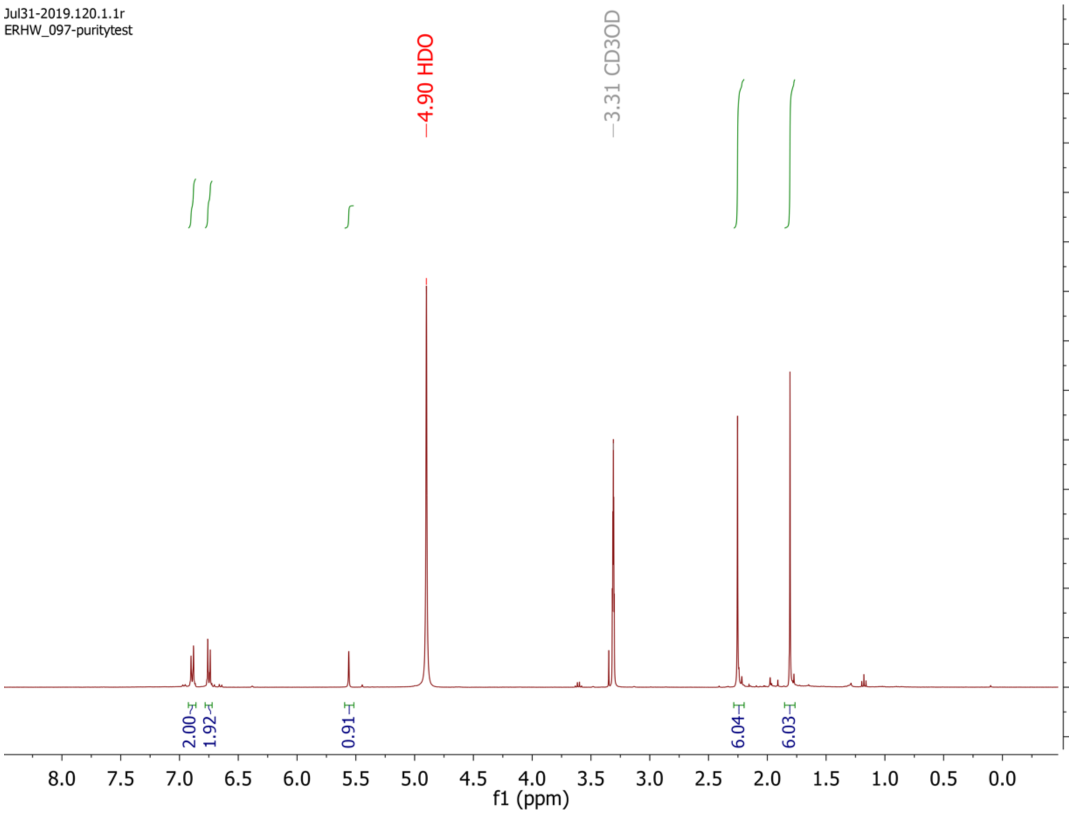


**Figure S27.** The $^{1}$H NMR spectra of **(4)** in MeOD, 298 K.

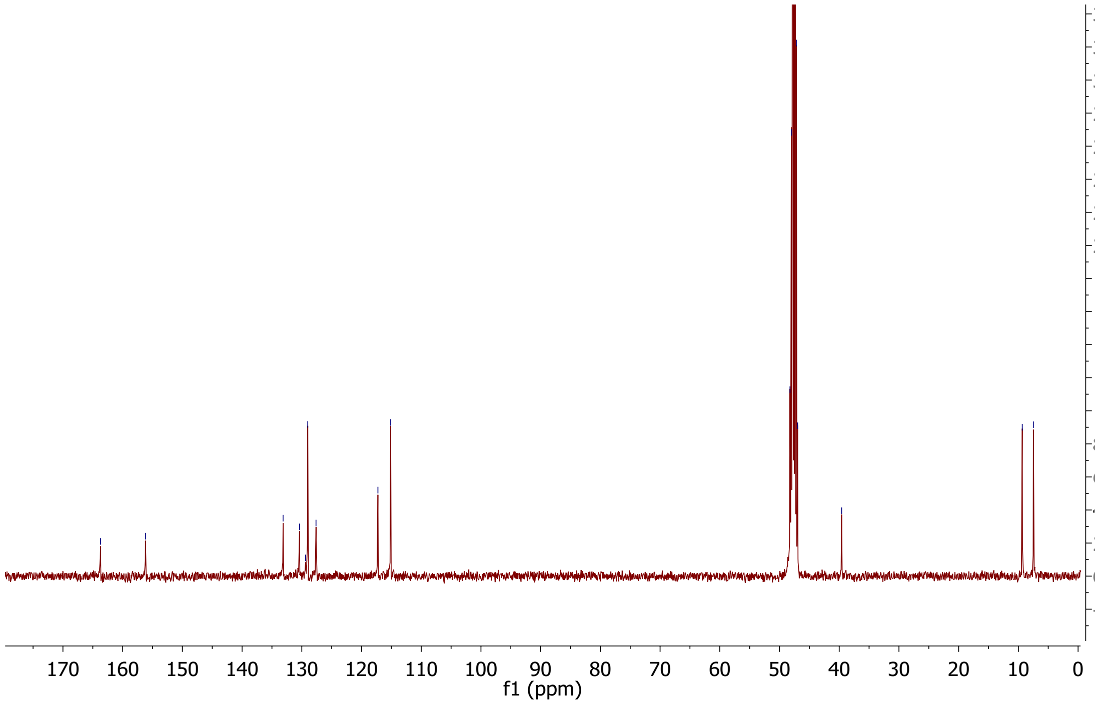


**Figure S28.** The $^{13}C$ NMR spectra of **(4)** in MeOD, 298 K.

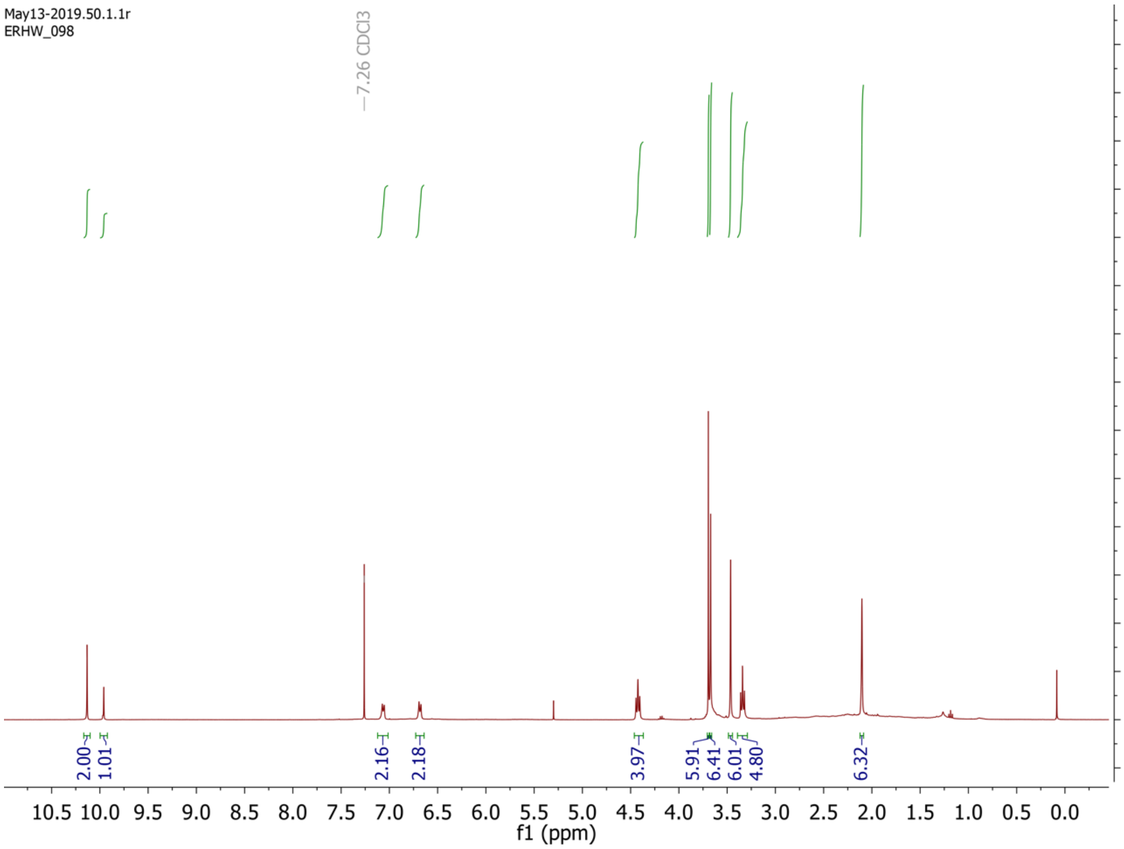


**Figure S29.** $^{1}H$ NMR spectra of **(6)** in $CDCl_3$, 298 K.

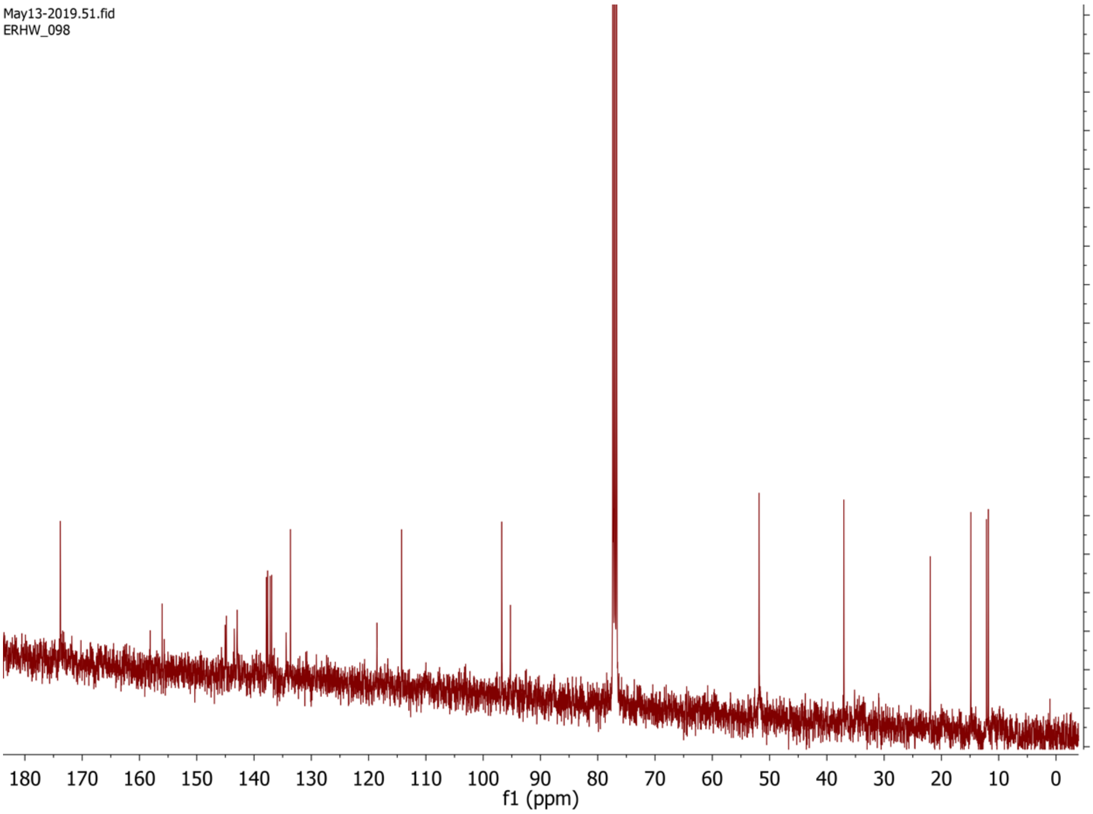


**Figure S30.** $^{13}$C NMR spectra of **(6)** in $CDCl_3$, 298 K.

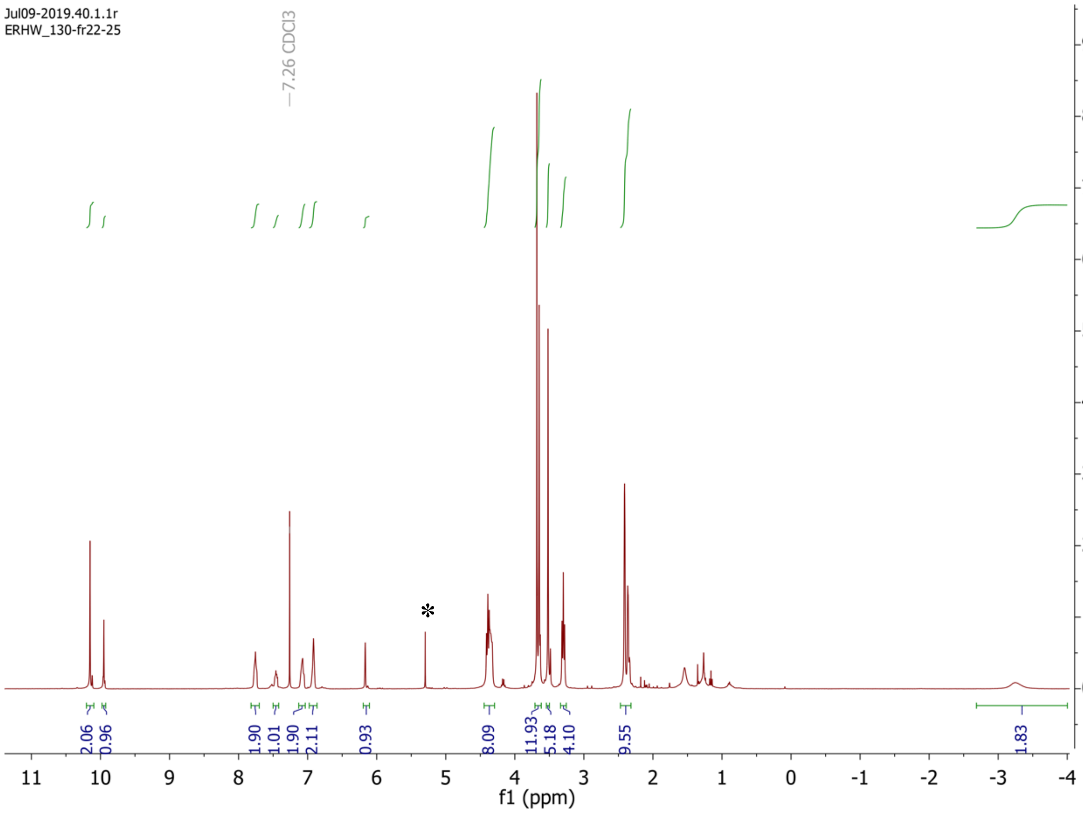


**Figure S31.** $^{1}$H NMR spectra of **L$^1$–DME** in $CDCl_3$, 298 K. * Trace amounts of $CH_2Cl_2$.

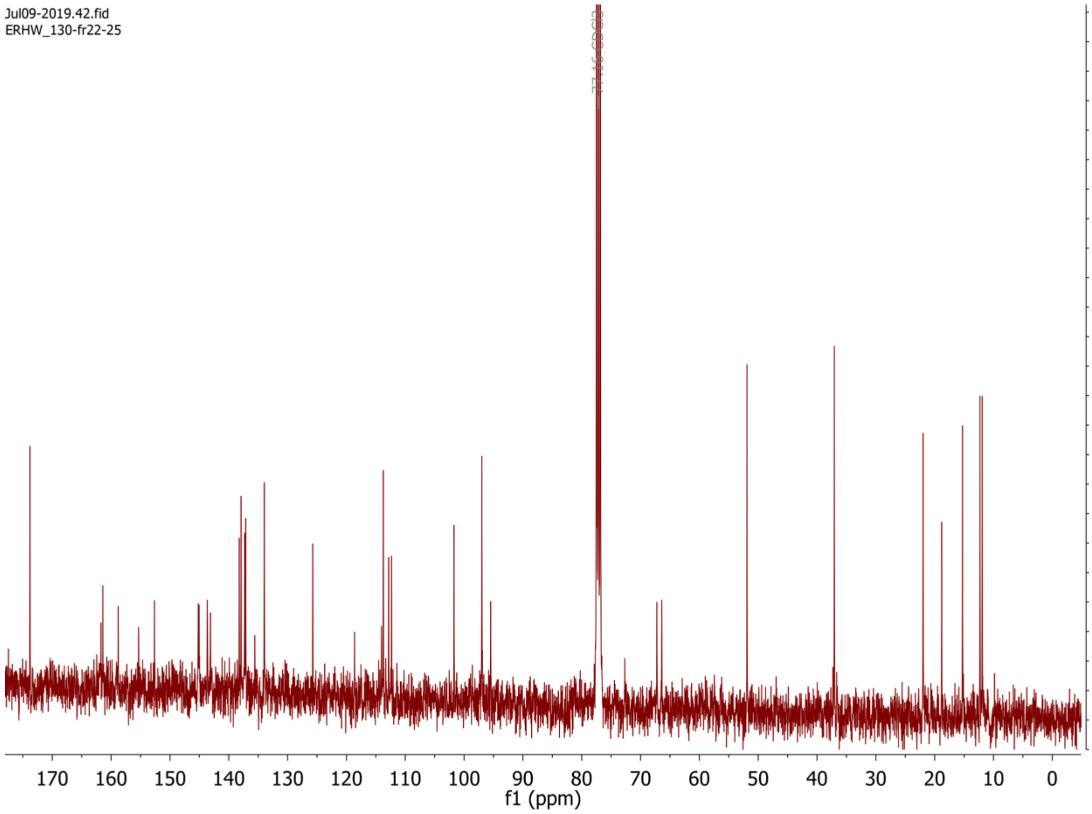


**Figure S32.** The $^{13}C$ NMR spectra of **$L^1$–DME** in $CDCl_3$, 298 K.

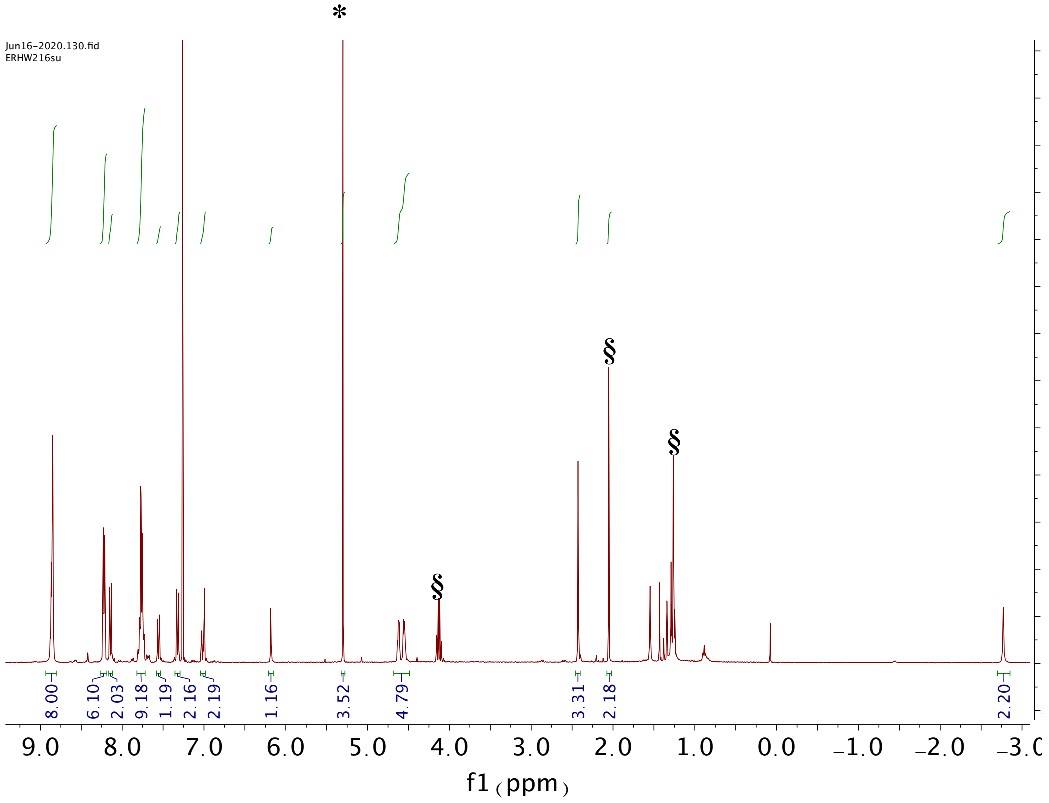


**Figure S33.** The $^{1}H$ NMR spectra of **$L^2$** in $CDCl_3$, 298 K. * Trace amounts of $CH_2Cl_2$, § trace amounts of ethyl acetate.

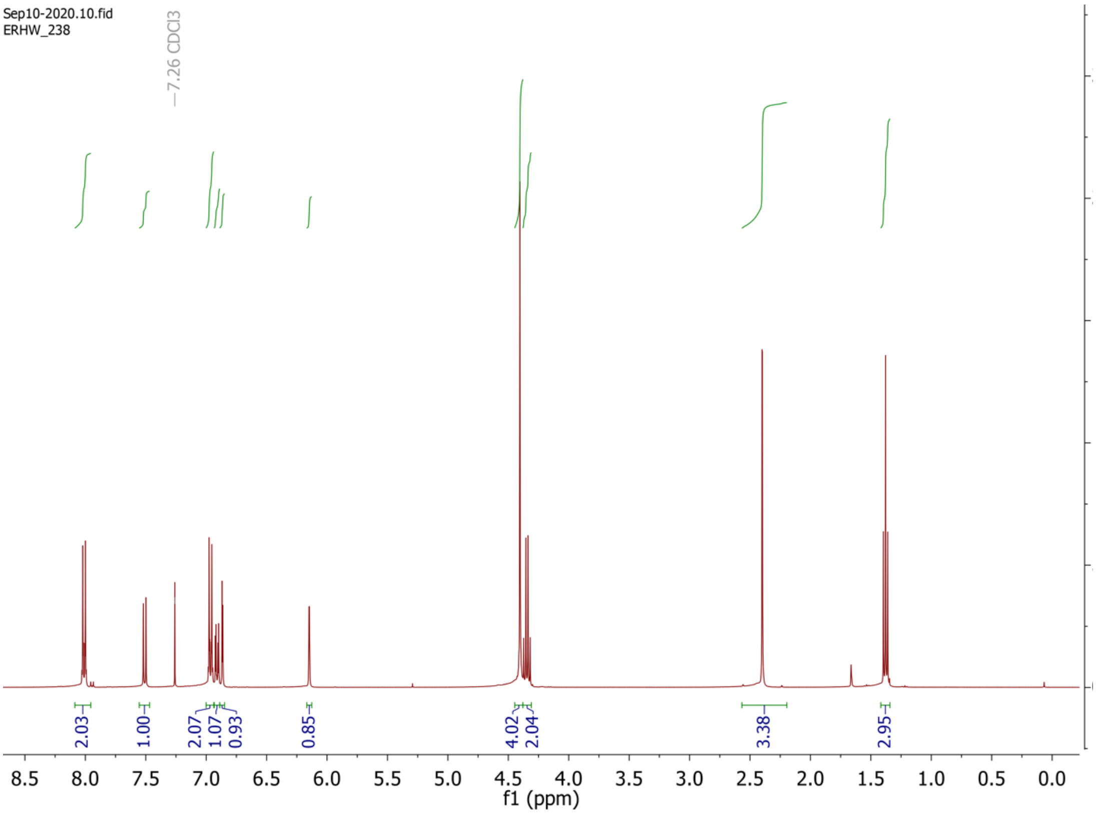


**Figure S34.** The $^{1}H$ NMR spectra of compound **(11)** in $CDCl_3$, 298 K.

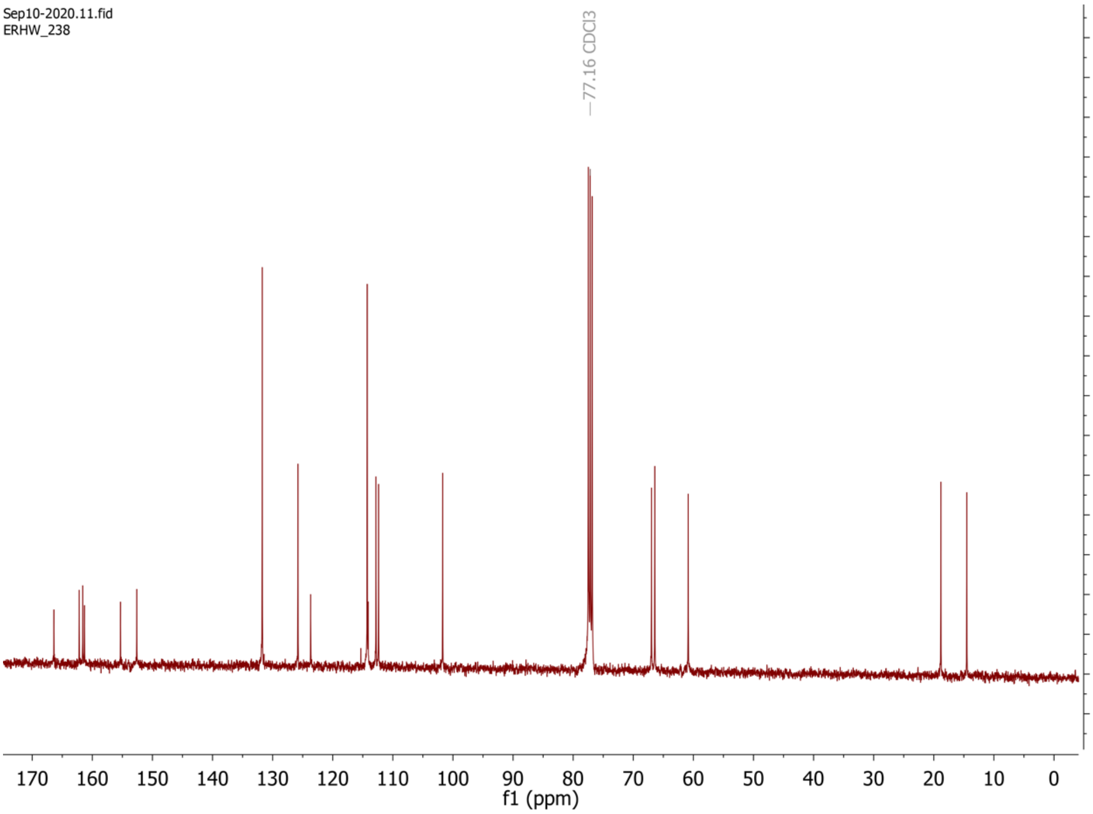


**Figure S35.** The $^{13}C$ NMR spectra of compound **(11)** in $CDCl_3$, 298 K.

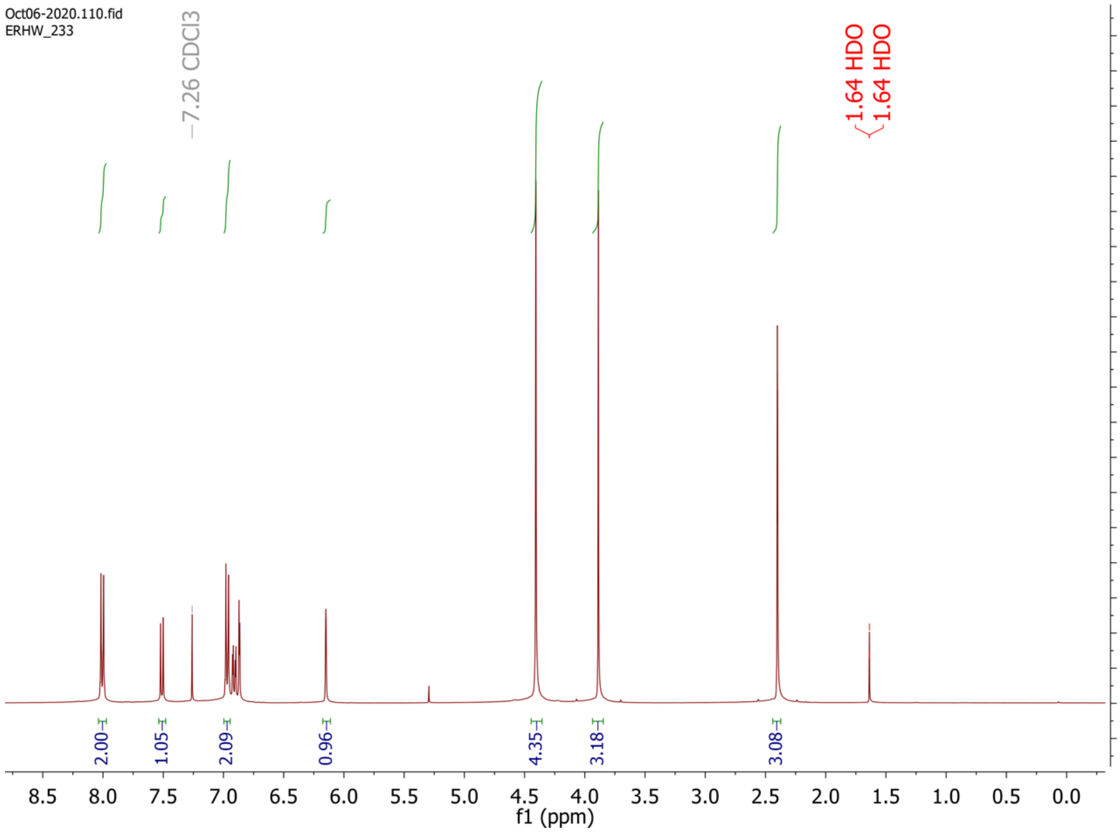


**Figure S36.** The $^{1}H$ NMR spectra of compound **(12)** in $CDCl_3$, 298 K.

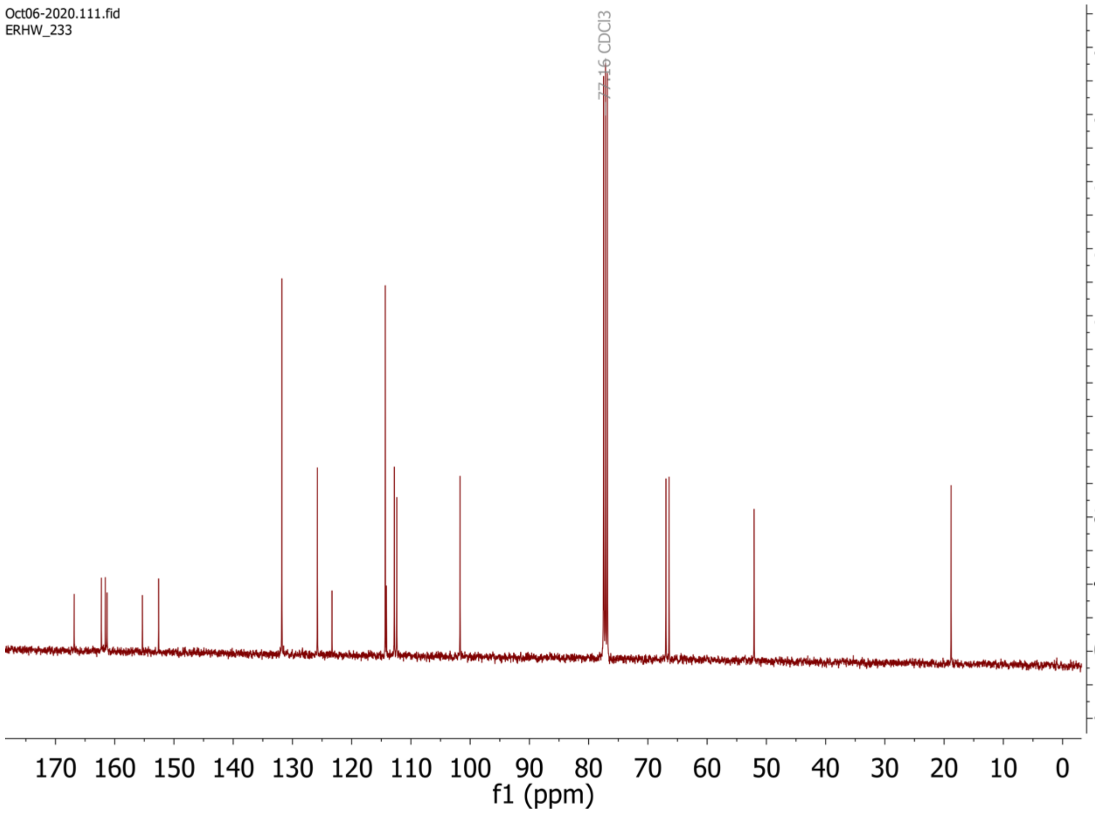


**Figure S37.** The $^{13}C$ NMR spectra of compound **(12)** in $CDCl_3$, 298 K.

## 3.2 Mass Spectrometry data

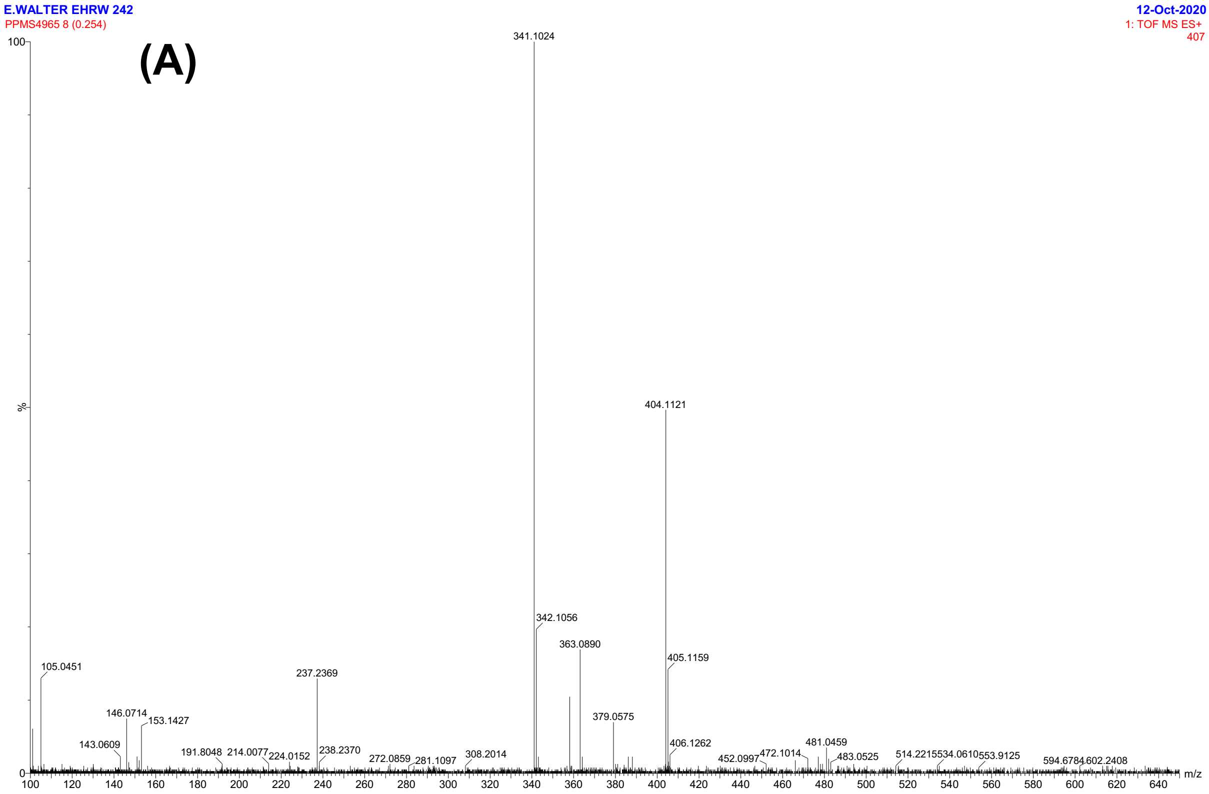


**(B)** Elements Used:

C: 19-19  H: 0-120  N: 0-15  O: 0-10

| | | | | | | |
|---|---|---|---|---|---|---|
| Minimum: | | | | -1.5 | | |
| Maximum: | | 5.0 | 10.0 | 1000.0 | | |
| Mass | Calc. Mass | mDa | PPM | DBE | i-FIT | Formula |
| 341.1024 | 341.1025 | -0.1 | -0.3 | 11.5 | 1.4 | C19 H17 O6 |

**Figure S38.** **(A)** The ESI-MS of **(1)** and **(B)** the calculated and found HRMS data.

**(A)**

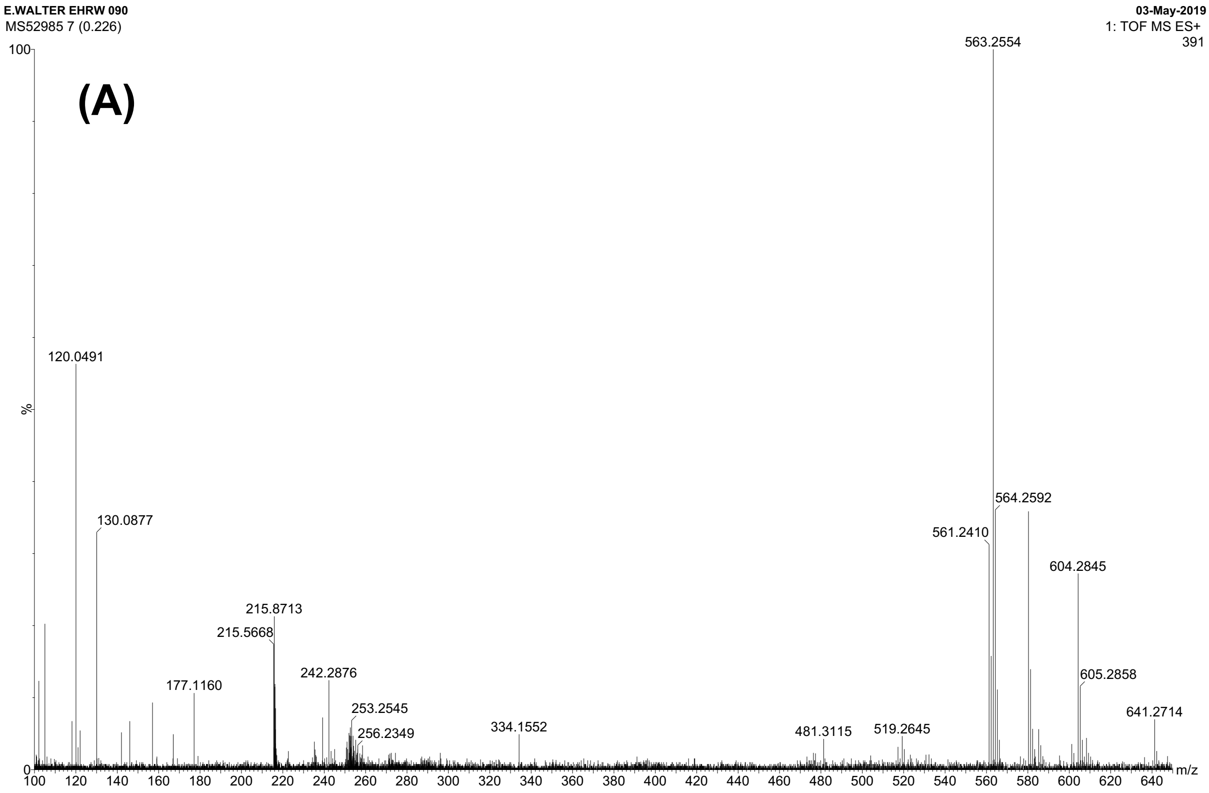


**(B)**

Elements Used:

C: 35-35 H: 0-100 N: 0-10 O: 0-10 Na: 0-1 194Pt: 0-2 195Pt: 0-2 196Pt: 0-2

| | | | | | | | |
|---|---|---|---|---|---|---|---|
| Minimum: | | | | -1.5 | | | |
| Maximum: | | 5.0 | 10.0 | 100.0 | | | |
| Mass | Calc. Mass | mDa | PPM | DBE | i-FIT | i-FIT (Norm) | Formula |
| 563.2554 | 563.2546 | 0.8 | 1.4 | 19.5 | 54.1 | 0.0 | C35 H35 N2 O5 |

**Figure S39. (A)** The ESI-MS of **(3)** and **(B)** the calculated and found HRMS data.

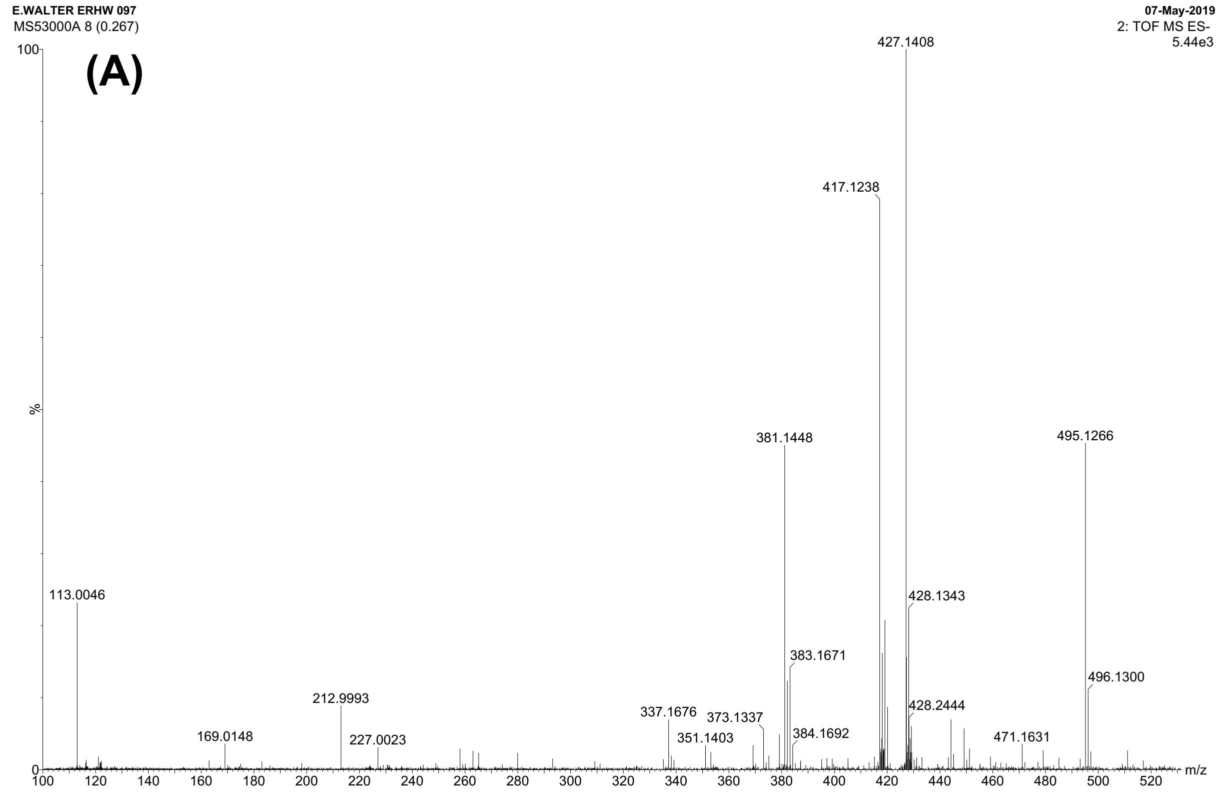


**(B)**

Elements Used:

C: 21-21 H: 0-100 N: 0-10 O: 0-10 Na: 0-1 I: 0-3

| | | | | | | | |
|---|---|---|---|---|---|---|---|
| Minimum: | | | | -1.5 | | | |
| Maximum: | | 5.0 | 10.0 | 100.0 | | | |
| Mass | Calc. Mass | mDa | PPM | DBE | i-FIT | i-FIT (Norm) | Formula |
| 381.1448 | 381.1450 | -0.2 | -0.5 | 12.5 | 81.3 | 0.0 | C21 H21 N2 O5 |

**Figure S40. (A)** The ESI-MS of **(3)** and **(B)** the calculated and found HRMS data.

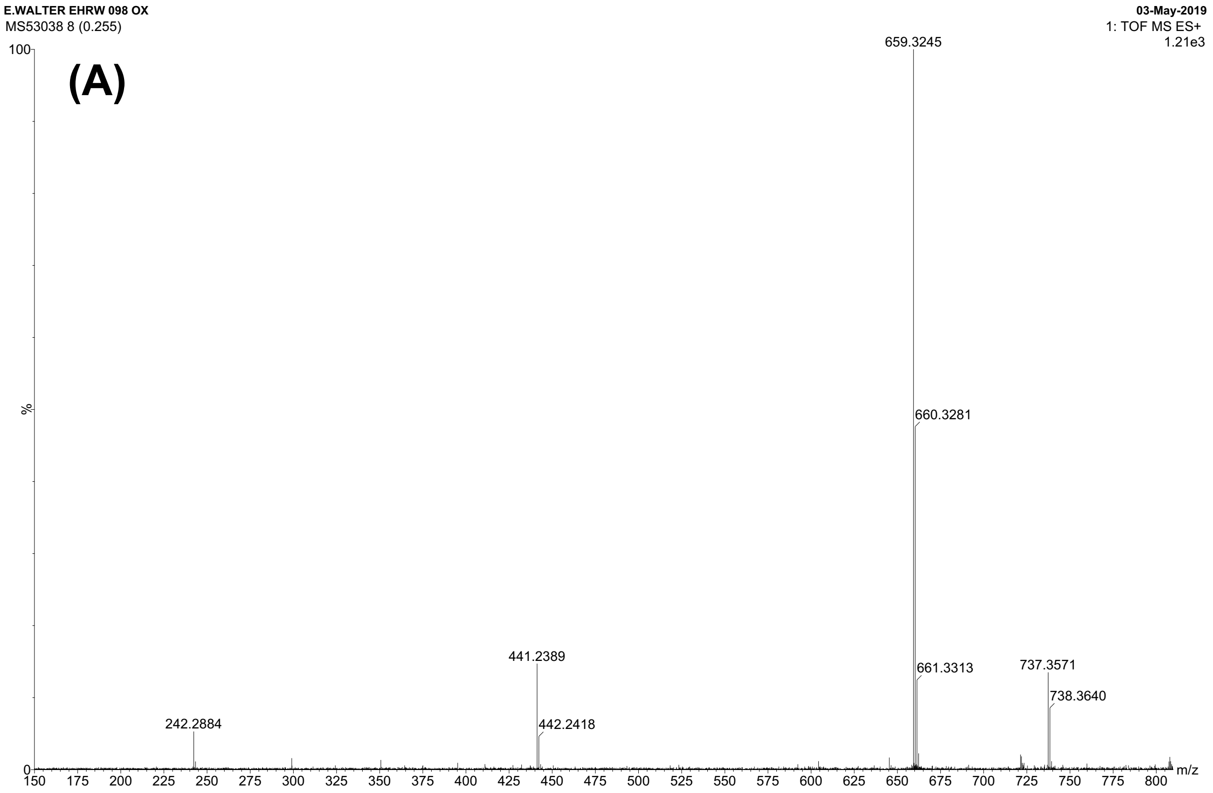


**(B)**

Elements Used:

C: 40-40 H: 0-100 N: 0-10 O: 0-15 Na: 0-1

| | | | | | | | |
|---|---|---|---|---|---|---|---|
| Minimum: | | | | -1.5 | | | |
| Maximum: | | 5.0 | 10.0 | 100.0 | | | |
| Mass | Calc. Mass | mDa | PPM | DBE | i-FIT | i-FIT (Norm) | Formula |
| 659.3245 | 659.3233 | 1.2 | 1.8 | 21.5 | 76.4 | 0.0 | C40 H43 N4 O5 |

**Figure S41. (A)** The ESI-MS of **(6)** and **(B)** the calculated and found HRMS data.

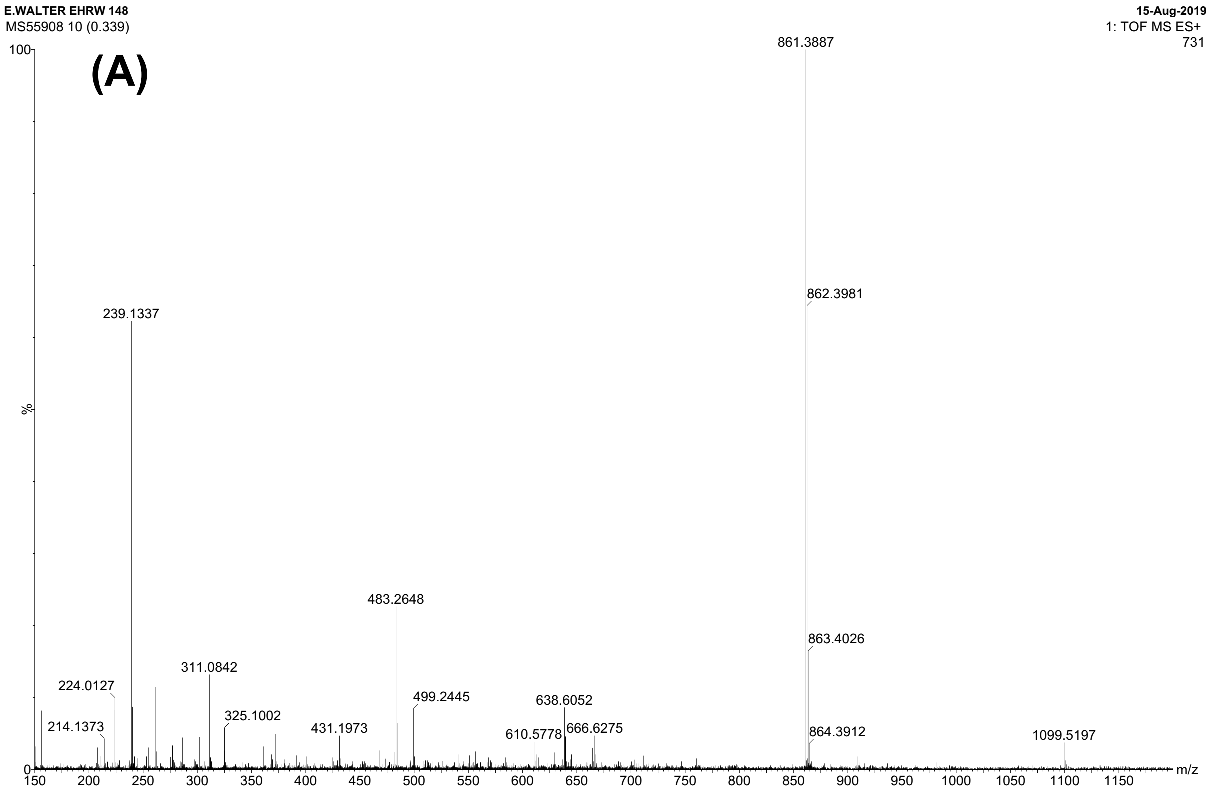


**(B)** Elements Used:

C: 52-52 H: 0-150 N: 0-10 O: 0-10 Na: 0-1

| | | | | | | | |
|---|---|---|---|---|---|---|---|
| Minimum: | | | | -1.5 | | | |
| Maximum: | | 5.0 | 10.0 | 100.0 | | | |
| Mass | Calc. Mass | mDa | PPM | DBE | i-FIT | i-FIT (Norm) | Formula |
| 861.3887 | 861.3863 | 2.4 | 2.8 | 28.5 | 37.7 | 0.0 | C52 H53 N4 O8 |

**Figure S42. (A)** The ESI-MS of **L[1]–DME** and **(B)** the calculated and found HRMS data.

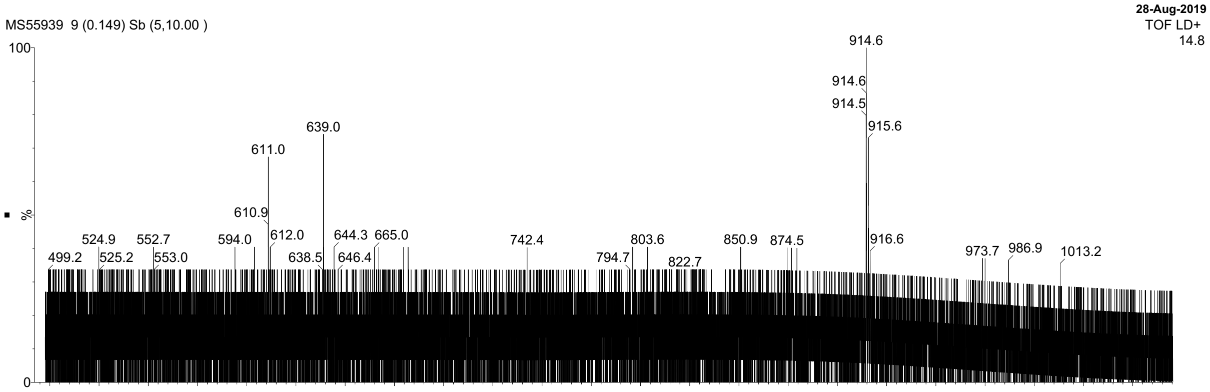


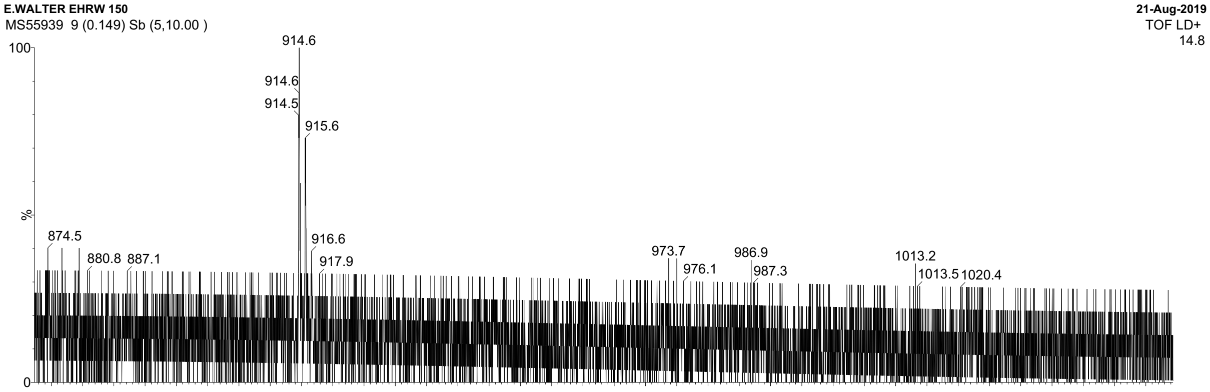


**Figure S43.** The MALDI-MS of **Fe–L[1]–DME**. $[M+H]^+$ = 914.

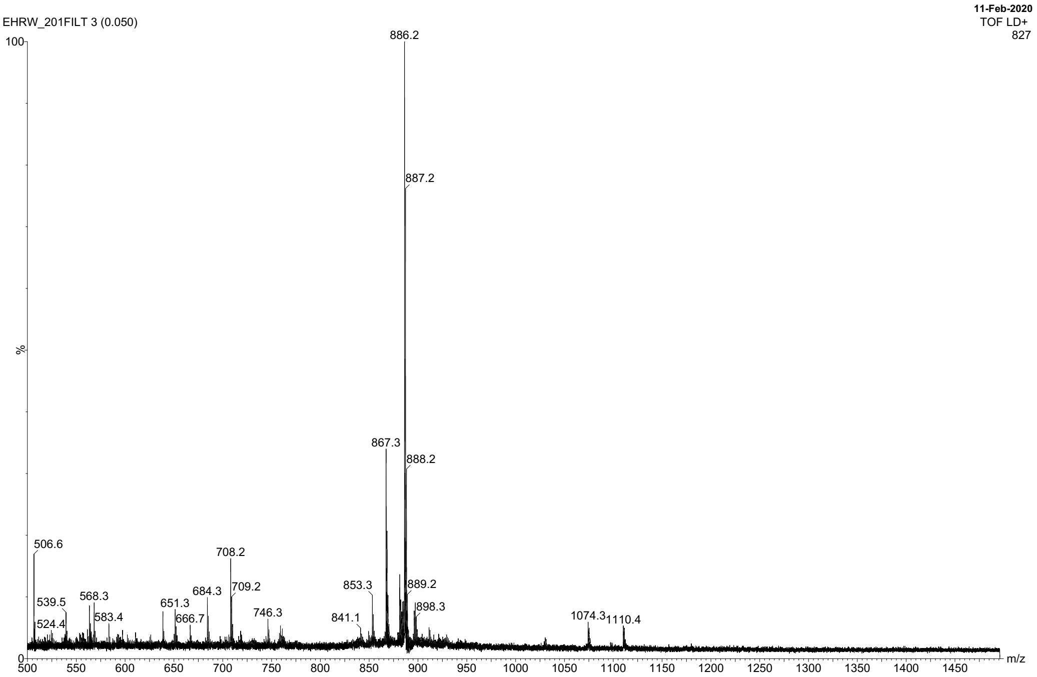


**Figure S44.** The MALDI-MS of **Fe–L[1]**. $[M+H]^+$ = 886.

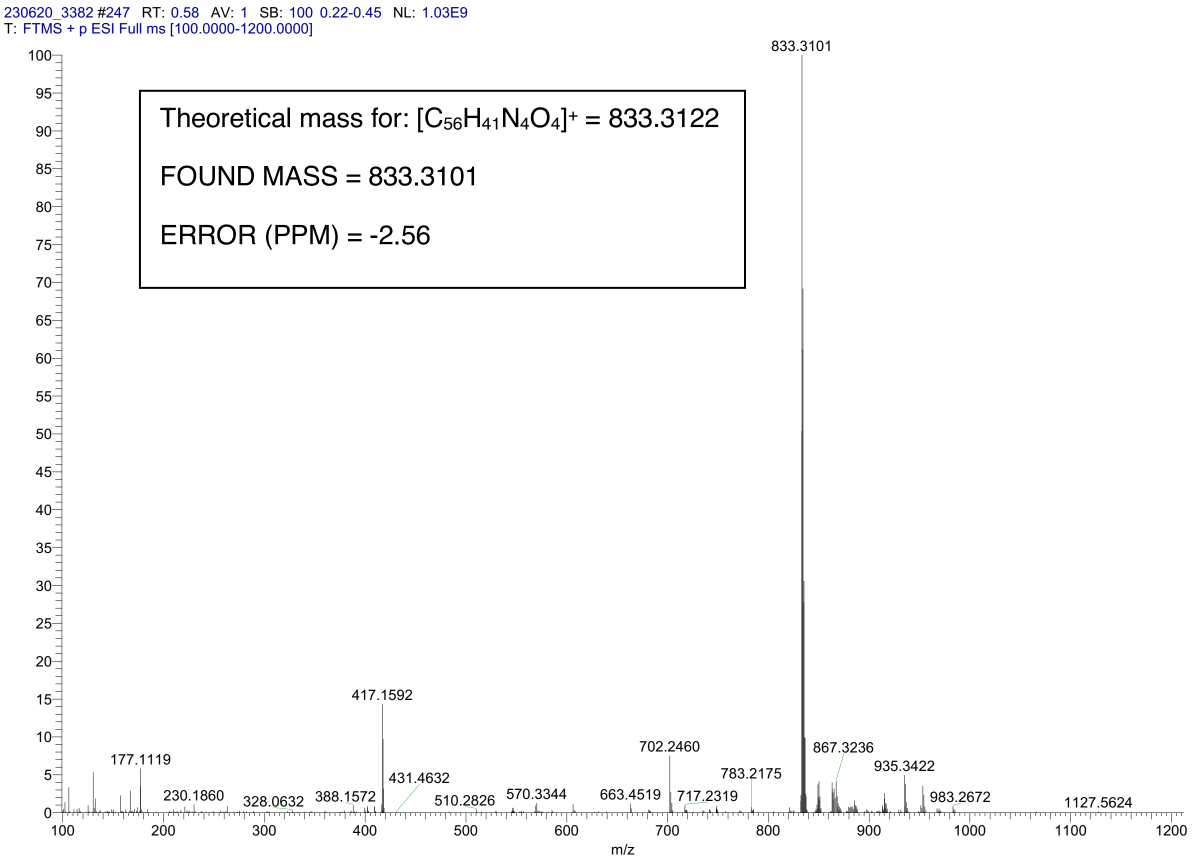


**Figure S45.** The ESI-MS and HRMS (inset) for **L²**.

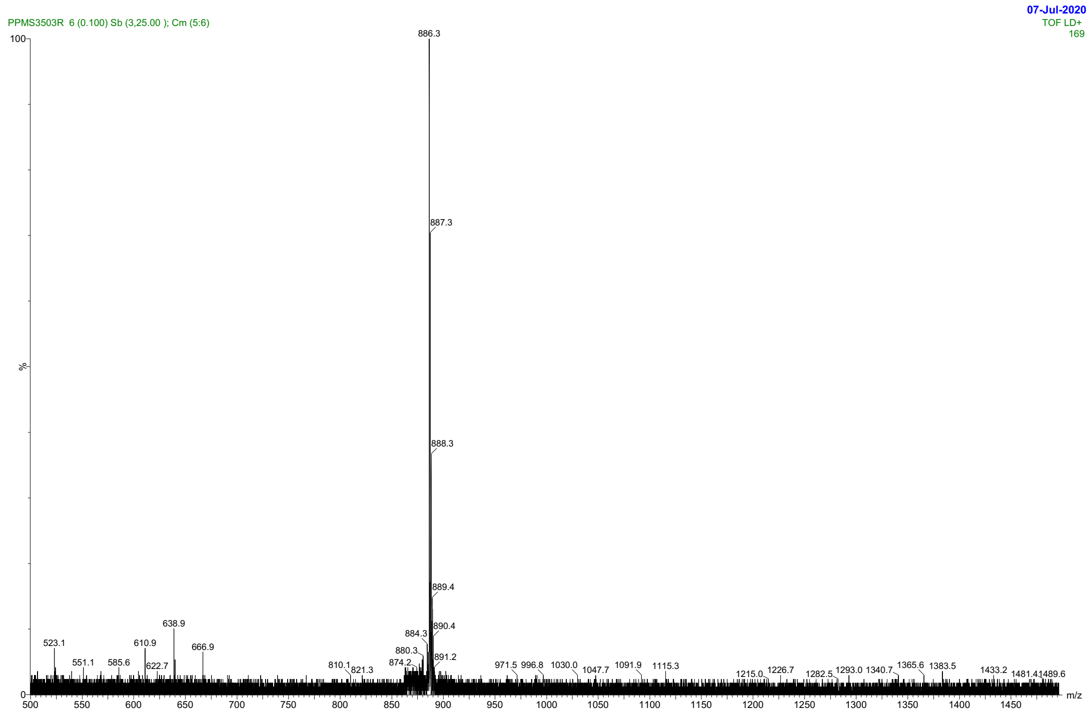


**Figure S46.** The MALDI-MS of **Fe–L²**. $[M+H]^+ = 886.3$.

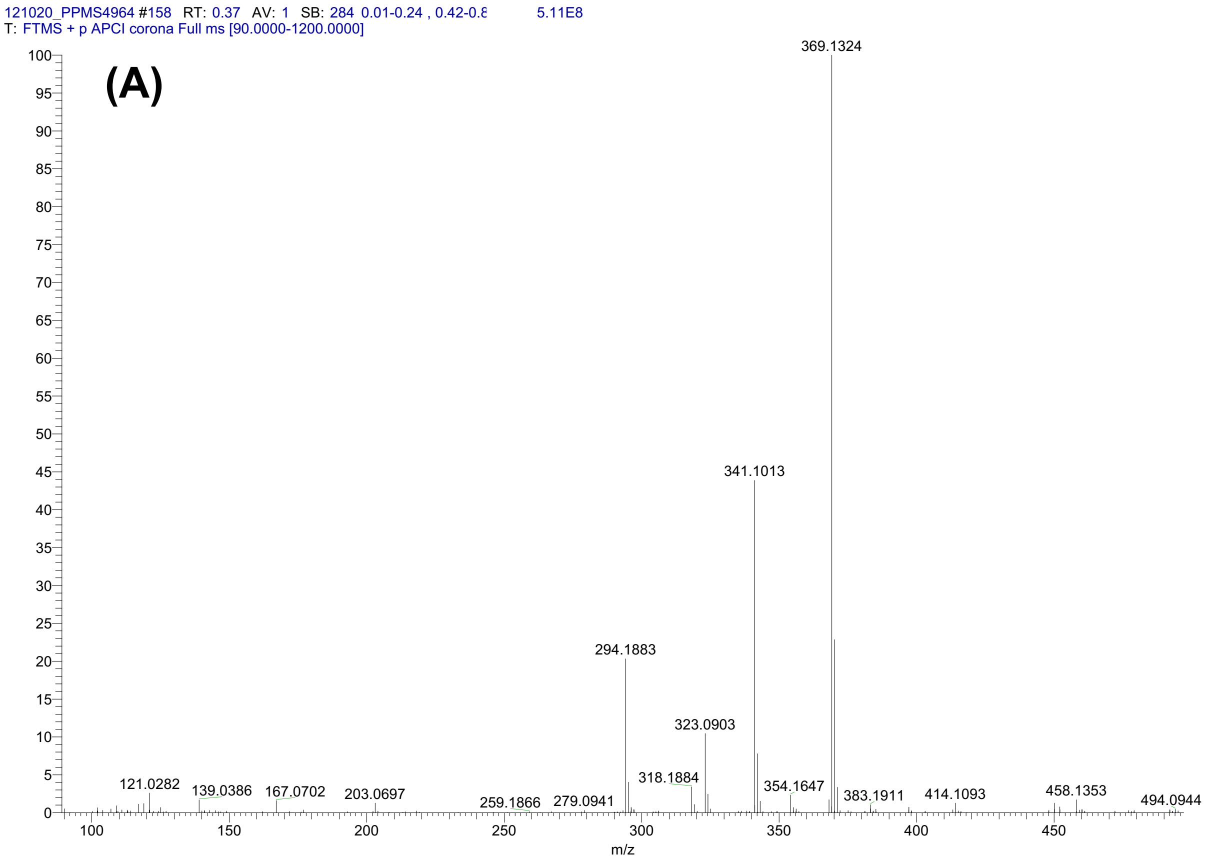


(B)

THEORETICAL MASS FOR C21H21O6+ = 369.1333

FOUND MASS = 369.1324

ERROR (PPM) = -2.34

**Figure S47. (A)** The ESI-MS of **(11)** and **(B)** the calculated and found HRMS data.

(A)

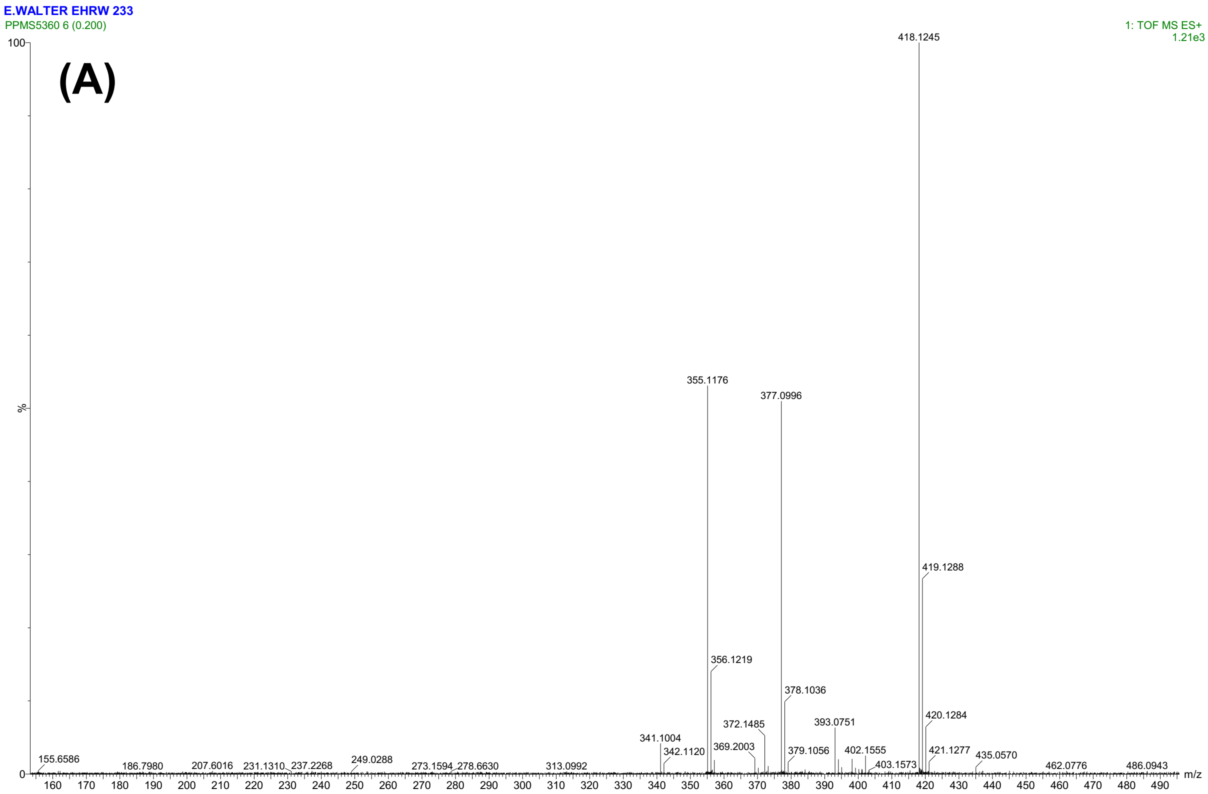


(B)

Elements Used:

C: 20-20 H: 0-150 N: 0-10 O: 0-10

| | | | | | | |
|---|---|---|---|---|---|---|
| Minimum: | | | | -1.5 | | |
| Maximum: | | 5.0 | 10.0 | 1000.0 | | |
| Mass | Calc. Mass | mDa | PPM | DBE | i-FIT | Formula |
| 355.1177 | 355.1182 | -0.5 | -1.4 | 11.5 | 0.2 | C20 H19 O6 |

**Figure S48. (A)** The ESI-MS of **(12)** and **(B)** the calculated and found HRMS data.

## 4. References


(1) Yields, Q. XIII': Fluorescence Spectra and Quantum Yields PAUL G. SEYBOL~~ AND NARTIN GOUTERMAN. *Quantum* **1969**, *13*, 1–13.

(2) Lash, T. D.; Bellettini, J. R.; Bastian, J. A.; Couch, K. B. Synthesis of Pyrroles from Benzyl Isocyanoacetate. *Synthesis (Stuttg).* **1994**, 170–172.

(3) Martin, P.; Mueller, M.; Flubacher, D.; Boudier, A.; Blaser, H. U.; Spielvogel, D. Total Synthesis of Hematoporphyrin and Protoporphyrin: A Conceptually New Approach. *Org. Process Res. Dev.* **2010**, *14*, 799–804.

(4) Jiang, N.; Huang, Q.; Liu, J.; Liang, N.; Li, Q.; Li, Q.; Xie, S. S. Design, Synthesis and Biological Evaluation of New Coumarin-Dithiocarbamate Hybrids as Multifunctional Agents for the Treatment of Alzheimer's Disease. *Eur. J. Med. Chem.* **2018**, *146*, 287–298.

(5) Osswald, P.; You, C. C.; Stepanenko, V.; Würthner, F. DABCO-Mediated Self-Assembly of Zinc Porphyrin-Perylene Bisimide Monodisperse Multichromophoric Nanoparticles. *Chem. - A Eur. J.* **2010**, *16* (8), 2386–2390.
